# Conformational Investigations in Flexible Molecules Using Orientational NMR Constraints in Combination with ^3^*J*-Couplings and NOE Distances

**DOI:** 10.3390/molecules24234417

**Published:** 2019-12-03

**Authors:** Christophe Farès, Julia B. Lingnau, Cornelia Wirtz, Ulrich Sternberg

**Affiliations:** 1 Max-Planck-Institut für Kohlenforschung, Kaiser-Wilhelm Platz 1, 45470 Mülheim an der Ruhr, Germany; fares@kofo.mpg.de (C.F.); lingnau@kofo.mpg.de (J.B.L.); wirtz@kofo.mpg.de (C.W.); 2 Research Partner of Karlsruhe Institute of Technology, 76131 Karlsruhe, Germany; 3 Present address: COSMOS-Software GbR, 07743 Jena, Germany

**Keywords:** NMR, molecular dynamics, residual dipolar couplings, tensor-free

## Abstract

The downscaling of NMR tensorial interactions, such as dipolar couplings, from tens of kilohertz to a few hertz in low-order media is the result of dynamics spanning several orders of magnitudes, including vibrational modes (~ns-fs), whole-molecule reorientation (~ns) and higher barrier internal conformational exchange (<ms). In this work, we propose to employ these dynamically averaged interactions to drive an “alignment-tensor-free” molecular dynamic simulation with orientation constraints (MDOC) in order to efficiently access the conformational space sampled by flexible small molecules such as natural products. Key to this approach is the application of tensorial pseudo-force restraints which simultaneously guide the overall reorientation and conformational fluctuations based on defined memory function over the running trajectory. With the molecular mechanics force-field, which includes bond polarization theory (BPT), and complemented with other available NMR parameters such as NOEs and scalar *J*-couplings, MDOC efficiently arrives at dynamic ensembles that reproduce the entire NMR dataset with exquisite accuracy and theoretically reveal the systems conformational space and equilibrium. The method as well as its potential towards configurational elucidation is presented on diastereomeric pairs of flexible molecules: a small 1,4-diketone **1** with a single rotatable bond as well as a 24-ring macrolide related to the natural product mandelalide A **2**.

## 1. Introduction

Tensorial NMR observables, such as chemical shift anisotropy, dipole-dipole couplings or nuclear quadrupolar splittings, give access to structurally-relevant local orientation at nuclear sites relative to the external magnetic field. This information can also be retrieved in solution at high-resolution by intentionally imposing an anisotropy to the otherwise Brownian molecular reorientation. Since their first description in solution [1], such interactions have been used to understand structure and dynamic properties of small molecules dispersed in liquid-crystalline solvents [2]. In the last two decades, a number of media have been proposed that scale these interactions down to small perturbations on the order of a few Hz which are as easy to evaluate as scalar couplings. These structure-dependent “residual” dipolar couplings (RDCs) or “residual” chemical shift anisotropy (RCSAs), when taken together, report on the geometry of the molecule as a whole, complementing the more commonly-used, exclusively short-range observables, such as chemical shifts (CS), nuclear Overhauser enhancements (NOEs) and scalar (or *J-*) couplings [3,4]. Whereas, for biological systems, these tensorial interactions have been applied towards validating and refining folds [5] as well as studying dynamic phenomena [5,6,7], they have recently found popularity, especially in organic solvents, in their application towards solving stereochemical problems, such as enantiodifferentiation and stereochemical elucidations [8,9]. Indeed, RDCs and RCSAs offer a simple and attractive approach aimed at relatively rigid molecules for which the available conformational space does not deviate significantly from a mean structural representation [10,11,12]. In the most typical approach, the measured RDCs < *D_i_ >* are joined in a set of linear equations (e.g., SVD [13]) to determine an order matrix **A [14]** which describes the alignment of a molecular fixed frame relative to the static magnetic field and which enables the back-prediction of all RDCs from this structure model [8,15,16,17]. Despite the structural assumptions and the often “under-determination” of **A**, this method can deliver clear-cut validation of stereochemistry even with imperfect correlation.

Flexible molecules can typically exhibit a wide range of conformers, whose determination is a challenging problem, relevant to many fields of molecular sciences including medicinal and catalytic chemistry. The interconversion between conformational states, fast on the NMR timescale (Hz-kHz), leads to the averaging of NMR parameters. Whereas a mean value for the short-range parameters CS, NOEs and *J-*couplings can often be interpreted semi-quantitatively by means of local flexibility (e.g., confined fluctuations, rotameric exchanges, ring-flips), the interpretation of averaged RDCs is a much more complex undertaking. For one, the non-linearity and ambiguity of the RDC solutions combined with the potentially large number of degrees of freedom between distant interatomic vectors across a molecule restrict their interpretation based on “simple local fluctuations”. More importantly, RDCs are parameters that are dynamically-averaged over time-scales spanning several orders of magnitude and so “whole-molecule” anisotropic reorientation cannot be clearly disentangled from faster vibrational modes and from slower internal geometrical interconversion. In this regard, a relation between *D_i_* and an order matrix **A** fixed within the molecular frame becomes much more ill-defined. Though some level of success can be achieved using a small set of minimized geometries combined with an overall average alignment tensor or with discrete number of order tensors [10,18], these methods cannot be generalized to all flexible molecules, encompassing all dynamic amplitudes, timescales and complexity. Besides the steep increase in complication in fitting data in this way, one additional challenge here is to properly access the full conformational space.

The method of choice to survey the entropically driven conformational space remains all-atom molecular dynamics (MD) simulations. Generally speaking, an accurate simulation should be conducted in the presence of solvent molecules (e.g., a “solvent box”), since all energy states and barrier heights are potentially influenced by the environment. The complexity of such simulations would be amplified if more intricate environments such as oriented media like stretched gels or biomembranes, used in NMR experiments to extract (R)DCs, are to be included, and thus reaching conformational equilibria would become unrealistically time consuming. Moreover, highly elaborate force fields are needed to calculate proper interaction energies between the solvent and the molecules under investigation. Therefore it is clear that traditional all-atom MD simulations cannot bridge the gap of several orders of magnitude between the MD time-scale (up to µs) and that of the equilibria governing NMR (up to ms).

One way out of this conundrum is restrained MD simulation methods that are guided by NMR parameters. Not surprisingly, NMR parameters including tensorial interactions—such as RDCs and RCSAs have been used within MD simulations to bias structural fluctuations and to actively drive the simulation towards conformational solutions. Besides the commonly used simulated annealing and refinement to optimize molecular geometries based on NMR information [19,20,21,22], a number of other RDC-driven methodologies have been presented for ensemble simulations of flexible systems [6,23,24,25,26,27].

All RDC-restrained MD methods generally share some implicit assumption about the stochastic reorientation order of the molecule. Most commonly, this takes the form of the Saupe order tensor A requiring 5 independent terms (a 3 × 3 matrix expressing a symmetric traceless tensor or more intuitively, the magnitude A, the rhombicity R and the 3 rotation Euler angles).

One departure from this approach is the so-called tensor-free ϑ-method, where the averaged second-order Legendre polynomial < P_2_(cos ϑ) > relating the RDC internuclear vector to the laboratory frame is used to restrain a replica-averaged MD simulation [28]. The appeal of this approach is that the approximations and assumptions associated with the determination of an alignment tensor is avoided. Instead, the orientational bias is calculated along with the conformational fluctuations within the restrained MD simulation. Still, the implicit overall scaling of the dipolar coupling clearly marks a delineation between the whole-molecule reorientation and the internal fluctuations. Using a bias-exchange metadynamics approach, the authors illustrated the method on the small molecule, strychnine, to determine its major conformational states and populations based on RDCs [29].

Most of these restrained MD methods generally share a common aspect—they restrain only the *zz*-component of the interaction tensor [23] or the parameter P_2_(cos ϑ) derived from that component [28] with respect to the *z*-direction of the external magnetic field (directly or via the director of the sample alignment). In reality, the full dynamics of the molecular motion requires that the time and ensemble mean value of the tensor’s off-diagonal elements vanish. Consequently, using all elements of the dipolar coupling second-order tensor (rather than only their scalar values) as a MD-restraint should autonomously drive the full internuclear orientational dynamics.

Molecular Dynamics with Orientational Constraints (MDOC) simulations provide an alternative approach which uses tensorial NMR parameters as constraints [30,31] and which requires no alignment or order tensors. This approach was recently introduced and thoroughly investigated towards conformation and configuration determination on small molecules, from relatively rigid ring-systems to system with some level of internal flexibility, using only RDCs [32,33]. The MDOC method exhibits several important distinctions from the aforementioned MD methods [23,28,29]. For one, the expression of the RDC-based restraint is extended to all 5 independent components (i.e., including off-diagonal components) of its tensor description; thus, in contrast to a scalar restraint, the downscaling of the interaction over several orders of magnitude is in large part explicitly driven within the simulation as internal geometrical fluctuations and overall whole-molecule reorientations. Second, the averaging of the parameters is considered ergodic and takes place over the course of the trajectory using an adjustable memory function. This results in a type of “accelerated” MD towards understanding conformational equilibria and is independent of the initial structure. Finally, the MDOC molecular mechanic force-field includes the elaborate bond polarization theory (BPT) which calculates electrostatic energy along the trajectory, and is extendable with other easily accessible NMR parameters, such as chemical shifts, *J*-couplings and NOEs which can be also applied as time-averaged constraints.

With its attributes, this light-weight yet rich MD platform, available within the COSMOS package, allows the bypass of explicit solvent or aligned media, since their influence is embodied within the reorienting pseudo-forces. Together, the features of MDOC present the double advantage of considerably simplifying the complexity of the MD as well as accelerating the overall reorientation and internal fluctuations giving rapid access to a broad conformational space and conformational equilibria.

In this paper, a key advance is presented in the application of MDOC on small and flexible organic chiral compounds based on carefully evaluated RDCs. It is shown that MDOC converges to ensembles with satisfying results on small flexible molecules when complemented with other NMR-based parameters such *J-*coupling and NOEs. As a first simple example, simulations on a diastereomeric pair of 1,4-diketone [34] **1** were undertaken to generate rotamer distributions. The much more complex second example uses a pair of stereoisomers of mandelalide A, a 24-ring macrolactone including nine main-chain chiral centers [35] for which a rich set of NMR data was available. These examples validate the MDOC approach towards generating realistic thermodynamic structural ensemble and show encouraging potential towards guiding the correct configuration assignment.

## 2. Results and Discussion

### 2.1. Single-Bond Dynamics in Diketone ***1***

#### 2.1.1. NMR Datasets

**Figure 1:**
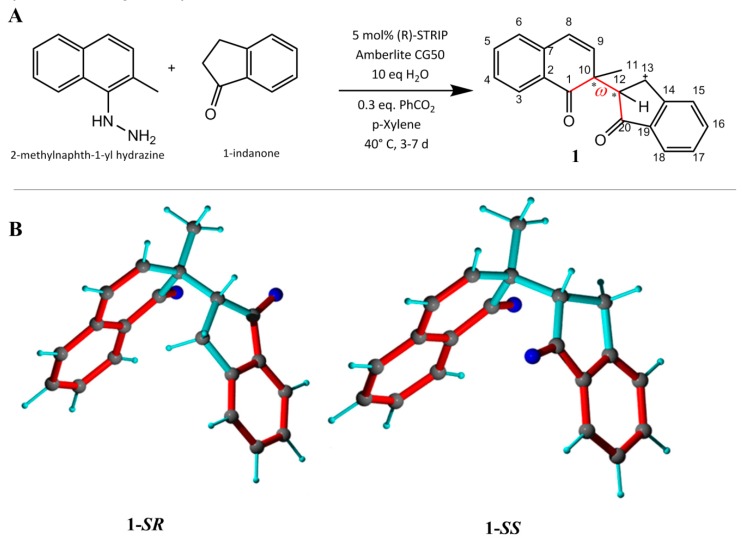
(**A**) Synthesis of **1 [34]**. (**B**) The **1-*SR*** (C10-S and C12-R) and **1-*SS*** (C10-S and C12-S) forms. Bond rotation (defined by torsion angle ω (C1-C10-C12-C20), red) is not restricted. Conjugated double bonds are displayed in red and single bonds in cyan.

For the first assessment of the MDOC approach, a simple flexible chiral molecule was required. For this task, the 1,4-diketone **1** was synthesized as a coupling product between 2-methylnaphth-1-yl hydrazine and 1-indanone in an asymmetric Brønsted acid catalyzed dearomatizing redox cross coupling reaction [34] (Figure 1). Characteristic for this molecule are the two chiral and relatively rigid subgroups (a naphthalenone group and an indenone group) connected by a single rotatable bond. The modest stereoselectivity (dr < 2.5) during the trials of this reaction resulted in both diastereomers of the 1,4-diketone **1** being available for this study. The labels **1-*SR*** and **1-*SS*** are applied henceforth to represent the two diastereomers, differing only in the chirality at carbon C12, for simplicity. The diastereomers were separated by chromatography and provided two samples for dynamic investigation by NMR.

The theoretical rotamers (*trans*, *gauche* (-) and *gauche* (+)) of **1** were first geometry-optimized and their relative energies calculated using DFT (see Supporting Information). Table S1 shows that the *trans* and the *gauche* (-) geometries are the most stable among the conformers of **1-*SS*** and **1-*SR***, respectively, which both correspond to the configuration where the small indenone H12 is simultaneously *gauche* to the bulkier methyl (C11) and carbonyl (C1) groups. Nevertheless, the other rotamers should also be accessible, especially for **1-*SR*** with all three relative energies within 1 kcal/mol. For **1-*SS***, the theoretical differences are somewhat larger (E*_tr_* − E*_g+_* = 3.6 kcal/mol) due to the combination of the repulsive effect of the ketones and overall steric effect of the other 2 rotamers. It is expected however that these differences could be mitigated by the polar solvent. These optimized structures provide already theoretical evidence for significant rotameric exchange and will serve also as starting structures for the dynamical evaluations.

The NMR investigation provided two datasets (1A **(1-*SS*)** and 1B for (**1-*SR***)) comprising carefully evaluated NOESY cross-peaks integrals and converted to interproton distances and a set of ^3^*J*-coupling constants related to torsion angle distribution (for datasets, see Supplementary Information). Both the measured NOE contacts as well as the homo- and heteronuclear ^3^*J* couplings in the initial NMR analysis indicate some level of rotameric exchange, fast on the NMR timescale. For instance, there are no distinctly large ^3^*J_CH_* couplings (>9 ppm) from H12 to C1, C9 or C11 across the rotatable C10-C12 bond that would clearly hint to a static *trans* relation. Furthermore, the semi-quantitative evaluation of the interannular NOEs from H13 or H12 to H11 or H9 show that all pairs correlate with NOEs of similar magnitudes which also imply some form of dynamic averaging.

The datasets were extended with HH and CH RDC values (22 in the case of 1A and 24 for 1B), obtained from a set of coupled experiments in the isotropic (CDCl_3_) and the anisotropic (PMMA/CDCl_3_) sample for each diastereomer. An RDC-fitting procedure using MSPIN (SVD) against the corresponding three DFT rotamers identified the 1A and 1B dataset to fit best to **1-*SS-tr*** (*Q* = 0.19) and **1-*SR*-*g(-)*** (*Q* = 0.21) conformation, respectively (see Supporting Information). These rotamers match with those with the lowest calculated DFT energies. However, the quality of the best fits were less than optimal as indicated by the quality criterion (n/χ^2^) and outlier criterion (1/χ^2^_min_) far below unity (1A: n/χ^2^ = 0.07, 1/χ^2^_min_ = 0.013 and 1B: n/χ^2^ = 0.05, 1/χ^2^_min_ = 0.006), as well as only ℱ = 6/22 (27%) and 5/24 (21%) of the computed RDCs being valid, i.e., having values within the estimated error margins. These observations indicate that overall, a majority of RDCs cannot be accurately rationalized by a lone rotamer.

The attempts to fit RDCs to a multi-rotameric distribution with a common averaged alignment tensor lead to a substantial improvement in the fitting (1A: *Q* = 0.16 and 1B: *Q* = 0.14), however the above mentioned criteria are still less than satisfactory (1A: n/χ^2^ = 0.11, 1/χ^2^_min_ = 0.01 and ℱ = 41% (9/22) and 1B: n/χ^2^ = 0.08, 1/χ^2^_min_ = 0.015 and ℱ = 54%(13/24)). It should be mentioned that these only modest improvements may be in part due to the assumption of an existing average alignment tensor; indeed, since the overall shape of the molecule changes substantially from one rotamer to the other, a strong dependence of the alignment to the rotameric state is to be expected. Unfortunately, the sparseness of the RDC dataset did not allow the use of *multiple* alignment tensors since this procedure would have required at a minimum 17 independent RDC values (3 × 5 for the Saupe (alignment) matrix + 2 for the three populations). A further extension of the dataset (e.g., with less accessible long-range HC RDC couplings) would not have improved the required low condition number since a large portion of the RDC vector orientations are confined within or close to the aromatic planes and are therefore not completely independent. The inclusion of the NOE interproton information and the ^3^*J*-coupling (CH) in the stereofitter module of MSPIN did not give significantly better results (data not shown).

The example of the diketone **1** demonstrates, as for many molecules previously studied in past, that the linear fitting approaches like SVD constitute a powerful tool to roughly discriminate structures especially between a finite number of possible conformers; however, as a molecule exhibits increasingly more disorder, the method becomes progressively more deficient towards accurately explaining dynamic exchange. The aim of the MDOC approach introduced in the next section therefore presents a novel tool to obtain an ensemble of structures based on NMR observables that describe the conformational space for each diastereomer.

#### 2.1.2. MDOC Simulation

Two MDOC simulations were performed combining the structure configurations **1-*SR*** and the **1-*SS*** with their corresponding NMR constraints from datasets 1A and 1B (for the details of the simulations see Tables S10 and S11 in Supplementary Information). Datasets 1A and 1B included 1-bond CH RDCs (1A:14, 1B:14) and long-range HH RDCs (1A:8, 1B:10)), NOEs (1A: 7, 1B:5), *3J*-couplings (1A: 5, 1B:5), each translated into a pseudo-force term for the MDOC simulations. The outcome was independent of the initial conformation, since the pseudo-forces calculated from the RDC have the ability to rapidly generate all possible rotamers as well as reorient the molecule as a whole, on a timescale related to the memory time *τ* of the mean value calculation (in this case 200 ps, Equation (3)).

**Figure 2:**
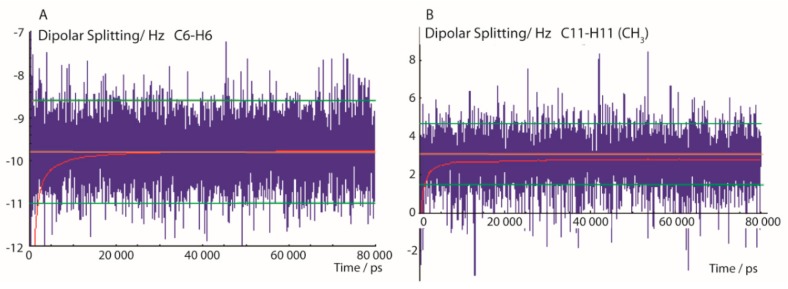
Selected ^13^C-^1^H dipolar splittings of an 80-ns MDOC simulation of the **1-*SS*** configuration with dataset 1A. The trajectories display the mean values with exponential memory according to Equation (3) (blue) and the running mean over the former values (red). Extreme values at beginning of the trajectory are later cut off. The orange line indicates the experimental splitting and the lower and upper error bounds are indicated by green lines. (**A**): C_6_-H_6_ bond dipolar splitting and (**B**): C-H splitting of the CH_3_ group at position C11.

In Figure 2, typical trajectories of ^13^C-^1^H dipolar splittings are displayed. The left panel shows the fluctuation of the dipolar coupling of the aromatic C-H group at position C6 calculated using the exponential function with a memory time *τ* of 200 ps, according to Equation (3). After a short simulation time of about 1 ns—approximately 5 times the exponential rise constant of the pseudo-forces—the time mean value of the dipolar coupling fluctuates mostly within a range of about ±1 Hz. The experimental error of 1.2 Hz is indicated in Figure 2 with green lines. The pseudo-force width constant ΔD (Equation (5)) was set to 0.5 Hz which is close to the error ranges of most experimental values. Figure 2 also shows the convergence of the running mean value (red) of the trajectory towards a value close to the experimental RDC and reaching a constant value after about 10 ns, indicating that this MDOC simulation period was sufficient to average the dipolar coupling through combined whole-molecule reorientation and internal dynamics.

In statistical analysis of traditional MD simulations, the initial time period before the system reaches a thermal equilibrium is often disregarded. The state of equilibrium in MDOC is reached when the mean values with exponential memory fluctuate only within the experimental error bounds, mostly after about 1 ns. Flowingly, the first nanosecond in the final analysis is dropped according to the decay function described in Equation (3).

The right panel of Figure 2 displays the interesting case of the CH_3_ group which involves one additional mode of motion—the axial reorientation of the CH_3_ group. Though the local order parameters for freely rotating methyl groups are typically much smaller than in the case of the aromatic CH groups, the conditions for a successful convergence are also fulfilled within these time limits.

**Figure 3:**
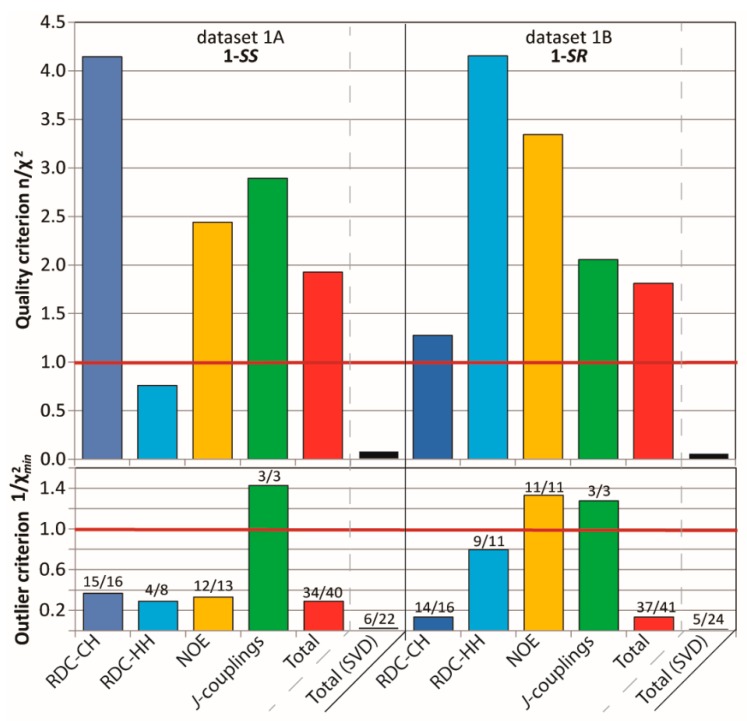
criterion n/χ^2^ (Equation (7)), “smallest outlier” criterion 1/χ^2^_min_ and fidelity ℱ (labels) for MDOC simulations of the 1,4-diketone ***1-SS*** and **1-*SR*** with the experimental datasets 1A and 1B, respectively. On average, all interaction types are very well reproduced by ensembles, with n/χ^2^ > 1 (overall 1A: 1.9, 1B: 1.8) and fidelity ℱ above 85% (overall 1A: 34/40, 1B: 37/40).

Figure 3 shows that the running ensembles of the MDOC simulation for both **1*-SS*** and **1-*SR*** are in good accord with the entire corresponding measured datasets, which include four categories of NMR parameter types (one-bond and long-range RDCs, *^3^J-*couplings and NOEs). In stark contrast to the outcome of the linear analysis based only on RDC values fit to static rotameric DFT models, the aforementioned quality criterion (n/χ^2^) exceeds unity for all but one parameter type, the only exception being the “long-range” (*^n^D*, *n* > 1) RDCs for the **1-*SS*** configuration with n/χ^2^ = 0.76 (Figure 3). This exception is specifically affected by the calculated H6-H8 coupling, with a deviation of 1.4 Hz from the measured RDC, whose error was estimated to be ± 0.9 Hz. In the case of the MDOC simulation of the ***1-SS*** and ***1-SR*** configurations with dataset 1A and 1B, there are 6 (ℱ = 34/40 (85%)) and 4 (ℱ = 37/41 (90%)) outliers observed, with $\chi_{min}^{-2}$ values of 0.29 and 0.13 respectively (see supplementary information). Though these numbers represent a large improvement over the corresponding values in the static SVD study (black in Figure 3: 1A: #outliers = 13/22 and $\chi_{min}^{-2}$ = 0.015; 1B: #outliers = 11/22 and $\chi_{min}^{-2}$ = 0.010), it will be important to determine the factors contributing most to the remaining incongruity.

**Figure 4:**
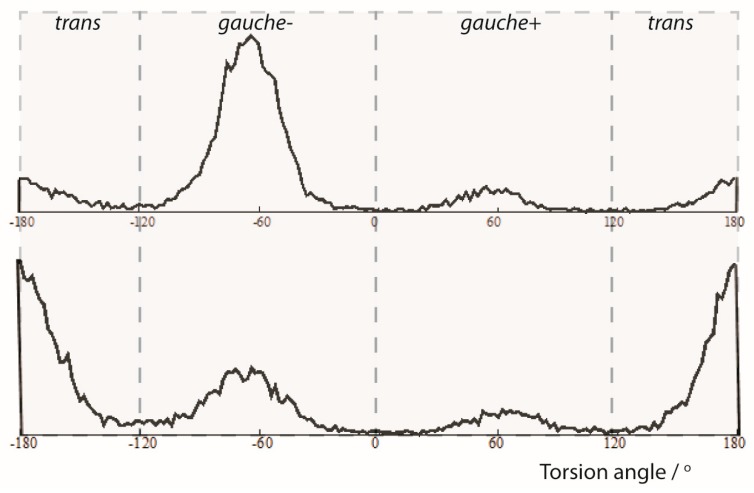
Angle analysis of the C1-C10-C12-C20 torsion angle (ω) from an MDOC simulation of the **1-*SR*** and **1-*SS*** form of the 1,4-diketon. Upper panel: **1-*SR*** form: torsion statistics for the ω torsion angle. The {*trans, gauche(-), gauche(+)*} ratios are {0.165, 0.730, 0.105} Lower panel: **1-*SS*** form: torsion statistics for the ω torsion angle. The {*trans, gauche(-), gauche(+)*} ratios are {0.617, 0.281, 0.101}.

The analysis of the trajectories from the MDOC simulations also provided insight into the population of conformers. In Figure 4, the torsion angle ω distributions for the central C10-C12 bond are presented. Clearly, the three maxima represent the accessible rotameric states of **1** corresponding to *trans, gauche(-) and gauche(+)* subensembles. In the **1-*SS*** form the *trans*-conformation is clearly favored whereas in the ***1-SR*** form the *gauche(-)* conformation is the dominant conformation. This is in general accordance with the relative DFT energies from the geometry optimized DFT models and with the results of the SVD analysis (see Supporting Information). More specifically, however, a statistical analysis showed that the relative population of the individual rotameric states ratios (*trans*:*gauche(-)*:*gauche(+)*) are more balanced for the minor components (**1-*SS***: {0.617: 0.281: 0.101} and ***1-SR*** {0.165: 0.730: 0.105}

**Figure 5:**
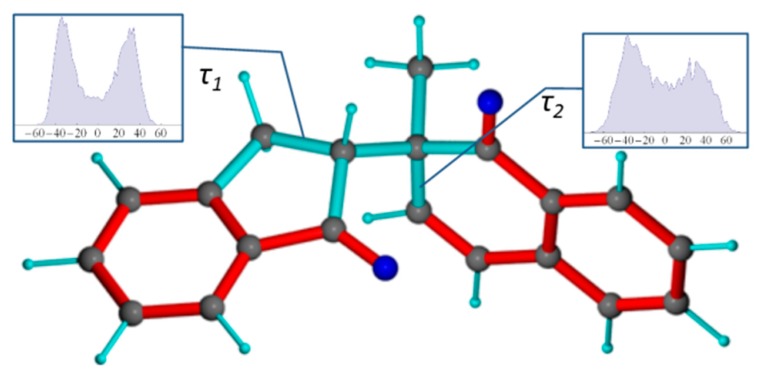
Conformations of the ***1-SR*** 1,4-diketone (displayed in the preferred *gauche(-)* conformation). Right inset diagram: torsion angle distribution of $\tau_{1}$ (C8-C9-C10-C1 in Figure 1). Left inset diagram: torsion angle distribution of $\tau_{2}$ (C14-C12-C13-C20 in Figure 1).

Besides the rotation about the C10-C12 bond, the MDOC simulations reveal the presence of conformational isomeric exchange within the non-aromatic rings. Indeed, both rings of the molecules are not fully planar and therefore more than one twist forms of the rings are possible (Figure 5).

The distribution of the C8-C9-C10-C1 ($\tau_{1}$) torsion angle shows maximum puckered forms at −40° and +40° although a continuous distribution of more planar forms are possible as well (see inset panel, Figure 5). More distinct maxima at ±35° are seen for the C14-C12-C13-C20 ($\tau_{2}$) torsion angle within the 5-membered ring indicating a stronger preference of twisted forms. By comparison, the molecular model of ***1-SR*** in *gauche(-)* conformation obtained from a DFT geometry optimization, a static torsion angle of $\tau_{1}$ = −6.4° and $\tau_{2}$ = 13.5° are observed, which are not dominant in the MDOC simulation. This raises the question whether this ring-twist dynamic behavior represented by this ensemble distribution is real or rather an artifact of the MDOC method. For the $\tau_{2}$ dihedral angle, this question could be investigated, by considering the ^3^*J*_HH_ couplings of H13a and H13b to H11. Using the Altona equation [4], the **1-*SR*-*g(-)*** DFT model gives a value of ^3^*J*_H13b-H12_ = 8.5 Hz. The MDOC simulation which was run at a mean temperature of 313 K gave rise to a mean value of 5.8 Hz within the estimated error range (±0.75 Hz) of the experimental value of 5.1 Hz, so that one can suppose that this isomeric exchange occurs also in reality.

### 2.2. Mandelalide A

#### 2.2.1. NMR Datasets

**Figure 6:**
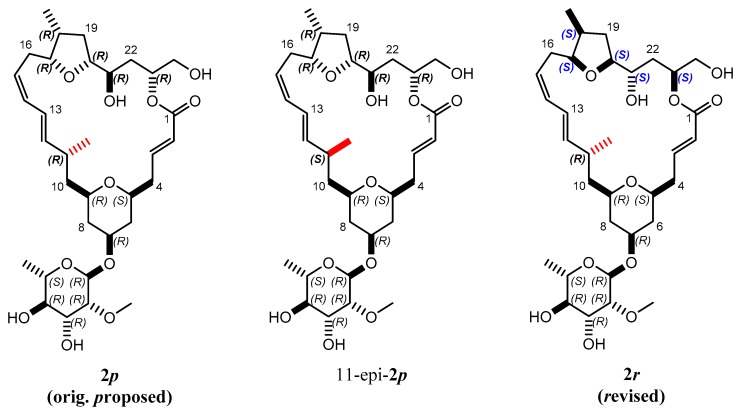
Proposed **2*p*** and *r*evised **2*r*** isomers of the natural product mandelalide A. During the search for the right configuration, 11-epi**-2*p,*** for which the bond indicated in red was inverted, was also synthesized.

Mandelalide A is a glycosylated macrolide with interesting cytotoxic properties found in a species of *Lissoclinum ascidian* (marine invertebrate) from South Africa [35]. This natural product has been the object of two recent total synthetic projects [36,37]. An original configuration of the large lactone ring containing 9 stereocenters was proposed based on scalar coupling constants and interproton NOEs (**2*p*** in Figure 6). The tedious syntheses ultimately led to the correction of the configuration, reallocating the entire northern part to the inverted configuration (**2*r*** in Figure 6).

Since the configurations **2*p*** and its *11-epi***-2*p*** were synthesized in larger scale in a local laboratory and characterized using NMR, this excellent material was made available to test the MDOC methodology on a challenging, larger-sized and flexible molecule.

The current structural characterization of mandelalide A isomers relies on carefully evaluated RDC values, NOE distances and scalar ^3^*J* couplings. In the case of structure **2*p* (***11-epi*-**2*p*),** a full unambiguous ^1^H and ^13^C assignment was obtained (including the prochiral ^1^H assignment 7 CH_2_), and complemented with 45 (48) CH RDC values, 38 (38) ^3^*J*_HH_ couplings and 129 (106) NOE distances (see Supplementary Information). Any attempt to obtain alignment tensor evaluation using the SVD approach on calculated structural models failed (data not shown). The NMR-based structural information, however, was used as constraints in a 41-ns MDOC simulations (see Supplementary Information).

**Figure 7:**
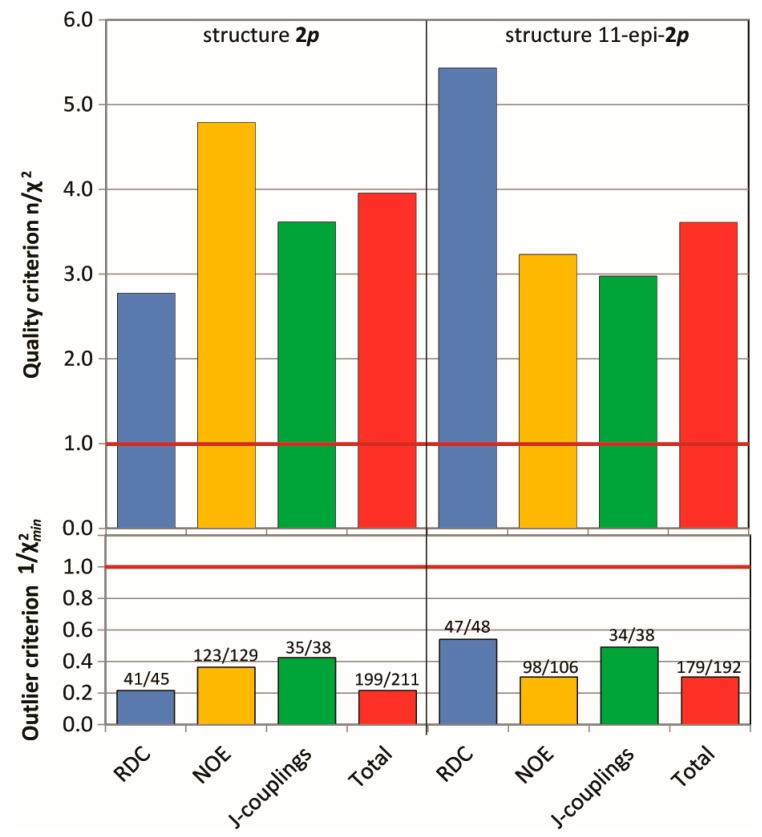
Criterion n/χ^2^(top), outlier criterion1/χ^2^_min_ (bottom) and fidelity ℱ (labels) of the MDOC simulations of the configurations **2*p*** and *11-epi***-2*p.*** Data is presented for each type of NMR parameters.

The MDOC simulations (see SI for details) of the madelalide A isomers are more challenging than the 1,4-diketone simulations because conformational changes of the ring system have larger energy barriers. The rotational degrees of freedom of the side chains add up to the complexity of the system. As indicated by the n/χ^2^ quality criteria (Figure 7), values back-calculated from the MDOC trajectory are on average well within the experimental error bounds (n/χ^2^ > 1). As for the MDOC simulation of the 1,4-diketone **1**, inspection of the individual data reveals however the occasional parameter were not completely fulfilled (see labels representing the fidelity ℱ in Figure 7). The outliers represent generally less than 10% of the overall NMR constraints. Furthermore, based on the magnitude of the outlier criterion, $\chi_{min}^{-2}$, the worst of these outliers deviate by less than one error margin. Whereas the error margins for the RDC values were determined individually, those for the NOE distances were estimated to 0.5Å. In the case of ^3^*J* couplings, the errors were assessed to be about 1.0 Hz taking into account also the possible uncertainties of the equation of Haasnoot et al. [4]. The RMS deviation of the NOE distances was lower than 0.3 Å and that of the ^3^*J* couplings below 0.6 Hz.

The final mean data values of the MDOC simulations are calculated from 2000 coordinate, skipping the first nanosecond of the 41-ns trajectories. These snapshots contain the calculated NMR data (RDC, ^3^*J* couplings and NOE distances) as averaged according to the memory function as given in Equation (3). In other words, the snapshots contain average information from every time step of the trajectory. Considering the n/χ^2^ criteria (Equation (7)) or the calculated data (see Supplementary Information), it can be stated that the MDOC results are mostly within the estimated error bounds and no severe outliers are observed. Since the NMR data are both time- and ensemble-averaged values, it can also be stated that the MDOC simulation for a single molecule behaves to a good approximation in an *ergodic* manner. In this sense, the 2000 coordinate and data snapshots represent the final result of the simulations. Since this larger flexible molecules may undergo many conformational changes, the population of these states may be of high interest and therefore advanced methods need to be employed to analyze these populations.

#### 2.2.2. Torsion Angle Distributions

**Figure 8:**
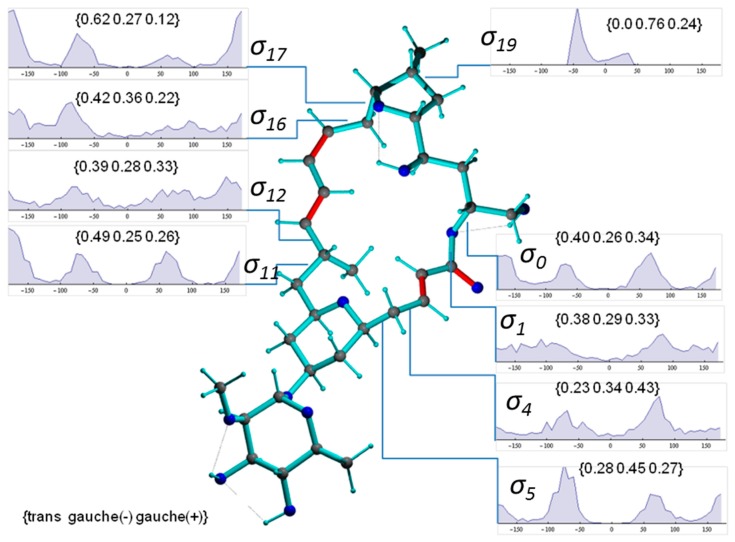
Conformer of mandelalide A configuration ***2p*** including some torsion distributions around single bonds (π-bonds are displayed in red). Dihedral angles are denoted with $\sigma_{n}$, where n refers to the 3rd atom of the dihedral angle definition (e.g., $\sigma_{1}\equiv$ C23-O-C1-C2). The torsion distributions involve only carbons or oxygens of the large ring system. Inset labels indicate {*trans, gauche-*, *gauche+*} ratios.

**Figure 9:**
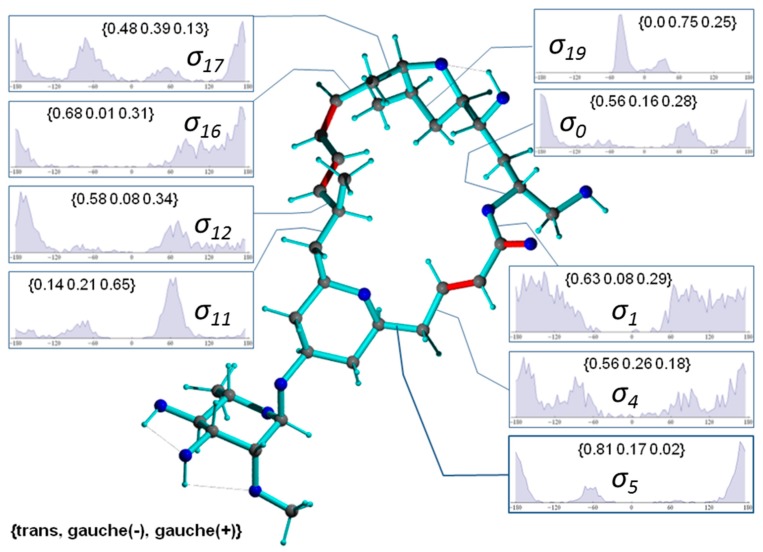
Conformer of mandelalide A configuration *11-epi-***2*p*** including selected occupation numbers of torsional states around single bonds (π-bonds are displayed in red). Dihedral angles are denoted with $\sigma_{n}$, where *n* refers to the 3rd atom of the dihedral angle definition (e.g., $\sigma_{1}\equiv$ C23-O-C1-C2). The torsion distributions involve only carbons or oxygens of the ring system. Inset labels indicate {*trans, gauche-*, *gauche+*} ratios.

Figure 8 presents a representative conformer of the configuration **2*p*** with torsion angles commonly populated within the 1000 MDOC coordinate snapshots (the Supplementary Information contains a collection of 10 typical conformers). This conformer also exhibits the lowest total force field energy among all snapshots. Also displayed Figure 8 are the torsion distributions of some σ-bonds that display highest variability throughout the trajectory. As can be seen, many bonds sample a wide range of dihedral angles even within a closed 24-membered macrolide ring, indicating large amplitude motions and complex conformation exchange. Noteworthily, the oxane 6-ring (-C5-C6-C7-C8-C9-O-) did not change from its chair conformation throughout the simulation although the oxolane 5-ring displayed two twist states as demonstrated by σ_19_ with a slight preference of *g(-)* conformation (Figure 9, top-right inset).

The representation of the fluctuation of the tethered rhamnose ring proved to be a special case. From its sparse and weak NOEs to the lactone ring, it can be expected to undergo conformational changes with low correlation to the large macrolide ring. Indeed, the MDOC simulation resulted in a flexible oxygen bridge with several large scale modes of motions in order to average the parameters down to reliable mean values in the rhamnose system. The ether bond to the oxane ring (C7-O) showed nearly no preference for a torsion state while the ether bond to the rhamnose ring (O-C51) favored the *trans*-position to the carbon with the O-CH_3_ group (Figure 8). The dominant rhamnose ring conformation was characterized by the O-CH_3_ in axial position of the ring and the two OH groups and the CH_3_ group in equatorial positions. This conformation is strongly supported by the ^3^*J* couplings of the protons of the rhamnose ring and rendered by the MDOC simulation, although nearly 10% rhamnose ring inversions were also generated.

In Figure 9, a typical conformer of *11-epi*-**2*p*** selected from a set of 1000 snapshots of an 40-ns MDOC simulation is presented, along with the distribution of key dihedral angles. It is instructive to compare the significantly different rotameric distribution of torsion angles with the values given for configuration **2*p*** in Figure 8. Not surprisingly, the dynamics present in the smaller rings are almost identical in both diastereomers (e.g., $\sigma_{19}$). In the larger macrolide ring, however, not only are the torsion angle distributions in the direct vicinity of the epimerization site affected ($\sigma_{11}$ and $\sigma_{12}$) but also those at moderately and very distant sites (e.g., $\sigma_{0}$, $\sigma_{4}$, $\sigma_{5}$ and $\sigma_{17})$. This is indicative of long-range trans-annular steric interactions of the methyl group at position C11. However, a clearer conformational picture can only emerge from the examination of the entire structural ensemble generated by the MDOC simulation.

#### 2.2.3. Principal Component Analysis

The representative models shown in Figure 8 were selected because their dihedral angles match a majority of the most highly populated rotameric states of the MDOC ensemble and they represent states of low force field energy. But just as for **1**, it would be desirable to rationalize the dynamics of the mandelalide A isomers **2** in terms of population of major conformers with representative torsion angle combinations. Instead of only three rotameric states as in **1**, there are nine “rotatable” σ-bond (Figure 6) each with roughly three main rotamers, leading to nearly 20,000 possible combinations of angles. Though the MDOC simulations indicate that very broad distribution of angles is sampled by all of these nine key bonds, all combinations of angles are, of course, not accessible in reality. The challenge here lies in the complexity of the conformational space, and in finding the right tools to describe its essence.

A general and elegant method to evaluate the present MD data is the principal component analysis (PCA) on the basis of dihedral angles as developed by Altis et al. [38]. In its substance, PCA distills and ranks the geometrical parameters (e.g., atom coordinates) contributing most to a fluctuating system (e.g., MD trajectory) by identifying the orthogonal eigenvectors from the diagonalised co-variance matrix. In contrast to using methods based on Cartesian coordinates, PCA based on dihedral angles (“dPCA”) provides a correct separation of internal and overall dynamics and places more emphasis on large structural rearrangements and less on bond length fluctuations. Since it deals with angular variables, dPCA has the added intricacy of accounting for circular statistics by transforming the angles to a complex coordinate space. A slightly adapted variant of the complex dPCA metod was used here to identify conformers with low energy.

**Figure 10:**
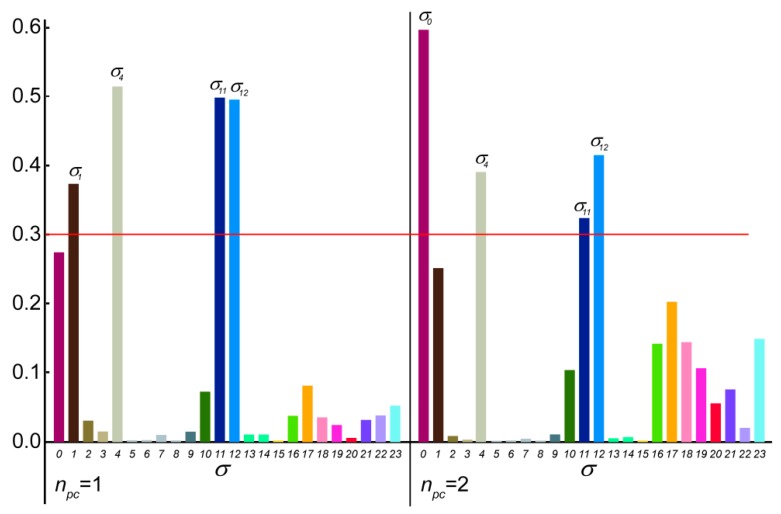
Contribution of the selected 24 σ dihedral angles along the mandelalide A ring main-chain to the first 2 principal components with highest ranked eigenvalues. Dominant contributions (>0.3) are indicated with the shorthand $\sigma_{n}$, where n refers to the 3rd atom of the dihedral angle definition (e.g., $\sigma_{12}\equiv$ C10-C11-C12-C13, also: $\sigma_{0}\equiv$ C22-C23-O-C1).

**Figure 11:**
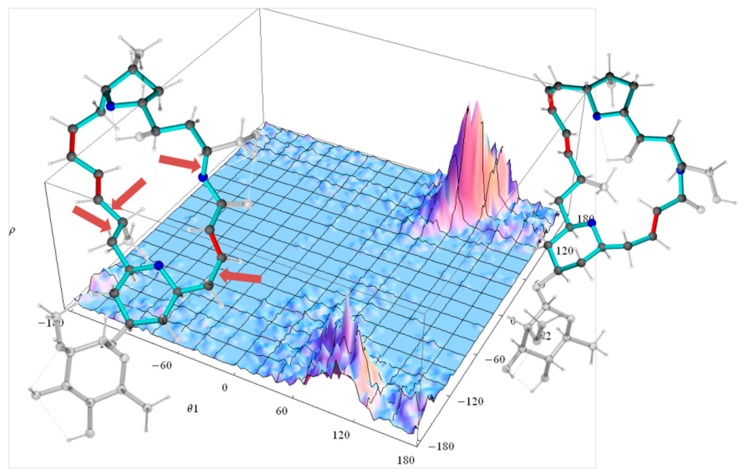
Dihedral landscape of the first two principal components of the mandelalide A isomer **2*p*** with two typical molecules according their contribution to the principal components (Figure 10). The left conformation is selected according to the lower θ_2_ maximum of the dihedral distribution and the right molecule according to the upper θ_2_ maximum. The side chains of the large ring system are displayed transparent. The arrows indicate the largest contributions to the first two principal components: both bonds to methyl-group-bearing C11 ($\sigma_{11}$ and $\sigma_{12}$), the bond to the ring oxygen O1 ($\sigma_{0}$) and the bond the methylene C4 ($\sigma_{4}$).

In the case of the **2*p*** isomer of mandelalide A, all 24 of the macrolide dihedral angles within the large ring were selected as the basis for the dPCA. Results are shown in Figure 10, where the relative contribution of each of the 24 dihedral angles to the angular variance is displayed for the first two principal components. Further components (n_pc_ > 2) were already less significant. Out of the 24 bonds, five contribute very significantly to the overall conformational fluctuations within the MDOC ensemble, namely $\sigma_{0}$, $\sigma_{1}$, $\sigma_{4}$, $\sigma_{11}$ and $\sigma_{12}$, which according to Figure 8 are involved in fluctuations of large amplitude.The product of each eigenvector *n* with the dihedral angle matrix deliver a single angular value *θ_n_* for a given MD snapshot. In Figure 11, the (*θ*_1_, *θ*_2_) distribution for the first two principal components obtained for the dPCA of mandelalide A **2*p*** is presented from all 8000 data points each corresponding to an MDOC snapshot taken every 5 ps. The resulting distribution identifies three sub-regions at approximately (*θ*_1_, *θ*_2_) = (+100, +130) [45%], (+100, −150) [40%] and (170, −150) [~5%]. In principle, these describe the subsets of large amplitude fluctuations contributing the most to the overall dynamics within the large ring system. Though the *θ*_1_ and *θ*_2_ angles bear no direct geometrical representation in the macrolide, they correspond to a linear combination of the angles highlighted in Figure 10. The major conformational exchanges between the three sub-regions thus involve correlated variations of these angles. Structural representation of typical conformers for the two principal subregions are also shown in Figure 11.

The question arises whether sparsely populated conformers are accurately represented in this simulation ensemble. The requirement that the ensemble meet the stringent rule of satisfying a very large number of NMR parameters within their error margins would certainly elicit emergence of low-populated states. So far, conformation subsets with populations ≥5% have proved to be essential for achieving the n/χ^2^ criterion. Further investigations will however be required to establish the relationship between the abundance of short- and long-range constraints and the representation of low-energy conformations. There are several experimental and theoretical investigations that discuss precisely measured NMR parameters, especially NOE distances, as sensitive indicators on sparsely populated conformers to elucidate conformer equilibria [39,40].

An analogous analysis was also conducted for the isomer 11-epi**-2*p*** (Supplementary Information). The corresponding dominantly contributing torsion angles are different to those of **2*p***: whereas $\sigma_{1}$, $\sigma_{4}$, $\sigma_{11}$ and $\sigma_{12}$ have similarly important co-variation, $\sigma_{0}$ and $\sigma_{17}$ are relatively more involved. Since the eigenvectors have different angular linear contributions, the dihedral landscapes for (*θ*_1_, *θ*_2_) cannot be directly compared; still, one can notice that the 11-epi**-2*p*** conformational equilibrium is constituted of two major sub-regions ((*θ*_1_, *θ*_2_) = (120°,160°) and (175°,120°)) with well-defined distributions. The results here show that transannular steric influence reaching the opposite part of the 24-membered ring seems to favor an overall more open ring conformation for 11-epi**-2*p***.

Intuitively, one might expect only minor differences in the conformational distribution of **2*p*** and its isomer 11-epi**-2*p***, whose configurations only differ in the chirality of a single methyl group, and these differences would be expected to be manifest mainly in the NMR data of nuclei in the direct vicinity of the C11-CH_3_ group, but based on Figure 8 and Figure 9 as well as in the PCA results, this is not the case - the disparities hint to divergent conformer distributions. Though it is perhaps unexpected that a single methyl group epimerization leads to considerable change in the conformer distribution of the entire ring system, the significantly different NMR spectra and datasets corroborates with this finding. By contrast, the oxolane 5-ring, the oxane 6-ring and the rhamnose ring displayed no significant difference comparing **2*p*** and *11-epi*-**2*p*.**

The resulting structural ensembles are the product of MD simulations driven by a rich set of NMR data, including NOEs, J-couplings and RDCs. Since these interactions act in reality on different time scales, no attempts were made here to extract kinetic information from these results. However, since MD simulations generally include energetic and entropic influences, these MDOC results propose that it is possible to extract a detailed picture of the conformer distribution and equilibrium in solution. The high consistency of these dynamic results with their corresponding data leads to the interrogation whether MDOC could be confidently used towards determination of configuration in flexible molecules such as natural products.

### 2.3. Configuration Determination with MDOC

It would be interesting to evaluate the diastereomeric discrimination potential of MDOC for flexible molecules. MDOC was already introduced for this task in relatively rigid and slightly flexible molecules [32,33], but in the present case, it is expected that orientation pseudo-forces may arrive more easily at false positives (e.g., structures with quality criterion >1) especially if the molecule’s topology allows for large geometrical fluctuations due to increased number of degrees of freedom. On the other hand, the pseudo-forces from short-range NOEs and *J-*couplings may play an important role in hindering unrealistic ensembles. The test molecules at hand are ideally suited for this evaluation, since two diastereomers are available and can be used reciprocally for cross-validation.

#### 2.3.1. Discriminating 1,4-Diketone 1-SS vs. 1-SR

**Figure 12:**
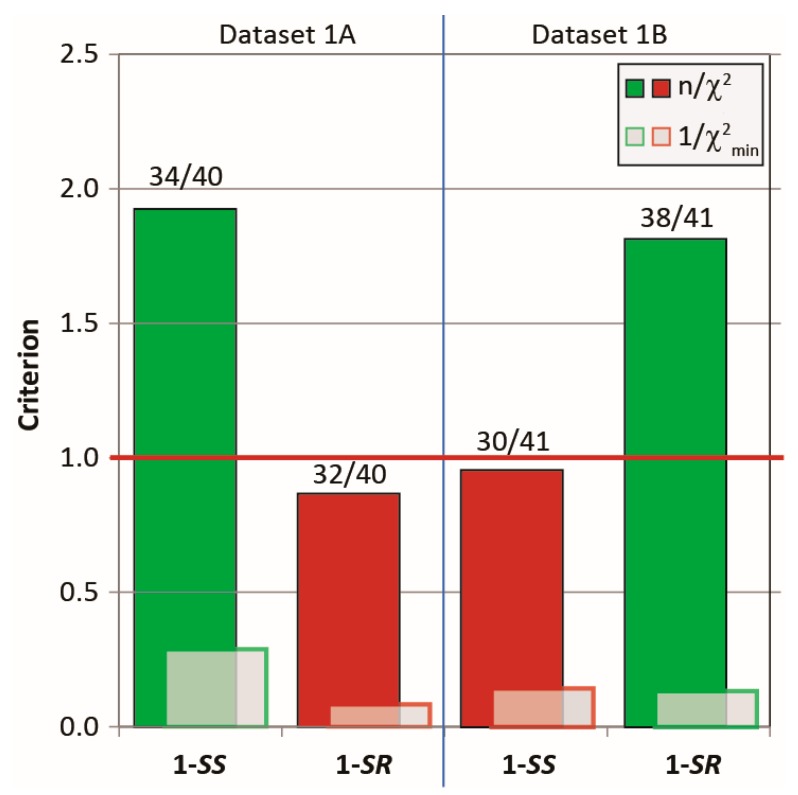
The MDOC outcome based on datasets 1A and 1B against the correct (green) and incorrect (red) diketone **1** configuration. Displayed are the three criteria (quality criterion *n/χ*^2^, outlier criterion (*1/χ*^2^_*min*_) and data validity ℱ (labels)). For the breakdown of these parameters based on data type, see Figure S2 in the Supporting Information).

As seen in Figure 12, all NMR parameters extracted from the MDOC trajectory run with dataset 1A scored better according on three criteria (*n/χ*^2^, *1/χ*^2^_*min*_ and ℱ) with the corresponding **1-*SS*** configuration and, conversely, those with dataset 1B scored better (or equally well (*1/χ*^2^_*min*_)) with the ***1-SR*** form. More importantly, the wrong configuration does not pass the quality criterion in both cases. The individual parameter types taken individually (1-bond RDCs, long-range RDCs, NOE distances and ^3^*J*-couplings) also follow the trend that the criteria are consistently better for the correct configuration (Table S6 and Figure S12 in Supporting Information). The only exception regards the ^3^*J*-couplings of dataset 1A for which the three values are satisfied almost equally for both diastereomers. Note that the three ^3^*J-*couplings entered as constraints into the simulation influence the ω dihedral angle, and that these values are more characteristic of the conformational exchange than of the configuration. By contrast, the NOE distances seems to be highly sensitive to the configuration.

#### 2.3.2. Discriminating between 4 Stereoisomers of Mandelalide A 2

**Figure 13:**
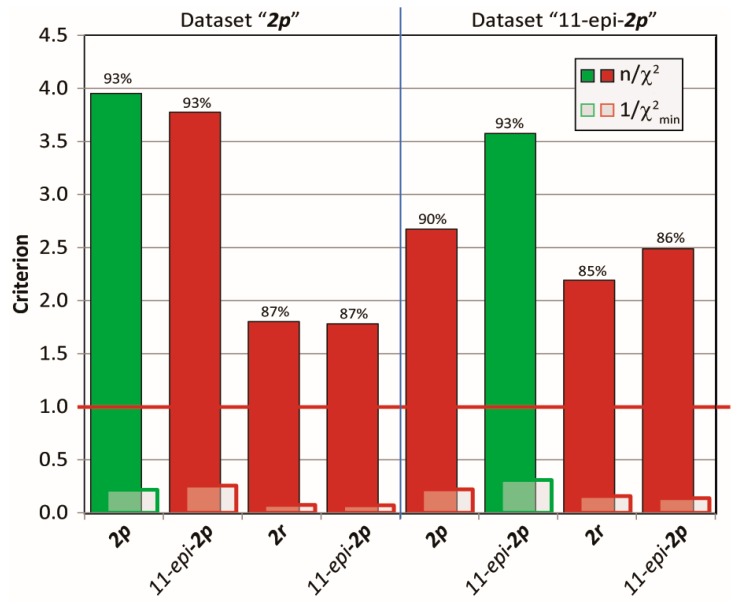
Quality criteria (*n/χ*^2^, *1/χ*^2^_*min*_ and ℱ) for MDOC simulations of four configurations with two different NMR data sets. The configuration that belongs to the data is indicated in green.

Next, cross-validations of the datasets for the mandelalide A isomers **2** were tested in MDOC simulation against the incorrect configurations to address the diastereomeric discriminating ability of MDOC in more complex flexible molecules with various chiral centers. To this end, MDOC simulations with identical parameters (see SI) were applied to four configurations **2*p***, *11-epi*-**2*p***, **2*r*** and *11-epi*-**2*r***, and the accuracy of the simulated NMR parameters were examined based again on the three aforementioned criteria. The results are displayed in Figure 13. Although the quality criterion is met (>1) for all configurations, the isomers **2*r*** and *11-epi*-**2*r*** with their inverted northern part show a substantial drop in the quality of fit relative to the correct ones (**2*p*** and *11-epi***-2*p***, respectively). However, the inversion of the methyl group at position 11 does not lead to a large deterioration of the quality - especially with NMR dataset **2*p*** (4.64 to 4.20). Closer inspection however showed that the small drop in quality is caused mainly by a number of long-range NOE distances, whereas the simulated RDCs and *J*-coupling could not readily distinguish either configurations. In contrast, a more distinct drop in quality is observed for the *11-epi*-**2*p*** dataset applied to configuration **2*p***.

From these results, it can be stated that in complex flexible molecules with various rotatable bonds and chiral centers, the MDOC-based discrimination of configurations on the basis of one-bond RDCs alone will be impracticable in a majority of cases; however, with the inclusion of additional high quality parameters such as NOE distances, some level of success should be expected. The example of mandelalide A already shows potential in this regard. With the refinement of the method and with the use of advanced statistical tools, it is conceivable that MDOC could provide a robust and general approach to assist in the difficult task of determining stereochemistry in larger natural products.

## 3. Conclusions

In this report, the MDOC method is proposed as a tensor-free approach for the conformational analysis of flexible molecules based on RDCs measured at high resolution in orienting media. One advantage of the MDOC method is that a vast range of interconversions are efficiently driven by the action of orientational pseudo-forces derived from tensorial properties, so that large compatible ensemble of conformers are generated. Since the expression of each orientational pseudo-energy possesses multiple minima, it is expected that the simulated ensemble may include non-native conformations, leading to inaccurate population equilibria. It is however another advantage of the MDOC method that it can be combined with traditional short-range scalar constraints like NOE distances or ^3^*J* couplings, which are easily measured in solution and which should limit the generation of unrealistic rotamers. The entire NMR dataset can be brought together in a single simulation to generate a consistent picture regarding the structure of molecules in solution and their dynamic equilibrium. For the **2*p*** isomer of mandelalide A, for instance, it can at least be stated that the resulting ensemble are in accordance with no less than 212 NMR constraints. Further investigations will show to what extent this method can be generalized.

The cross-validation applied on different configurations of the same molecules offered the opportunity to show that MDOC has the ability to discriminate between diastereomers although the level of contrast depends on the quality of the NMR data and on how strongly the stereogenic centers interact with the rest of the structure and induce overall changes in the conformer distribution. In the case of the relatively small diketone pair, **1-*SS*** and **1-*SR***, differing at a single chiral position, the MDOC results fit the incorrect diastereomer quite well by conventional standard testing (e.g., RMS or Q), but according to the n/χ^2^ and 1/χ^2^ criteria, the outcome is much clearer. In the case of the much larger mandelalide A, configuration **2*p*** can readily be distinguished from **2*r*** but in the case of the epimers **2*p*** and *11-epi*-**2*p*** the difference is barely significant. Nevertheless, all 4 configurations pass the n/χ^2^ test and have similar in 1/χ^2^ values. This is not surprising since a majority of the short-range constraints (NOEs and *J*) are only weakly affected by the chiral difference at distant positions. It would thus be desirable to take these aspects into consideration and develop more advanced statistical tools in order to confidently use MDOC for configurational evaluations in flexible molecules.

As a consequence, MDOC may become a method of choice for the NMR investigation of flexible molecules where the structure and distribution of conformers are of interest. To solve these questions can be of high interest in all areas of chemistry where conformational selection is of interest such as in the study of interactions of biological active molecules with their targets or in the mechanistic understanding of stereoselective catalytic reactions.

## 4. Materials and Methods

### 4.1. NMR

#### 4.1.1. Materials

The monomers, methyl methacrylate (MMA, 99%, Sigma-Aldrich, St. Louis, MO, USA) and ethylene glycoldimethacrylate (EGDMA, 98%, Sigma-Aldrich) were purified prior to the experiments by passing the neat liquids through a short column filled with basic alumina in order to remove the polymerization inhibitor. The radical initiator 2,2’-azobis(2,4-dimethyl-4-methoxyvaleronitrile (V-70) was purchased from Wako (Neuss, Germany), and acetone-d_6_ and chloroform-d_1_ (99.9% of D atoms) were purchased from Cambridge Isotope Laboratories (Tewksbury, MA, USA). Twenty five mg of a single diastereomer of the 1,4 diketone was kindly donated by the List group, and was a product of an asymmetric cross coupling catalysis reaction [34]. About 2 mg of “pseudo”-mandelalide and *11-epi*-“pseudo”-mandelalide (recognized as isomers of mandelalide A as a result of its synthesis, following the inversion of the northern part) were obtained from the Fürstner group as the end product of a multistep synthesis by Willwacher et al. [41]

#### 4.1.2. Sample Alignment

Alignment of the diketone and of the mandelalide was achieved using reversible compression of polymethylmetacrylate (PMMA) gels prepared as described by Gayathri et al. [42]. PMMA gel sticks of about 2 mm in diameter and 25 mm long and crosslink density of 0.3 mol% were pre-swollen in chloroform-d_1_, inserted into a 5-mm NMR tube. Residual monomers were washed out of the polymer stick gel by applying several compession-and-release cycles in the deuterated solvent (chloroform-d_1_) using a home-made Teflon plunger. This was repeated until no monomer could be observed in the 1D ^1^H NMR spectrum. The gels exhibited quadrupolar splitting (Δν_Q_) of the solvent signal of form 40 to 47 Hz when maximally compressed, and no quadrupolar splitting (Δν_Q_ = 0) when fully relaxed as assessed by 1D 2H NMR. The homogeneity of the alignment was also assessed using ^2^H-mapping approaches [43]. The dissolved analytes (diketones or mandelalides) were dispersed into the gel using a pumping actions as described above. Samples could be measured immediately.

#### 4.1.3. NMR Experiments

NMR experiments for the 1,4-diketone samples were collected on an Avance III NMR instrument (Bruker BioSpin GmbH, Ettlingen, Germany) operating at 499.87 MHz for ^1^H, and 125.69 MHz for ^13^C and equipped with a broad band observe (incl.^19^F) probehead (BBFO) with Z gradients. NMR experiments for the mandelalide samples were collected on a Bruker Avance NMR instrument operating at 600.22 MHz for ^1^H, and 150.93 MHz for ^13^C and equipped with cryogenically cooled triple channel (^1^H/^13^C/^15^N) inverse probehead (CPTCI) with Z gradients. Complete ^1^H and ^13^C assignments, including stereotopic ^1^H in methylene groups, were obtained at 25 °C from standard 1D experiments as well as 2D correlation experiments. The correlation experiments included ^1^H,^1^H-DQF-COSY, ^1^H,^1^H-NOESY, ^1^H,^13^C-HSQC, ^1^H,^13^C-HMBC. The ^1^H and ^13^C spectra were referenced by setting the residual solvent signal at their known chemical shift relative to TMS. Homonuclear ^1^H scalar coupling constants were measured directly on 1D ^1^H NMR spectra in resolved cases and using selective 1D NOE experiments in cases where ^1^H signals were overlapped. Cross-relaxation NOEs were evaluated from a ^1^H,^1^H NOESY experiments measured with 2048 × 1024 points in the acquisition matrix, 6(8) scans per increment and 1(0.7) s mixing time and 3(6) s relaxation delay for the diketone (mandelalide) sample. CLIP-HSQC experiments [44] were recorded to measure the CH splittings corresponding to scalar couplings (^1^*J*_CH_) or to scalar plus residual dipolar couplings (^1^*T*_CH_ = ^1^*J*_CH_ + ^1^*D*_CH_) in the isotropic and anisotropic samples. This experiment was typically parametrized with 16k x 512 points in the acquisition matrix, 8 scans per increment and 3-s relaxation delay. A homo-decoupled CLIP-RESET-HSQC [45] was also measured in the case of the mandelalide sample as a 3D matrix of 512 × 512 × 12 (chunks) points, with chunk duration of 16.255 ms, 8 scans per increment and relaxation delay of 1 s. All raw, processed and analysed data are made available as NMReDATA records [46].

#### 4.1.4. Conformational Analysis

The initial input structures for the two possible diastereomers of diketone 1 (**1-*SS*** and **1-*SR***) in three different rotamers (*trans*, *gauche+* and *gauche-*) were generated with the hybrid density function B3LYP, cc-pVTZ as a basis set in Gaussian-09. For the conformation analysis of these structures against the measured RDCs based on the classical alignment method using SVD, the program Mspin (Mestrelabs, Santiago de Compostela, Spain) was employed. All inputs are given in the Supporting Informations.

### 4.2. MDOC Simulations

#### 4.2.1. Theory: RDC-Based Orientation Constraints

The general methodology of MDOC has already been outlined [30,31] and described more specifically for RDCs [32,33]. Since new methodological features are introduced in this paper some essential points of the method are discussed in this section.

Prerequisite of MDOC is a molecular mechanics force field that is flexible enough to calculate the relative energies of most organic molecules and provide structures that compare well to diffraction experiments or more elaborated ab initio or DFT calculations. The COSMOS-NMR [47,48] force field that was used in this case has one distinctive advantage over most other force fields - it uses partial atomic charges from a quantum chemical method [48,49,50] (Bond Polarization Theory - BPT) to calculate the electrostatic energy. Since these charges can be recalculated in the course of an MD simulation, all mutual polarization can be included into the electrostatic energy. This particular performance of the force field is an issue especially if orientational constraints are applied, since their ambiguities/degeneracies have a tendency to drive the MD towards unrealistic structures. By contrast, MD with pseudo-forces based only on scalar constraints are more self-contained.

The pseudo-energy in the case of tensorial properties like dipolar couplings has a special form:

(1) $$E_{pseudo}=\frac{K}{2}\sum_{\alpha \beta }\sum_{i}{\left(D_{\alpha \beta }^{the,i}-D_{\alpha \beta }^{\exp,i}\right)}^{2}$$

The first double sum (denoted with αβ - the indices α and β are used to denote the coordinates x, y and z) in Equation (1) runs over all nine components of the dipolar tensor **D** and all tensor components are regarded as constraints. Other pseudo-energies for scalar constraints like NOE distances or scalar *J* couplings have only the second summation over the experimental values (denoted with *i*). The constant *k* is used to convert the expression into kJ/mol and to adjust the strength of the pseudo-forces. Measured RDC values represent the z-components of diagonal traceless tensors whereas the off-diagonal elements are averaged to zero by the rapid reorientation of the molecule around the director. The measured tensors **D***^exp^* are given in the laboratory coordinate system, oriented with its z-axis relative to the direction of the external magnetic field. The calculation of dipolar coupling tensor **D***^theo^* can easily be performed in the coordinate system that is oriented parallel to the vector that connects the two coupled nuclei. In this case the dipolar tensor is diagonal and its principal values can be calculated from the product of the gyromagnetic ratios of the two nuclei divided by the third power of their distance (also see Supplementary Information). To calculate the differences between the calculated and experimental tensor components, the tensor **D***^theo^* has to be transformed from its principal axis system to the laboratory coordinate system using proper transformation matrices **T***^i^*:

(2) $$D_{\alpha \beta }^{theo_{i}}=\sum_{\alpha^{\prime }\beta^{\prime }}^{3}T_{\alpha \alpha^{\prime }}^{i}T_{\beta \beta^{\prime }}^{i}D_{\alpha^{\prime }\beta^{\prime }}^{CH}$$

In Equation (2) the dipolar coupling of ^1^H and a ^13^C nucleus in a C-H bond is chosen as example. This coupling is nearly the same for all C-H bonds since the bond distance is nearly constant (in the present simulations, different values only for C(*sp*^3^)-H and C(*sp*^2^)-H couplings are used—see Supplementary Information). The double sum in Equation (2) runs over all components of the transformation matrices **T***^i^* being mostly different for all sites *i* because of the different orientation of the bonds with respect to the laboratory system.

The dipolar tensor components obtained from Equation (2) cannot be directly compared with experimental tensor components since they vary within several tens of kHz whereas the experimental RDC have mean values that span only several Hz. Therefore a theoretical mean value is calculated during the time of the MD simulation. For this mean value calculation, a special form is chosen that implies a memory time *τ*:

(3) $$\begin{array}{l} \langle D\left(t\right)\rangle =\frac{1}{N\left(t\right)}\int_{t^{\prime }=t_{0}}^{t}e^{\frac{-\left(t-t^{\prime }\right)}{\tau }}D\left(t^{\prime }\right)\;\;\;\;dt^{\prime }\\ N\left(t\right)=\int_{t^{\prime }=t_{0}}^{t}e^{\frac{-\left(t-t^{\prime }\right)}{\tau }}\;\;\;\;dt^{\prime } \end{array}$$

The exponential decaying memory function was first introduced by Torda et al. [51] and leads to a time-dependent property on the time scale of the decay or memory time τ. In the context of MDOC, this time τ can be considered as life time of an orientation of a molecule or molecular part. Since the pseudo-forces depend on this lifetime, τ should be considered as the time scale for orientational and conformational changes. After 5τ, less than 1% of the original orientation as expressed by the instantaneous value of **D** is left and the pseudo-forces change on the same time scale τ because of the mismatch of calculated and experimental data. In this respect, MDOC can be regarded as an accelerated MD simulation and orientational averages can be reached in moderate simulation times. A value of τ of 200 picoseconds was generally used whereas the simulations were performed over durations one or two order of magnitudes longer than τ − i.e., over several nanoseconds.

Since in MD simulations the integration of the equations of motion is performed in time steps, the integration in Equation (3) can be transformed into a summation. To speed up the calculation a recursion formula for this integral was developed (see Supplementary Information). The formalism was used for all types of tensorial and scalar constraints as for instance for NOE distances and scalar couplings. In MD simulations, the energies and pseudo-energies (Equation (1)) are not directly used but the forces that are calculated from these energies as first derivatives with respect to the coordinates of the nuclei *A_i_* and *B_i_*:

(4) $$F_{x\left(A_{i}B_{i}\right)}^{i}=k\sum_{\alpha \beta }^{3}\left(D_{\alpha \beta }^{theo_{i}}-D_{\alpha \beta }^{\exp_{i}}\right)\frac{\partial }{\partial x_{\left(A_{i}B_{i}\right)}}D_{\alpha \beta }^{theo_{i}}$$

From Equation (4), the magnitude of the forces on the coupling atoms grows linearly with the difference between experimental and calculated RDC. This dependence occurs for harmonic potentials like Equation (1) but for other molecular potentials like those of bonds harmonic behavior occurs only for deviations not far from the minimum. For large deviations as in the case of pseudo-forces harmonic potentials lead to very high pseudo-forces and consequently to unrealistic deformations of the structure. Therefore, the difference $\left(D_{\alpha \beta }^{theo_{i}}-D_{\alpha \beta }^{\exp_{i}}\right)$ n Equation (4) is replaced with a scaling factor f(ΔD) with a different dependence:

(5) $$f\left(\Delta D\right)=\frac{s-s^{-1}}{s+s^{-1}}\;and\;s=e^{\frac{\left(D^{theo}-D^{\exp}\right)}{\Delta D}}$$

Equation (5) represents a hyperbolic tangent dependence that becomes constant if the difference between calculated and experimental RDC gets much larger than a width parameter *ΔD*. This width parameter can be evaluated as the experimental error of the RDC.

Inserting Equation (2) into Equation (4) reveals that orientational pseudo-forces imply derivatives of the transformation matrices **T***^i^* in Equation (2) with respect to the coordinates coupling nuclei. The transformation matrices can be obtained from sets of orthogonal unit vectors that span the local bond (or interconnection) coordinates systems and therefore the derivatives can be obtained from these unit vectors (see Sternberg et al. [31]). In contrast to 1-bond RDCs(^1^*D*), long-range RDCs (*^n^D*) can also be used as distance constraints in analogy to NOE distances, providing the mean distribution of orientations of nuclear connection vectors (or alignment tensor) is known. However, in this investigation, only the orientational derivatives of the RDC pseudo-energies are used.

It should be remarked that the magnitude of the pseudo-forces is controlled by the differences between experimental values and mean values calculated according to Equation (3) but the direction of the orientational pseudo-forces is calculated from derivatives of the actual transformation matrices (see Equation (2)). In this way a mismatch in orientation acts immediately. The problem is that at start of the MDOC, there are no mean values and the strong pseudo-forces would completely distort the molecule. For this reason, the pseudo-forces are introduced in exponential fashion using $1-e^{-t/\rho }$ with *ρ* the time constant for the rise of the pseudo-forces. This value was set in the simulation to the same value as the memory time τ (see Equation (3)).

In former investigations, the orientation and motions of molecules in lipid membranes were studied. These liquid crystalline systems display a high degree of order that is expressed using the so called Saupe order parameter *S* (for review see Limmer [52]). The molecules in these investigations reached order parameters up to S ~ 0.8. In the present investigation weakly oriented media such as stretched gels were used allowing order parameters in the range of 10^−2^ to 10^−3^. Therefore an order parameter *S_am_* is introduced into the calculations that accounts for the reduction of the dipolar splitting caused by the alignment medium (in analogy to the order parameter *S_bilayer_* introduced by Marsan et al. [53]). *S_am_* is multiplied to the calculated dipolar couplings to prevents too high pseudo-forces by shifting the dipolar splittings from the kHz range to several Hertz. *S_am_* should be chosen large enough that its product with the maximum splitting is in any case much larger than any observed splitting. If, for example, the static C-H splitting of 47.96 kHz is multiplied with *S_am_* (0.004, see Table S10 in Supplementary Information) a maximum attainable CH splitting of 191.8 Hz is obtained, that is much larger than any observed splitting.

#### 4.2.2. Theory: Scalar Constraints

In addition to the RDC, NOE distances and ^3^*J_HH_* indirect scalar couplings are used as constraints. For the calculation of the time mean values in both cases the average with an exponential memory as given in Equation (3) was used. The pseudo-forces are augmented with hyperbolic tangent weight function as given in Equation (5). For the calculation of the distances the following average is applied [54]:

(6) $$\bar{r}(t)={\langle r^{-6}\rangle }^{-1/6}$$

(the mean value calculation is indicated using the symbol < >). Equation (6) is valid for rigid molecules that undergo isotropic reorientations [49]

In MDOC simulations, it is possible to directly use NOE distances of protons of CH_3_ groups as constraints. If for the CH_3_ protons also one bond RDC are used as constraints these groups rotate fast in the MDOC simulations and therefore the same distance constraint can be applied to all three protons of a CH_3_ group.

For the calculation of the ^3^*J_HH_* couplings the equation according to Haasnoot et al. [4] was used. In this Karplus-like equation a correction term is added that accounts for the influence of the electronegativity of neighbor substituents on the coupling protons. Using this equation an RMS (root mean square) deviation between calculation and experiment in the range of ca. 0.6 Hz should be possible.

#### 4.2.3. Evaluation of Quality

In many investigations, the RMS deviation between calculated and measured values is used to estimate the performance of theoretical or computational methods. The disadvantage of the RMS criterion is that (i) the valuable information about the error of the measurement is disregarded and (ii) since the RMS value has the unit of the measurement different data sets as for instance distances and frequencies cannot be compared properly. Therefore the *n/χ*^2^ criterion is used, as introduced by Intelmann et al. [55]:

(7) $$\chi^{2}={\sum_{i}^{n}\left(\frac{P_{i}^{theo}-P_{i}^{\exp}}{Error_{i}^{\exp}}\right)}^{2}$$

The quality of a computation is expressed as *n/χ*^2^ (with *n* the number of measured values of the property *P*) giving values larger than *1.0* when the calculations are on average within the experimental error bounds.

As introduced by Tzvetkova et al. [32], a second tighter indicator for the quality of a computed dataset focuses on the outliers. This outlier criterion is formulated as $1/\chi_{min}^{2}$ (i.e., the smallest value among all ($1/\chi_{i}^{2}$). A dataset is perfectly reproduced by a computed ensembles within error margins, when this the condition $1/\chi_{min}^{2}$ > 1 is met.

Finally, to convey the overall fidelity of a computed dataset, the ratio of valid data ℱ = (*n* − *n_outliers_*)/*n* will also be employed.

## Supplementary Materials

The following are available online at https://www.mdpi.com/1420-3049/24/23/4417/s1.
