# Supplementary material for: Conformational Investigations in Flexible Molecules Using Orientational NMR Constraints in Combination with 3J-Couplings and NOE Distances
Source: Molecules. 2019 Dec 3;24(23):4417. doi: 10.3390/molecules24234417 (PMC6930577; doi:10.3390/molecules24234417)
Supplement: Supplementary file 1 [file molecules-24-04417-s001.pdf]

Supplementary Information

Conformational Investigations using Orientational NMR Constraints in Combination with ^3J-Couplings and NOE Distances

Christophe Farès[1],*, Julia B. Lingnau[1], Cornelia Wirtz[1], Ulrich Sternberg [2,*]

[1] Max-Planck-Institut für Kohlenforschung, Kaiser-Wilhelm Platz 1, 45470 Mülheim an der Ruhr, Germany
[2] Research Partner of Karlsruhe Institute of Technology, Karlsruhe, Germany
* Correspondence: ulrich.sternberg@partner.kit.edu

Table of Contents

1 Classic NMR 2

1.1 1,4 diketone (**1**) 2

1.1.1 **Full NMR Assignment of 1**-*SS* 2

1.1.2 **Full NMR Assignment of 1**-*SR* 4

1.1.3 DFT- Calculation of Rotamers of **1**-*SS* and **1**-*SR* 6

1.1.4 Alignment Tensor Analysis of the 1,4 Diketone 8

1.2 Mandelalide A isomers 11

1.2.1 Full NMR Assignment of 2p 11

1.2.2 Full NMR Assignment of 11-epi-2p 14

2 Methods 17

2.1 Calculation of Dipolar Couplings 17

2.2 The Recursion Formula for the Time Average with an Exponential Decaying MemoryFunction 18

2.3 Performing MDOC Calculations 19

2.4 Analysis of MDOC Trajectories 20

2.5 Parameters for the MDOC simulation 21

3 MDOC Results 23

3.1 Results of 1,4-Diketone MDOC Simulations 23

3.2 Results of Mandelalide A MDOC Simulations 28

4 Structure ensembles 39

4.1 Mandelalide A 39

4.1.1 Mandelalide A 2p 39

4.1.2 Mandelalide A 11-epi-2A 40

5 dPCA results for mandelalide A *11-epi-2p* 41

6 References 42

1 Classic NMR

***1.1** 1,4 diketone (**1**)*

1.1.1 **Full NMR Assignment of 1-*SS***

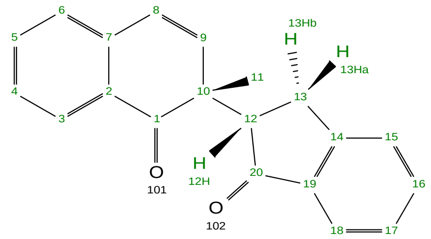

About 10mg of **1**-*SS* was dissolved in about 550uL of CDCl3 and transferred to a 5mm NMR tube. Measurements were performed on a AV-III 500 equipped with a BBFO probehead at 298K.

^{1}H NMR (500 MHz, Chloroform-*d*) δ 8.10 (d, *J* = 7.9 Hz, 1H, 3), 7.75 (d, *J* = 7.7 Hz, 1H, 18), 7.60 (td, *J* = 7.5, 1.4 Hz, 1H, 5), 7.54 (td, *J* = 7.5, 1.3 Hz, 1H, 16), 7.40 (td, *J* = 7.6, 1.2 Hz, 1H, 4), 7.36 (t, *J* = 7.5 Hz, 1H, 17), 7.31 (dt, *J* = 7.7, 0.9 Hz, 1H, 15), 7.23 (d, *J* = 7.6 Hz, 1H, 6), 6.57 (d, *J* = 9.8 Hz, 1H, 8), 6.00 (d, *J* = 9.8 Hz, 1H, 9), 3.37 (dd, *J* = 7.9, 4.6 Hz, 1H, 12H), 3.14 (dd, *J* = 17.2, 7.9 Hz, 1H, 13Hs), 2.45 (dd, *J* = 17.2, 4.6 Hz, 1H, 13Hr), 1.71 (s, 3H, 11).

^{13}C NMR (126 MHz, Chloroform-*d*) δ 207.15 (20), 201.94 (1), 153.28 (14), 138.07 (7), 137.51 (19), 135.90 (9), 135.02 (16), 134.91 (5), 128.99 (2), 128.35 (4), 127.71 (6), 127.53 (17), 127.49 (3), 126.48 (15), 126.10 (8), 123.75 (18), 52.59 (12), 52.10 (10), 31.34 (13), 23.20 (11).

Table S1: Full NMR Assignment of **1-*SS***

1-*SS*

Atom	Chemical Shift --ppm	J in Hz (coupling partner)	COSY	HSQC	HMBC	NOESY
1 C	201.94	3.90(11H), 6.70(9H), 1.90(12HH)			3, 9, 11, 12H	
2 C	128.99				4, 6, 8	
3 C	127.49			3	5	
H	8.10	1.40(5), 7.80(4)	4	3	1, 5, 7	4, 11
4 C	128.35			4	6	
H	7.40	7.50(5), 7.80(3), 1.22(6)	3, 5	4	2, 6	3, 5
5 C	134.91			5	3	
H	7.60	1.40(3), 7.50(4), 7.60(6)	4, 6	5	3, 7	4, 6
6 C	127.71			6	4, 8	
H	7.23	7.60(5), 1.22(4)	5	6	2, 4, 8	8, 5
7 C	138.07	10.70(9H)			3, 5, 9	
8 C	126.10			8	6	
H	6.57	9.80(9)	9	8	2, 6, 10	6, 9
9 C	135.90	4.60(11H), 6.20(12HH)		9	11, 12H	
H	6.00	10.70(7C), 6.70(1C), 1.80(11C), 9.80(8)	8	9	1, 7, 12	8, 11, 13Hb, 12H
10 C	52.10	3.90(11H), 5.60(12HH)			8, 13Hb, 13Ha	
11 C	23.20	1.80(9H), 3.20(12HH)		11	12H	
H3	1.71	3.90(1C), 3.90(10C), 4.60(9C), 4.20(12C)		11	1, 9, 12	12H, 3, 13Hb, 13Ha, 9
12 C	52.59	4.20(11H)		12H	9, 11	
12H H	3.37	1.90(1C), 5.60(10C), 6.20(9C), 5.80(20C), 3.20(11C), 4.60(13Hb), 7.9(13Ha)	13Ha, 13Hb	12	1, 9, 11, 20	13Hb, 13Ha, 11, 9
13 C	31.34			13Hb, 13Ha	15	
13Hb H	2.45	4.60(12H), -17.20(13Ha)	13Ha, 12H	13	10, 15, 19, 20, 14	12H, 15, 13Ha, 11, 9
13Ha H	3.14	7.90(12H), -17.20(13Hb)	12H, 13Hb	13	10, 15, 19, 20, 14	12H, 13Hb, 15, 11
14 C	153.28				13Hb, 13Ha, 16, 18	
15 C	126.48			15	13Hb, 13Ha, 17	
H	7.31	7.70(16)	16	15	13, 17, 19	13Hb, 13Ha, 16
16 C	135.02			16	18	
H	7.54	7.50(17), 1.30(18), 7.70(15)	15, 17	16	14, 18	15, 17
17 C	127.53			17	15	
H	7.36	7.70(18), 7.50(16)	16, 18	17	15, 19	16, 18
18 C	123.75			18	16	
H	7.75	7.70(17), 1.30(16)	17	18	14, 16, 20	17
19 C	137.51				13Hb, 13Ha, 15, 17	
20 C	207.15	5.80(12HH)			13Hb, 13Ha, 12H, 18	

Dataset for ***1-SS*** is available in NMReDATA[1] format here:
https://dx.doi.org/10.17617/3.34

1.1.2 **Full NMR Assignment of 1**-*SR*

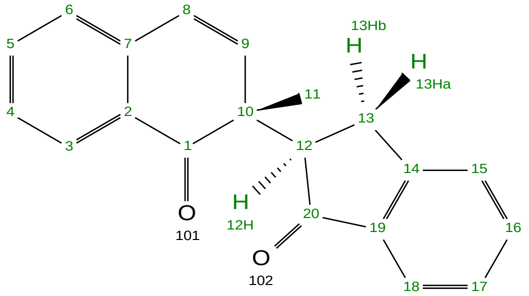

About 10mg of **1**-*SR* was dissolved in about 550uL of CDCl3 and transferred to a 5mm NMR tube. Measurements were performed on a AV-III 500 equipped with a BBFO probehead at 298K.

^{1}H NMR (500 MHz, Chloroform-*d*) δ 8.14 (d, *J* = 7.8 Hz, 1H, 3), 7.65 (d, *J* = 7.7 Hz, 1H, 18), 7.57 (t, *J* = 7.5 Hz, 1H, 5), 7.57 (t, *J* = 7.5 Hz, 1H, 16), 7.45 (dt, *J* = 7.7, 1.0 Hz, 1H, 15), 7.40 (td, *J* = 7.6, 1.2 Hz, 1H, 4), 7.34 (t, *J* = 7.5 Hz, 1H, 17), 7.24 (d, *J* = 7.6 Hz, 1H, 6), 6.59 (d, *J* = 9.8 Hz, 1H, 8), 6.03 (d, *J* = 9.9 Hz, 1H, 9), 3.61 (dd, *J* = 8.4, 5.1 Hz, 1H, 12H), 3.35 (dd, *J* = 17.3, 8.4 Hz, 1H, 13Hr), 3.08 (dd, *J* = 17.3, 5.1 Hz, 1H, 13Hs), 1.39 (s, 3H, 11).

^{13}C NMR (126 MHz, Chloroform-*d*) δ 204.93 (20), 202.08 (1), 152.73 (14), 137.78 (7), 136.83 (19), 134.81 (9), 134.78 (16), 134.33 (5), 129.73 (2), 128.27 (4), 127.69 (17), 127.65 (6), 127.15 (3), 126.46 (15), 125.57 (8), 124.02 (18), 55.75 (12), 50.13 (10), 28.67 (13), 23.07 (11).

Table S2: Full NMR-Assignment of 1-SR

1-*SR*

Atom	Chemical Shift -- ppm	J in Hz (coupling partner)	COSY	HSQC	HMBC	NOESY/EXSY
1 C	202.08	3.40(12H)			3, 11, 12H, 9	
2 C	129.73				4, 6, 8	
3 C	127.15			3	5	
H	8.14	7.80(4)	4	3	1, 5, 7	4
4 C	128.27			4	6	
H	7.40	7.60(5), 7.80(3)	3, 5	4	2, 6	3, 5
5 C	134.33			5	3	
H	7.57	7.60(4), 7.60(6)	4, 6	5	3, 7	4, 5, 8
6 C	127.65			6	4, 8	
H	7.24	7.60(5)	5	6	2, 4, 8	8
7 C	137.78				3, 5, 9, 8	
8 C	125.57			8	6	
H	6.59	9.80(9)	9	8	2, 6, 10, 7	5, 9, 6
9 C	134.81	5.00(12H)		9	11, 12H	
H	6.03	9.80(8), 5.50(12H)	8	9	1, 7, 11, 10	13Ha, 12H, 8
10 C	50.13	5.40(12HH)			11, 12H, 13Ha, 9, 8	
11 C	23.07	2.60(12HH)		11	12H, 9	
H3	1.39			11	1, 9, 12, 10	13Ha, 13Hb, 12H
12 C	55.75			12H	11, 12H, 13Ha	
12H H	3.61	3.40(1C), 5.40(10C), 5.00(9C), 5.50(9), 2.60(11C), 8.40(13Ha), 5.10(13Hb)		12	1, 9, 11, 13, 20, 10, 12	11, 13Hb, 9
13 C	28.67			13Hb, 13Ha	15, 12H, 13Hb, 13Ha	
13Hb H	3.35	17.30(13Ha), 5.10(12H)	13Ha	13	20, 14, 13	11, 13Ha, 15, 12H
13Ha H	3.08	17.30(13hb), 8.40(12H)	13Hb	13	10, 13, 12, 14	11, 13Hb, 15, 9
14 C	152.73				16, 13Hb, 13Ha, 18	
15 C	126.46			15	17	
H	7.45	7.70(16)	16	15	13, 19, 17	13Hb, 13Ha, 16
16 C	134.78			16	18	
H	7.57	7.70(15), 7.50(17)	15, 17	16	14, 18	15, 17
17 C	127.69			17	15	
H	7.34	7.50(16), 7.70(18)	16, 18	17	15, 19	16, 18
18 C	124.02			18	16, 18	
H	7.65	7.70(17)	17	18	14, 16, 20, 18	17
19 C	136.83				15, 17	
20 C	204.93				12H, 13Hb, 18	

Dataset for ***1-SR*** is available in NMReDATA[1] format here: https://dx.doi.org/10.17617/3.34

1.1.3 DFT- Calculation of Rotamers of **1**-*SS* and **1**-*SR*

For the conformation analysis of **1** based on the classical alignment method using SVD in Mspin and for the initial structures for MDOC simulations, optimized DFT structures of the two possible diastereomers (**1-*SS*** and **1-*SR***) in three different rotamers were generated with the hybrid density function B3LYP, cc-pVTZ as a basis set in Gaussian-09. The general input (preamble) is shown here:

```
$ RunGauss
%mem=25GB
%nproc=8
%chk=SR-m180_b3lyp_pvtz_geovib.chk
#p b3lyp/cc-pVTZ
   density=current integral(ultrafine)
   scf=(direct,conver=10,maxcycle=500)
   cphf=(simultaneous,conver=10)
   maxdisk=100GB
   opt=(readfc,verytight) freq
   guess=read geom=check

 SR-m180    B3LYP/PVTZ   GEO-OPT + VIB
```

Table S3

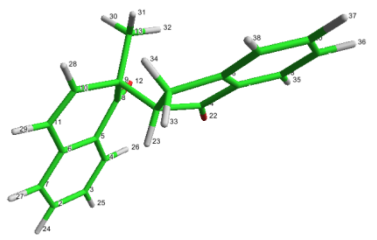

```
# SS-p60 MP2/PVTZ
# Created by GaussView 3.09

@<TRIPOS>MOLECULE
Molecule Name
38 41
SMALL
NO_CHARGES


@<TRIPOS>ATOM
1 C1    -0.5044     0.3960    -0.3299 C
2 C2     4.8672    -0.3570    -1.3540 C
3 C3     4.4060    -1.5585    -0.8207 C
4 C4     3.2731    -1.5675    -0.0192 C
5 C5     2.5801    -0.3856     0.2234 C
6 C6     3.0349     0.8308    -0.3172 C
7 C7     4.1943     0.8292    -1.0967 C
8 C8     1.4063    -0.3747     1.1393 C
9 C9     0.3810     0.7602     0.9304 C
10 C10     1.1097     2.0404     0.5816 C
11 C11     2.3059     2.0572    -0.0113 C
12 O12     1.2848    -1.1915     2.0236 O
13 C13    -0.4661     0.9224     2.1971 C
14 C14    -1.2555    -0.9454    -0.1985 C
15 C15    -2.7014    -0.6640    -0.3014 C
16 C16    -2.9070     0.6856    -0.5691 C
17 C17    -1.6011     1.4382    -0.6684 C
18 C18    -3.7615    -1.5602    -0.1864 C
19 C19    -5.0506    -1.0753    -0.3355 C
20 C20    -5.2651     0.2811    -0.6023 C
21 C21    -4.2016     1.1683    -0.7243 C
22 O22    -0.7320    -2.0295    -0.0862 O
23 H23     0.1820     0.2810    -1.1692 H
24 H24     5.7581    -0.3460    -1.9679 H
25 H25     4.9332    -2.4804    -1.0251 H
26 H26     2.9057    -2.4820     0.4246 H
27 H27     4.5662     1.7629    -1.4994 H
28 H28     0.5962     2.9707     0.7919 H
29 H29     2.7648     2.9987    -0.2879 H
30 H30     0.1697     1.1867     3.0412 H
31 H31    -1.2094     1.7103     2.0770 H
32 H32    -0.9726    -0.0056     2.4509 H
33 H33    -1.4624     1.8357    -1.6757 H
34 H34    -1.5923     2.2976     0.0012 H
35 H35    -3.5654    -2.6045     0.0163 H
36 H36    -5.8971    -1.7427    -0.2471 H
37 H37    -6.2779     0.6447    -0.7167 H
38 H38    -4.3855     2.2142    -0.9355 H
```

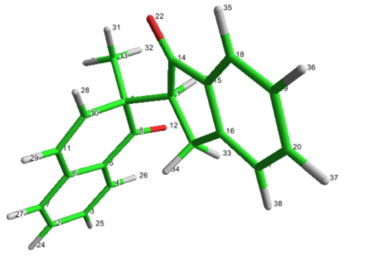

```
#SS-180 MP2/PVTZ
# Created by GaussView 3.09

@<TRIPOS>MOLECULE
Molecule Name
38 41
SMALL
NO_CHARGES


@<TRIPOS>ATOM
1 C1     0.8840     0.7571    -0.6811 C
2 C2    -4.9088    -1.4058     0.8045 C
3 C3    -4.8757    -1.2188    -0.5770 C
4 C4    -3.7923    -0.5792    -1.1559 C
5 C5    -2.7402    -0.1129    -0.3659 C
6 C6    -2.7696    -0.2971     1.0298 C
7 C7    -3.8667    -0.9523     1.5981 C
8 C8    -1.5812     0.5482    -1.0122 C
9 C9    -0.4995     1.1939    -0.1236 C
10 C10    -0.6324     0.8509     1.3344 C
11 C11    -1.6689     0.1875     1.8496 C
12 O12    -1.4810     0.6133    -2.2241 O
13 C13    -0.6929     2.7287    -0.2853 C
14 C14     2.1182     1.2658     0.0901 C
15 C15     3.1021     0.1640     0.1110 C
16 C16     2.5357    -0.9943    -0.4134 C
17 C17     1.0957    -0.7722    -0.7998 C
18 C18     4.4159     0.1964     0.5726 C
19 C19     5.1697    -0.9629     0.4894 C
20 C20     4.6095    -2.1288    -0.0438 C
21 C21     3.2951    -2.1560    -0.4960 C
22 O22     2.2689     2.3668     0.5752 O
23 H23     0.9641     1.1895    -1.6820 H
24 H24    -5.7517    -1.9078     1.2610 H
25 H25    -5.6915    -1.5725    -1.1926 H
26 H26    -3.7357    -0.4262    -2.2244 H
27 H27    -3.8964    -1.1020     2.6698 H
28 H28     0.1505     1.2207     1.9849 H
29 H29    -1.7225     0.0043     2.9159 H
30 H30    -1.6799     3.0255     0.0704 H
31 H31     0.0651     3.2627     0.2807 H
32 H32    -0.6084     2.9957    -1.3383 H
33 H33     0.8708    -1.1392    -1.8007 H
34 H34     0.4397    -1.3106    -0.1098 H
35 H35     4.8234     1.1128     0.9780 H
36 H36     6.1953    -0.9717     0.8329 H
37 H37     5.2121    -3.0258    -0.1042 H
38 H38     2.8761    -3.0676    -0.9024 H
```

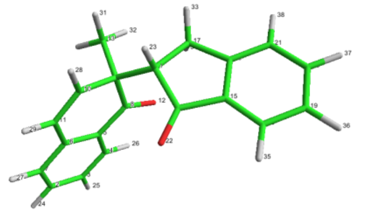

```
# SS-m60 MP2/PVTZ
# Created by GaussView 3.09

@<TRIPOS>MOLECULE
Molecule Name
38 41
SMALL
NO_CHARGES


@<TRIPOS>ATOM
1 C1     0.9704     1.1940     0.3716 C
2 C2    -4.9287    -1.3907    -0.0097 C
3 C3    -4.2348    -1.7767    -1.1560 C
4 C4    -2.9808    -1.2462    -1.4082 C
5 C5    -2.4140    -0.3202    -0.5311 C
6 C6    -3.1102     0.0733     0.6275 C
7 C7    -4.3713    -0.4791     0.8730 C
8 C8    -1.0625     0.2153    -0.8165 C
9 C9    -0.5350     1.3811     0.0403 C
10 C10    -1.3210     1.5824     1.3098 C
11 C11    -2.5034     1.0181     1.5504 C
12 O12    -0.3924    -0.2121    -1.7403 O
13 C13    -0.7307     2.6666    -0.8147 C
14 C14     1.3112    -0.1700     1.0033 C
15 C15     2.6758    -0.5188     0.5630 C
16 C16     3.1018     0.3808    -0.4110 C
17 C17     2.0248     1.3824    -0.7471 C
18 C18     3.4791    -1.5713     0.9938 C
19 C19     4.7428    -1.7021     0.4408 C
20 C20     5.1824    -0.7953    -0.5292 C
21 C21     4.3703     0.2464    -0.9634 C
22 O22     0.5897    -0.8120     1.7326 O
23 H23     1.2018     1.9210     1.1591 H
24 H24    -5.9063    -1.8071     0.1952 H
25 H25    -4.6727    -2.4887    -1.8423 H
26 H26    -2.4151    -1.5352    -2.2826 H
27 H27    -4.9102    -0.1899     1.7662 H
28 H28    -0.8984     2.2778     2.0261 H
29 H29    -3.0401     1.2442     2.4632 H
30 H30    -1.7887     2.8342    -1.0141 H
31 H31    -0.3474     3.5356    -0.2784 H
32 H32    -0.2090     2.5798    -1.7660 H
33 H33     2.4110     2.4014    -0.7935 H
34 H34     1.5972     1.1397    -1.7187 H
35 H35     3.1130    -2.2583     1.7449 H
36 H36     5.3956    -2.5043     0.7575 H
37 H37     6.1723    -0.9102    -0.9514 H
38 H38     4.7228     0.9333    -1.7223 H
```

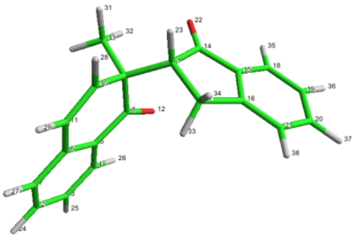

```
# SR-p60 MP2/PVTZ
# Created by GaussView 3.09

@<TRIPOS>MOLECULE
Molecule Name
38 41
SMALL
NO_CHARGES


@<TRIPOS>ATOM
1 C1     0.9239     1.2975     0.5893 C
2 C2    -4.8243    -1.5635    -0.0630 C
3 C3    -4.0449    -1.9569    -1.1498 C
4 C4    -2.7927    -1.3936    -1.3354 C
5 C5    -2.3170    -0.4232    -0.4534 C
6 C6    -3.1010    -0.0198     0.6443 C
7 C7    -4.3553    -0.6094     0.8272 C
8 C8    -0.9568     0.1392    -0.6470 C
9 C9    -0.5631     1.3928     0.1502 C
10 C10     -1.4295     1.6090     1.3639 C
11 C11     -2.5884     0.9816     1.5692 C
12 O12     -0.1733    -0.3537    -1.4361 O
13 C13     -0.8024     2.6032    -0.7993 C
14 C14      1.9821     1.2353    -0.5396 C
15 C15      2.8801     0.1015    -0.2398 C
16 C16      2.4819    -0.5387     0.9277 C
17 C17      1.2364     0.0864     1.5056 C
18 C18      4.0002    -0.3099    -0.9563 C
19 C19      4.7254    -1.3903    -0.4804 C
20 C20      4.3325    -2.0374     0.6956 C
21 C21      3.2142    -1.6185     1.4077 C
22 O22      2.1151     2.0368    -1.4360 O
23 H23      1.1439     2.2264     1.1236 H
24 H24     -5.7999    -2.0058     0.0895 H
25 H25     -4.4148    -2.7004    -1.8425 H
26 H26     -2.1605    -1.6902    -2.1605 H
27 H27     -4.9617    -0.3130     1.6736 H
28 H28     -1.0922     2.3669     2.0621 H
29 H29     -3.1920     1.2184     2.4368 H
30 H30     -1.8542     2.6559    -1.0802 H
31 H31     -0.5402     3.5305    -0.2889 H
32 H32     -0.1873     2.5171    -1.6899 H
33 H33      0.4169    -0.6353     1.5121 H
34 H34      1.3856     0.3883     2.5437 H
35 H35      4.2823     0.2123    -1.8605 H
36 H36      5.5996    -1.7374    -1.0147 H
37 H37      4.9097    -2.8788     1.0563 H
38 H38      2.9227    -2.1304     2.3162 H
```

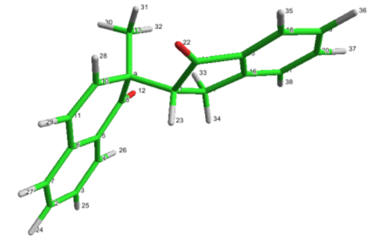

```
# SR-m180 MP2/PVTZ
# Created by GaussView 3.09

@<TRIPOS>MOLECULE
Molecule Name
38 41
SMALL
NO_CHARGES


@<TRIPOS>ATOM
1 C1    -0.6084     0.2015    -0.1396 C
2 C2     5.0734    -0.4272    -1.2929 C
3 C3     4.6488    -1.6278    -0.7247 C
4 C4     3.4764    -1.6596     0.0123 C
5 C5     2.7088    -0.5047     0.1698 C
6 C6     3.1319     0.7097    -0.4022 C
7 C7     4.3273     0.7294    -1.1280 C
8 C8     1.4770    -0.5500     0.9950 C
9 C9     0.4907     0.6331     0.9086 C
10 C10      1.1489     1.8928     0.4014 C
11 C11      2.3386     1.9126    -0.2020 C
12 O12      1.2281    -1.5095     1.7023 O
13 C13     -0.1316     0.8818     2.2942 C
14 C14     -1.7887     1.1950    -0.2662 C
15 C15     -3.0219     0.3950    -0.3838 C
16 C16     -2.7361    -0.9561    -0.2133 C
17 C17     -1.2736    -1.1816     0.0697 C
18 C18     -4.3170     0.8498    -0.6236 C
19 C19     -5.3362    -0.0845    -0.7019 C
20 C20     -5.0555    -1.4457    -0.5387 C
21 C21     -3.7620    -1.8916    -0.2935 C
22 O22     -1.7130     2.4037    -0.2876 O
23 H23     -0.1289     0.2124    -1.1228 H
24 H24      5.9929    -0.3957    -1.8626 H
25 H25      5.2346    -2.5275    -0.8548 H
26 H26      3.1330    -2.5715     0.4801 H
27 H27      4.6672     1.6611    -1.5620 H
28 H28      0.5768     2.8024     0.5086 H
29 H29      2.7441     2.8470    -0.5701 H
30 H30      0.6454     1.1388     3.0139 H
31 H31     -0.8380     1.7091     2.2489 H
32 H32     -0.6395    -0.0054     2.6645 H
33 H33     -1.1251    -1.5443     1.0867 H
34 H34     -0.8454    -1.9418    -0.5846 H
35 H35     -4.5036     1.9081    -0.7471 H
36 H36     -6.3527     0.2325    -0.8916 H
37 H37     -5.8627    -2.1638    -0.6035 H
38 H38     -3.5633    -2.9478    -0.1649 H
```

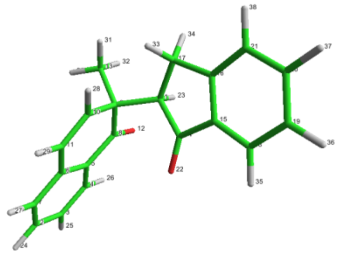

```
# SR-m60 MP2/PVTZ
# Created by GaussView 3.09

@<TRIPOS>MOLECULE
Molecule Name
38 41
SMALL
NO_CHARGES


@<TRIPOS>ATOM
1 C1     0.9359     0.9825    -0.4537 C
2 C2    -4.8242    -1.4859     0.5253 C
3 C3    -4.7793    -1.0384    -0.7941 C
4 C4    -3.7058    -0.2754    -1.2238 C
5 C5    -2.6733     0.0510    -0.3449 C
6 C6    -2.7136    -0.3976     0.9878 C
7 C7    -3.8015    -1.1694     1.4061 C
8 C8    -1.5322     0.8588    -0.8340 C
9 C9    -0.4467     1.3080     0.1724 C
10 C10     -0.5960     0.6943     1.5365 C
11 C11     -1.6315    -0.0620     1.9006 C
12 O12     -1.4597     1.2322    -1.9895 O
13 C13     -0.6108     2.8458     0.3006 C
14 C14      1.2098    -0.5251    -0.5739 C
15 C15      2.6482    -0.7316    -0.3055 C
16 C16      3.2227     0.4599     0.1299 C
17 C17      2.1980     1.5670     0.2210 C
18 C18      3.3916    -1.9023    -0.4297 C
19 C19      4.7419    -1.8594    -0.1210 C
20 C20      5.3274    -0.6641     0.3084 C
21 C21      4.5773     0.4996     0.4390 C
22 O22      0.3882    -1.3708    -0.8461 O
23 H23      0.8861     1.3470    -1.4839 H
24 H24     -5.6591    -2.0850     0.8645 H
25 H25     -5.5777    -1.2882    -1.4795 H
26 H26     -3.6408     0.0790    -2.2426 H
27 H27     -3.8379    -1.5224     2.4288 H
28 H28      0.1785     0.9212     2.2603 H
29 H29     -1.6911    -0.4527     2.9092 H
30 H30     -1.6010     3.0933     0.6833 H
31 H31      0.1252     3.2586     0.9901 H
32 H32     -0.4899     3.3141    -0.6754 H
33 H33      2.0196     1.8191     1.2691 H
34 H34      2.5421     2.4818    -0.2626 H
35 H35      2.9132    -2.8126    -0.7652 H
36 H36      5.3500    -2.7491    -0.2134 H
37 H37      6.3835    -0.6461     0.5438 H
38 H38      5.0456     1.4154     0.7769 H
```

Table S4: Relative energies (kcal/mol) of the rotameric forms (gauche(+), trans, gauce(-)) of **1-*SS*** and **1-*SR*** evaluated at the B3LYP/cc-pVTZ and at the MP2(fc)/cc-pVTZ levels of theory. Values in parenthesis were the calculated differences of the respective internal energies (enthalpies) at T=0K.

		**1-*SS***			**1-*SR***	
rotamer	B3LYP	ω (°)	MP2(fc)	B3LYP	ω (°)	MP2(fc)
g-	2.31(2.20)	-53.0	2.34(2.23)	0.00(0.00)	-67.9	0.00(0.00)
t	0.00(0.00)	-178.4	0.00(0.00)	0.49(0.44)	173.8	0.72(0.67)
g+	5.78(5.61)	58.6	3.59(3.42)	0.97(0.92)	62.3	0.99(1.05)

Table S5: Input Data for MSPIN

```
rdc_data {
#HGS-HB-141-11_pmma1 for SR(RS)
#I J value error
#C11H11
13 30   3.08 1.53
13 31   3.08 1.53
13 32   3.08 1.53
#C13H13cis
17 34 -11.05 0.95
#C13H13trans
17 33   2.80 0.60
#C12H12
 1 23 -14.95 0.45
#C18H18
18 35   7.10 0.70
```


```
rdc_data {
#HGS-HB-141-11_pmma1 for SR(RS)
#I J value error
#C11H11
13 30   3.08 1.53
13 31   3.08 1.53
13 32   3.08 1.53
#C13H13cis
17 34 -11.05 0.95
#C13H13trans
17 33   2.80 0.60
#C12H12
 1 23 -14.95 0.45
#C18H18
18 35   7.10 0.70
```

#C8H8<br>11 29 -12.00 0.90<br>#C15H15<br>21 38 6.90 1.10<br>#C3H3<br>4 26 -11.60 1.55<br>#C17H17<br>19 36 19.70 4.00<br>#C6H6<br>7 27 -9.75 1.15<br>#C4H4<br>3 25 21.50 1.40<br>#C5H5<br>2 24 13.50 1.60<br>#C16H16<br>20 37 -5.75 0.55<br>#C9H9<br>10 28 21.50 0.45<br>#H3H4<br>26 25 4.84 0.40<br>#H18H17<br>35 36 -2.40 0.40<br>#H15H16<br>38 37 8.83 0.40<br>#H16H17<br>37 36 2.10 0.40<br>#H5H6<br>24 27 8.10 0.45<br>#H6H8<br>27 29 10.20 0.90<br>#H8H9<br>29 28 5.55 0.42<br>#H13cisH13trans<br>34 33 -14.40 0.52<br>}	#C8H8<br>11 29 -12.00 0.90<br>#C15H15<br>21 38 6.90 1.10<br>#C3H3<br>4 26 -11.60 1.55<br>#C17H17<br>19 36 19.70 4.00<br>#C6H6<br>7 27 -9.75 1.15<br>#C4H4<br>3 25 21.50 1.40<br>#C5H5<br>2 24 13.50 1.60<br>#C16H16<br>20 37 -5.75 0.55<br>#C9H9<br>10 28 21.50 0.45<br>#H3H4<br>26 25 4.84 0.40<br>#H18H17<br>35 36 -2.40 0.40<br>#H15H16<br>38 37 8.83 0.40<br>#H16H17<br>37 36 2.10 0.40<br>#H5H6<br>24 27 8.10 0.45<br>#H6H8<br>27 29 10.20 0.90<br>#H8H9<br>29 28 5.55 0.42<br>#H13cisH13trans<br>34 33 -14.40 0.52<br>}
rdc_data {<br>#HGS-HB-141-12_pmma1 for SR(RS)<br>#I J value error<br>#C11H11<br>13 30 3.28 0.13<br>13 31 3.28 0.13<br>13 32 3.28 0.13<br>#C13H13cis<br>17 34 -4.10 1.25<br>#C13H13trans<br>17 33 -20.30 1.30<br>#C12H12<br>1 23 -15.55 0.35<br>#C18H18<br>18 35 0.25 0.10<br>#C8H8<br>11 29 -11.60 0.40<br>#C15H15<br>21 38 -0.85 0.95<br>#C3H3<br>4 26 -11.95 0.20<br>#C17H17<br>19 36 3.25 0.55<br>#C6H6<br>7 27 -12.10 0.50<br>#C4H4<br>3 25 12.30 0.70<br>#C5H5<br>2 24 18.55 1.50<br>#C16H16<br>20 37 24.35 0.95<br>#C9H9<br>10 28 10.43 0.72<br>#H6H8<br>27 29 8.60 0.70<br>#H8H9<br>29 28 7.14 0.50<br>#H13cisH13trans<br>34 33 -13.95 1.00<br>#H12H13cis<br>17 34 3.90 0.45<br>#H3H4<br>26 25 6.60 0.60<br>#H18H17<br>35 36 8.55 0.80<br>#H15H16<br>38 37 0.80 0.90<br>#H16H17<br>37 36 -0.30 1.00<br>#H5H6<br>24 27 4.30 1.20<br>#H4H5<br>25 24 -4.90 1.20<br>#H9H13trans<br>#28 33 4.80 1.30<br>}38 H38 2.9227 -2.1304 2.3162 H	rdc_data {<br>#HGS-HB-141-12_pmma1 for SS(RR)<br>#I J value error<br>#C11H11<br>13 30 3.28 0.13<br>13 31 3.28 0.13<br>13 32 3.28 0.13<br>#C13H13cis<br>17 33 -4.10 1.25<br>#C13H13trans<br>17 34 -20.30 1.30<br>#C12H12<br>1 23 -15.55 0.35<br>#C18H18<br>18 35 0.25 0.10<br>#C8H8<br>11 29 -11.60 0.40<br>#C15H15<br>21 38 -0.85 0.95<br>#C3H3<br>4 26 -11.95 0.20<br>#C17H17<br>19 36 3.25 0.55<br>#C6H6<br>7 27 -12.10 0.50<br>#C4H4<br>3 25 12.30 0.70<br>#C5H5<br>2 24 18.55 1.50<br>#C16H16<br>20 37 24.35 0.95<br>#C9H9<br>10 28 10.43 0.72<br>#H6H8<br>27 29 8.60 0.70<br>#H8H9<br>29 28 7.14 0.50<br>#H13cisH13trans<br>33 34 -13.95 1.00<br>#H12H13cis<br>17 33 3.90 0.45<br>#H3H4<br>26 25 6.60 0.60<br>#H18H17<br>35 36 8.55 0.80<br>#H15H16<br>38 37 0.80 0.90<br>#H16H17<br>37 36 -0.30 1.00<br>#H5H6<br>24 27 4.30 1.20<br>#H4H5<br>25 24 -4.90 1.20<br>#H9H13trans<br>#28 34 4.80 1.30<br>}-3.5633 -2.9478 -0.1649 H

1.1.4 Alignment Tensor Analysis of the 1,4 Diketone

Table S6: Results from MSPIN.

DATASET 1A					1-SS-t	1-SS-g-	1-SS-g+	1-SS (t, g-, g+)		1-SR-t	1-SR-g-	1-SR-g+	1-SR (t, g-, g+)
				trans	100.0%			69.6%		100.0%			7070.0%
				gauche-		100.0%		0.0%			100.0%		19.0%
				gauche+			100.0%	30.4%				100.0%	10.2%
	**atom#**	exp	err		comp	comp	comp	comp		comp	comp	comp	comp
**#C11H11**	**9,13**	-1.02	0.50		-0.65	0.20	-0.29	-1.93		-0.96	0.16	-0.28	-1.38
**#C13H13cis**	**17,33**	-11.05	0.95		-7.58	-4.18	-21.74	-7.47		-3.61	-9.86	-5.86	-10.83
**#C13H13trans**	**17,34**	2.80	0.60		1.85	9.39	9.13	2.48		-3.80	-1.95	11.18	1.98
**#C12H12**	**1,23**	-14.95	0.45		-11.08	-5.88	-0.35	-14.15		-9.44	-11.69	0.63	-12.11
**#C18H18**	**18,35**	7.10	0.70		6.36	0.47	4.78	5.03		5.30	10.53	-0.39	6.97
**#C8H8**	**11,29**	-12.00	0.90		-13.71	-13.91	0.68	-11.24		-9.20	-13.56	-2.89	-11.42
**#C15H15**	**21,38**	6.90	1.10		6.80	0.59	4.43	5.23		5.88	10.47	-0.36	7.86
**#C3H3**	**4,26**	-11.60	1.55		-14.08	-15.33	-4.43	-12.60		-7.22	-13.22	-3.01	-7.38
**#C17H17**	**19,36**	19.70	4.00		21.95	2.19	5.80	18.65		28.72	0.99	-0.64	39.99
**#C6H6**	**7,27**	-9.75	1.15		-13.72	-15.10	-3.09	-12.14		-7.45	-12.86	-3.27	-8.05
**#C4H4**	**3,25**	21.50	1.40		19.91	0.41	15.95	24.01		17.70	11.25	0.43	21.53
**#C5H5**	**2,24**	13.50	1.60		12.17	5.95	5.13	14.85		7.26	-2.95	11.22	8.13
**#C16H16**	**20,37**	-5.75	0.55		-10.54	-4.42	10.58	-3.44		-11.56	7.82	-5.52	-18.65
**#C9H9**	**10,28**	21.50	0.45		18.35	0.30	15.87	22.89		17.75	10.61	0.05	21.90
**#H3H4**	**26,25**	4.84	0.40		4.08	2.04	1.59	4.91		2.34	-0.88	3.65	2.60
**#H18H17**	**35,36**	-2.40	0.40		-3.45	-1.43	3.42	-1.18		-3.81	2.56	-1.78	-6.12
**#H15H16**	**38,37**	8.83	0.40		7.52	0.84	1.87	6.34		9.78	0.36	-0.16	13.66
**#H16H17**	**37,36**	2.10	0.40		2.47	0.23	1.52	1.92		2.21	3.47	-0.10	2.96
**#H5H6**	**24,27**	8.10	0.45		6.66	0.15	5.34	8.00		5.86	3.76	0.13	7.14
**#H6H8**	**27,29**	10.20	0.90		8.93	3.39	4.70	9.91		5.88	3.44	2.90	6.72
**#H8H9**	**29,28**	5.55	0.42		4.82	2.14	2.91	6.01		3.65	-0.64	4.11	4.29
**#H13cisH13trans**	**33,34**	-14.40	0.52		-14.55	0.80	-10.18	-10.12		-16.42	-14.07	2.93	-22.87
			**Q**		0.190	0.833	0.694	0.158		0.368	0.682	0.929	0.517
			**n/X2**		0.07	0.00	0.01	0.11		0.03	0.01	0.00	0.02

DATASET 1B					1-SR-t	1-SR-g-	1-SR-g+	1-SR (t, g-, g+)		1-SS-t	1-SS-g-	1-SS-g+	1-SS (t, g-, g+)
				trans	100.0%			0.0%		100.0%			27.0%
				gauche-		100.0%		40.7%			100.0%		68.6%
				gauche+			100.0%	59.3%				100.0%	4.4%
	**atom#**	exp	err		comp	comp	comp	comp		comp	comp	comp	comp
**#C11H11**	**9,13**	-1.09	0.13		-0.88	-0.66	0.25	-1.83		-0.68	-0.74	-0.23	-0.63
**#C13H13cis**	**17,34**	-4.10	1.25		5.41	0.02	-1.20	-0.49		-2.10	3.15	-5.91	-5.57
**#C13H13trans**	**17,33**	-20.30	1.30		-27.54	-22.11	-4.88	-20.74		-8.81	-11.82	-20.29	-20.81
**#C12H12**	**1,23**	-15.55	0.35		-12.37	-13.09	-10.28	-17.81		-20.35	-10.72	0.75	-1.25
**#C18H18**	**18,35**	0.25	0.10		-2.98	0.11	-6.14	-0.20		1.45	-5.22	0.53	-1.63
**#C8H8**	**11,29**	-11.60	0.40		-4.02	-13.67	-10.35	-9.24		-15.53	-10.79	-10.92	-14.50
**#C15H15**	**21,38**	-0.85	0.95		-3.09	-0.29	-6.29	-0.52		1.48	-5.70	0.21	-1.70
**#C3H3**	**4,26**	-11.95	0.20		-8.74	-14.48	-10.07	-13.20		-14.81	-9.66	-8.28	-9.80
**#C17H17**	**19,36**	3.25	0.55		15.22	-1.41	-3.29	3.54		11.92	0.31	-4.19	0.66
**#C6H6**	**7,27**	-12.10	0.50		-7.64	-14.33	-10.27	-12.45		-14.89	-10.73	-8.87	-10.68
**#C4H4**	**3,25**	12.30	0.70		6.69	10.61	-5.40	12.23		5.00	-3.99	7.04	12.01
**#C5H5**	**2,24**	18.55	1.50		11.22	14.58	-0.82	19.19		0.18	23.44	9.87	11.26
**#C16H16**	**20,37**	24.35	0.95		16.42	25.99	10.42	22.70		7.61	16.33	28.38	24.46
**#C9H9**	**10,28**	10.43	0.72		6.10	8.86	-6.11	10.93		4.24	-5.08	6.23	10.31
**#H6H8**	**27,29**	8.60	0.70		4.63	7.53	-0.05	8.33		2.94	5.58	3.70	5.48
**#H8H9**	**29,28**	7.14	0.50		5.22	5.55	-0.95	8.02		-0.13	8.18	5.00	5.12
**#H13cisH13trans**	**34,33**	-13.95	1.00		-13.75	-10.66	0.69	-12.28		-9.65	0.37	-15.80	-17.94
**#H12H13cis**	**17,34**	3.90	0.45		5.41	0.02	-1.20	-0.49		-2.10	3.15	-5.91	-5.57
**#H3H4**	**26,25**	6.60	0.60		3.55	4.86	-0.14	6.23		0.19	7.73	3.07	3.62
**#H18H17**	**35,36**	8.55	0.80		5.24	8.39	3.32	7.30		2.40	5.26	9.17	7.89
**#H15H16**	**38,37**	0.80	0.90		4.88	-0.74	-1.26	0.95		3.93	-0.10	-1.67	-0.02
**#H16H17**	**37,36**	-0.30	1.00		-1.01	-0.24	-2.20	-0.25		0.54	-1.94	-0.15	-0.76
**#H5H6**	**24,27**	4.30	1.20		2.23	3.56	-1.82	4.08		1.66	-1.37	2.28	3.93
**#H4H5**	**25,24**	-4.90	1.20		-2.67	-4.78	-3.41	-4.22		-4.92	-3.56	-2.89	-3.43
			**Q**		0.476	0.210	0.857	0.140		0.660	0.620	0.479	0.387
			**n/X2**		0.01	0.05	0.00	0.08		0.01	0.00	0.01	0.01

Back-computed values in black are within the estimated error margins for each coupling. The red values are outside these margins. Q and n/chi2 values are color-coded from blue to red according to the quality of the results

A

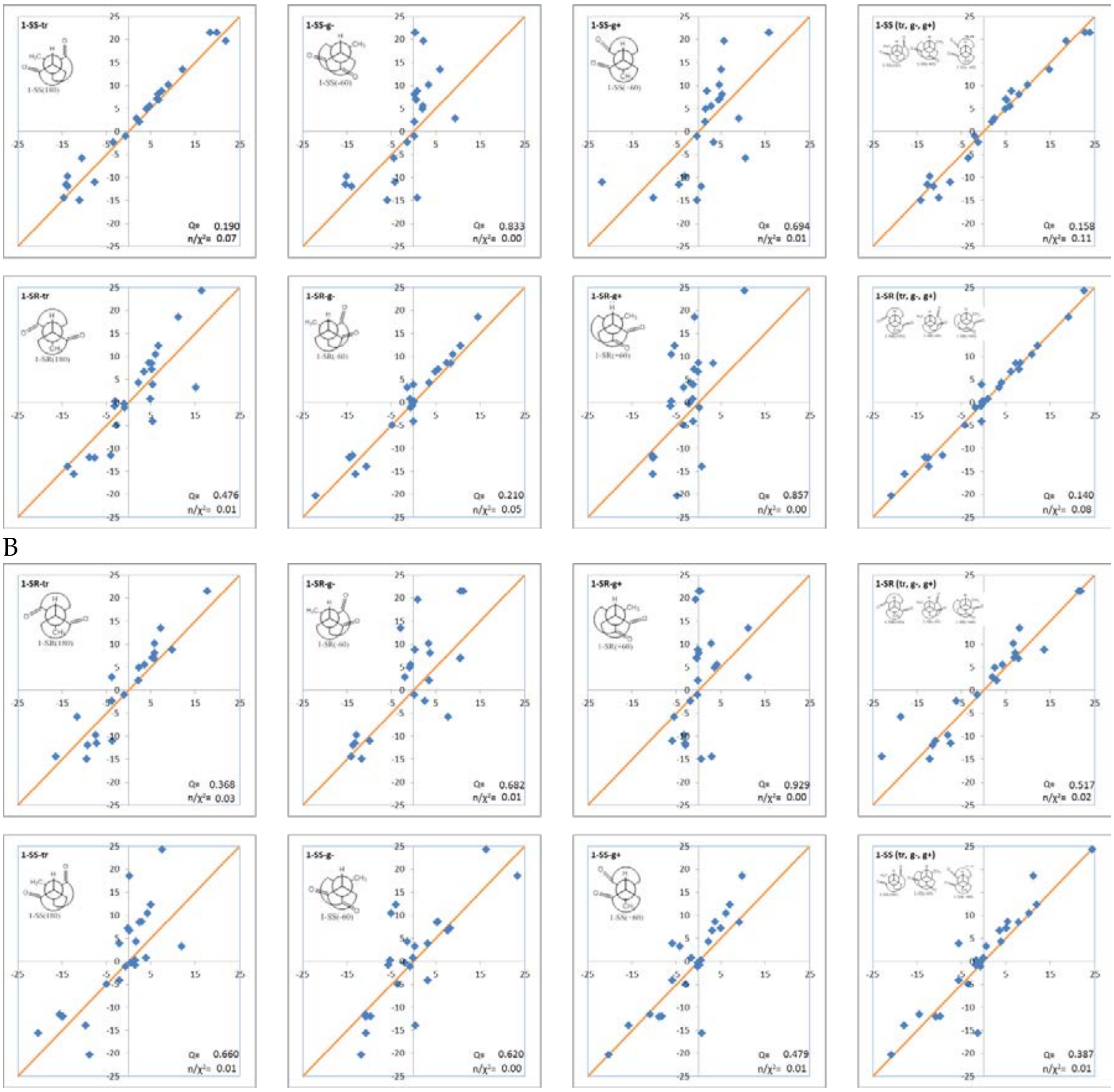

Figure S1: Correlation plot of experimental (A) dataset 1A and (B) dataset 1B against SVD-predicted RDCs on each of the DFT-calculated rotamers of diastereomers ***1-SR*** and **1-*SS***.

1.2 Mandelalide A isomers

1.2.1 Full NMR Assignment of 2p

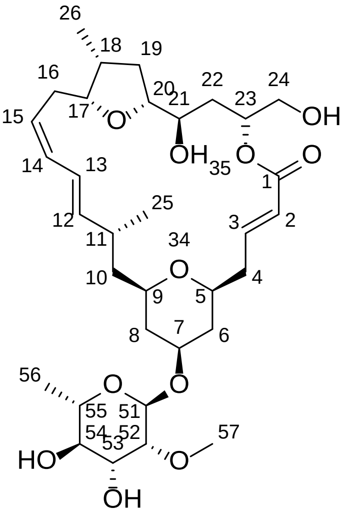

About 1mg of 2*p* was dissolved in about 60uL of CDCl3 and transferred to a 3mm NMR tube. Measurements were performed on a AV 600 equipped with a TCI probehead at 298K.

^{1}H NMR (600 MHz, Chloroform-*d*) δ 7.04 (dt, *J* = 15.8, 7.0 Hz, 1H, 3), 6.35 (ddt, *J* = 15.2, 10.8, 1.3 Hz, 1H, 13), 6.08 (t, *J* = 10.9 Hz, 1H, 14), 5.91 (dt, *J* = 15.7, 1.4 Hz, 1H, 2), 5.73 (dd, *J* = 15.2, 6.5 Hz, 1H, 12), 5.36 (dt, *J* = 10.9, 8.0 Hz, 1H, 15), 5.01 (d, *J* = 1.7 Hz, 1H, 51), 4.14 (m, 2H, 24a), 4.08 (dtd, *J* = 8.6, 5.4, 3.5 Hz, 1H), 4.06 (q, *J* = 7.1, 5.9 Hz, 1H), 3.77 (m, 2H, 7, 20), 3.68 (m, 2H, 21, 53), 3.62 (dq, *J* = 9.4, 6.2 Hz, 1H, 55), 3.45 (s, 3H, 57), 3.39 (dd, *J* = 3.8, 1.5 Hz, 1H, 52), 3.33 (m, 3H, 5, 9, 54), 2.52 (dp, *J* = 12.0, 6.6 Hz, 1H, 11), 2.44 (dh, *J* = 10.8, 7.3 Hz, 1H, 18), 2.38 (dddd, *J* = 15.0, 9.7, 6.7, 1.5 Hz, 1H), 2.32 (dddd, *J* = 15.0, 7.3, 3.2, 1.3 Hz, 1H), 2.29 (m, 2H), 1.99 (ddt, *J* = 12.2, 3.9, 1.7 Hz, 1H, 6''), 1.94 (ddd, *J* = 12.1, 7.3, 6.1 Hz, 1H), 1.86 (ddq, *J* = 12.4, 3.7, 1.7 Hz, 1H, 8''), 1.70 (ddd, *J* = 14.3, 8.8, 3.2 Hz, 1H, 22''), 1.64 (ddd, *J* = 14.0, 10.3, 4.8 Hz, 1H, 10'), 1.59 (ddd, *J* = 14.3, 8.5, 3.5 Hz, 1H), 1.47 (ddd, *J* = 12.2, 10.8, 9.6 Hz, 1H, 19''), 1.26 (d, *J* = 6.3 Hz, 3H, 8', 56), 1.23 (m, 2H), 1.19 (q, *J* = 12.1, 11.3 Hz, 1H), 1.04 (d, *J* = 7.0 Hz, 3H, 26), 1.01 (d, *J* = 6.6 Hz, 3H, 25).

^{13}C NMR (151 MHz, Chloroform-*d*) δ 166.43 (1), 146.04 (3), 141.98 (12), 130.94 (14), 126.39 (15), 123.07 (13), 122.78 (2), 93.96 (51), 81.45 (20), 80.80 (17), 80.60 (52), 74.20 (54), 73.99 (5), 73.34, 72.69 (7), 71.43 (21), 71.15 (53), 68.10 (23), 67.95 (24), 67.93 (55), 58.89 (57), 42.78 (10), 39.33 (8), 38.14 (4), 37.49 (6), 37.19 (22), 36.89 (18), 35.56 (19), 32.73 (11), 30.42 (16), 17.99 (25), 17.47 (56), 14.49 (26).

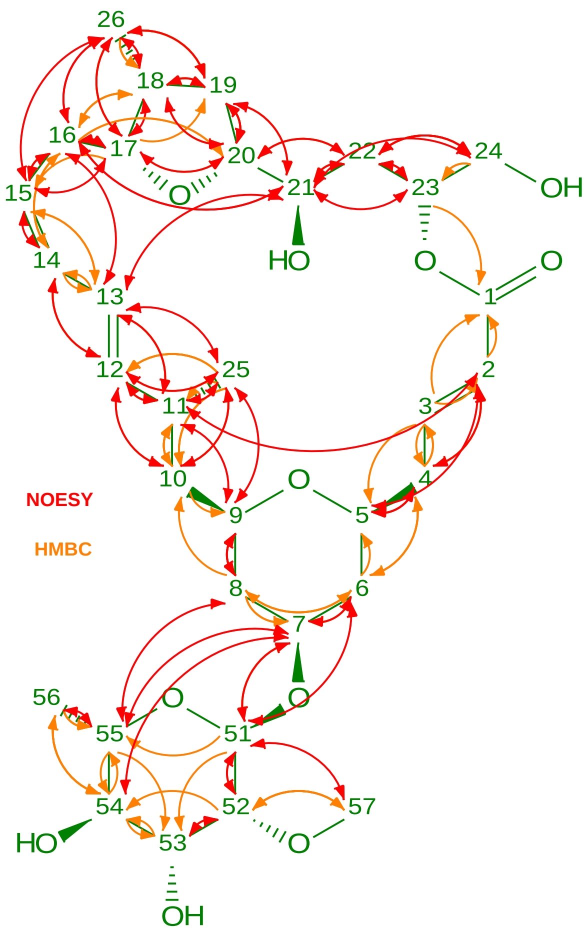

Summary of correlations for ***2p***

Table S7: Full NMR-Assignment of ***2p***

Assignments (2p)							
Atom	Chemical Shift --ppm	Predicted Shift --ppm	J in Hz (coupling partner)	COSY	HSQC	HMBC	NOESY
1 C	166.43	166.98				2, 3, 23	
2 C	122.78	122.4			2	3, 4', 4''	
H	5.91	5.83	15.80(3)	3	2	1, 4	4', 4'', 5, 11
3 C	146.04	141.02			3	4', 4''	
H	7.04	6.88	15.80(2), 6.70(4'), 7.30(4'')	2, 4', 4''	3	1, 2, 4, 5	
4 C	38.14	39.18			4', 4''	2, 3, 6', 6''	
H'	2.37	1.94, 2.19	6.70(3), -15.00(4'')	3, 4'', 5	4	2, 3, 5	2, 5
H''	2.33	1.94, 2.19	7.30(3), -15.00(4'), 3.20(5)	3, 4', 5	4	2, 3, 5, 6	2, 5
5 C	73.99	74.64			5	3, 4', 4'', 6'	
H	3.34	4.08	3.20(4'')	4', 4'', 6', 6''	5		2, 4', 4'', 6''
6 C	37.49	39.19			6', 6''	4'', 8', 8''	
H'	1.19	1.54, 1.79	-12.20(6''), 11.30(?), 11.30(?)	5, 6'', 7	6	4, 5, 7	6''
H''	1.99	1.54, 1.79	-12.20(6')	5, 6', 7	6	4, 7, 8	5, 6', 7, 51
7 C	72.69	74.66			7	6', 6'', 8', 8'', 51	
H	3.78	3.93		6', 6'', 8', 8''	7	51	6'', 8'', 51, 54, 55
8 C	39.33	39.7			8', 8''	6''	

H'	1.26	1.54, 1.79	-12.40(8'')	7, 8'', 9	8	6, 7, 9, 10	8'', 9
H''	1.86	1.54, 1.79	-12.40(8')	7, 8', 9	8	6, 7, 9	7, 8', 9, 55
9 C	73.25	74.42			9	8', 8'', 10'	
H	3.34	4.03	4.80(10')	8', 8'', 10', 10''	9		8', 8'', 11, 25
10 C	42.78	44.05			10', 10''	8', 11, 12, 25	
H'	1.64	1.25, 1.50	4.80(9), -14.00(10''), 10.30(11)	9, 10'', 11	10	9, 11, 25	10'', 12
H''	1.25	1.25, 1.50	-14.00(10')	9, 10', 11	10	11, 25	10', 25
11 C	32.73	34.63			11	10', 10'', 12, 13, 25	
H	2.52	2.18	6.52(12), 10.30(10')	10', 10'', 12, 25	11	10, 12, 13	2, 9, 12, 13, 25
12 C	141.98	139.83			12	11, 14, 25	
H	5.73	5.61	6.52(11), 15.30(13)	11, 13	12	10, 11, 14, 25	10', 11, 14, 25
13 C	123.07	126.51			13	11, 14, 15	
H	6.35	6.03	10.80(14), 15.30(12)	12, 14	13	11, 14, 15	11, 16, 21, 25
14 C	130.94	130.7			14	12, 13, 16	
H	6.08	5.97	10.80(13), 10.90(15)	13, 15	14	12, 13, 16	12, 15
15 C	126.39	128.5			15	13, 16, 17	
H	5.36	5.47	10.90(14), 8.00(16)	14, 16	15	13, 16	14, 16, 17, 26
16 C	30.42	31.49			16	14, 15, 18	
H2	2.29	1.94, 2.19	8.00(15)	15, 17	16	14, 15, 18, 20	13, 15, 17, 21, 26
17 C	80.8	87.14			17	26	
H	4.06	4.46	7.00(18)	16, 18	17	15, 19, 20	15, 16, 18, 20, 26
18 C	36.89	38.4			18	16, 19', 19'', 26	
H	2.44	1.85	7.00(17), 7.30(19'), 10.80(19''), 7.00(26)	17, 19', 19'', 26	18	16, 19, 20	17, 19', 20, 26
19 C	35.56	35.4			19', 19''	17, 18, 26	
H'	1.94	1.61, 1.86	-12.15(19''), 6.10(20), 7.30(18)	18, 19'', 20	19	18, 20, 26	18, 19'', 20
H''	1.47	1.61, 1.86	-12.15(19'), 10.80(18), 9.60(?)	18, 19', 20	19	18, 20, 21, 26	19', 20, 21, 26
20 C	81.45	80.58			20	16, 17, 18, 19', 19'', 22', 22''	
H	3.76	3.86	6.10(19')	19', 19'', 21	20		17, 18, 19', 19'', 22', 22''
21 C	71.43	71.34			21	19'', 22', 22''	
H	3.68	3.6		20, 22', 22''	21		13, 16, 19'', 22', 22'', 23, 24a, 24b
21OH O							
H		3.28					
22 C	37.19	37.8			22', 22''	24a, 24b	
H'	1.59	1.51, 1.76	14.40(22'')	21, 22'', 23	22	20, 21, 24	20, 21, 22'', 23, 24a
H''	1.7	1.51, 1.76	14.40(22'), 8.60(?), 3.50(?)	21, 22', 23	22	20, 21, 24	20, 21, 22', 23, 24a
23 C	68.1	72.91			23	24a, 24b	
H	4.09	4.57		22', 22''	23	1	21, 22', 22''
24 C	67.95	66.11			24a	22', 22''	
Ha	4.14	3.77, 4.03			24	22, 23	21, 22', 22''

Hb	4.14	3.77, 4.03				22, 23	21
24OH O	4.14						
H		3.36					
25 C	17.99	21.27			25	10', 10'', 12	
H3	1.01	0.96		11	25	10, 11, 12	9, 10'', 11, 12, 13
26 C	14.49	18.13				19', 19''	
H3	1.04	0.93	7.00(18)	18		17, 18, 19	15, 16, 17, 18, 19''
51 C	93.96	95.46				7	
H	5.01	5.19	1.70(52)			7, 52, 53, 55	6'', 7, 52, 57
52 C	80.6	80.9			52	51, 57	
H	3.39	3.27	1.70(51), 3.80(53)	53	52	53, 54, 57	51, 53
53 C	71.15	71.71			53	51, 52, 54, 55	
H	3.68	3.49	3.80(52)	52, 54	53	54	52
53OH O							
H		2.56					
54 C	74.2	73.3			54	52, 53, 55, 56	
H	3.33	3.25	9.40(55)	53, 55	54	53, 55, 56	7
54OH O							
H		4.29					
55 C	67.93	69.91			55	51, 54, 56	
H	3.62	3.62	9.40(54), 6.30(56)	54, 56	55	53, 54	7, 8'', 56
56 C	17.47	18.32			56	54	
H3	1.26	1.28	6.30(55)	55	56	54, 55	55
57 C	58.89	59.1			57	52	
H3	3.45	3.43			57	52	51

Dataset for **2*p*** is available in NMReDATA[1] format here:

https://dx.doi.org/10.17617/3.34

1.2.2 Full NMR Assignment of 11-epi-2p

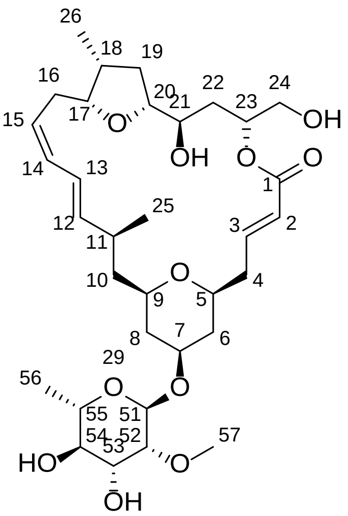

About 1mg of 11epi-**2*p*** was dissolved in about 60uL of CDCl3 and transferred to a 3mm NMR tube. Measurements were performed on a AV 600 equipped with a TCI probehead at 298K.

^{1}H NMR (600 MHz, Chloroform-*d*) δ 7.11 (ddd, *J* = 15.2, 8.2, 6.7 Hz, 1H, 3), 6.11 (dd, *J* = 14.8, 11.0 Hz, 1H, 13), 6.02 (t, *J* = 10.9 Hz, 1H, 14), 5.94 (d, *J* = 15.7 Hz, 1H, 2), 5.34 (dd, *J* = 14.8, 9.6 Hz, 1H, 12), 5.25 (dtd, *J* = 11.6, 5.2, 3.0, 2.3 Hz, 1H, 23), 5.22 (td, *J* = 10.9, 9.6, 5.8 Hz, 1H, 15), 5.01 (d, *J* = 0.8 Hz, 1H, 51), 4.01 (q, *J* = 7.3, 6.2 Hz, 1H, 17), 3.81 (td, *J* = 5.0, 3.3, -12.0 Hz, 1H, 24b), 3.81 – 3.75 (m, 1H, 7), 3.77 (d, *J* = 7.0 Hz, 1H, 20), 3.69 (td, *J* = 9.8, 3.8 Hz, 1H, 53), 3.67 (dt, *J* = 12.0, 6.0, 5.0 Hz, 1H, 24a), 3.63 (dq, *J* = 9.4, 6.2 Hz, 1H, 55), 3.48 (tt, *J* = 9.1, 8.0, 2.8, 2.0 Hz, 1H, 21), 3.40 (dd, *J* = 3.8, 1.4 Hz, 1H, 52), 3.35 (td, *J* = 9.4, 1.8 Hz, 1H, 54), 3.28 (tt, *J* = 11.0, 2.4 Hz, 1H, 5), 3.18 (tt, *J* = 11.1, 1.6 Hz, 1H, 9), 2.75 (s, 1H, 21OH), 2.55 – 2.48 (m, 1H, 11), 2.48 – 2.43 (m, 1H, 54OH), 2.43 – 2.36 (m, 3H, 4', 24OH, 53OH), 2.33 (ddd, *J* = 14.4, 8.2, 2.1 Hz, 1H, 4''), 2.27 (dtd, *J* = 15.0, 8.9, 7.6, 1.3 Hz, 1H, 16'), 2.11 (dt, *J* = 12.3, 7.1 Hz, 1H, 19'), 2.09 (dtd, *J* = 15.0, 6.0, 1.8 Hz, 1H, 16''), 2.00 (dq, *J* = 12.3, 4.6, 2.0 Hz, 1H, 6''), 1.90 (t, *J* = 14.0, 11.7 Hz, 1H, 22b), 1.77 (dq, *J* = 12.4, 4.7, 1.6 Hz, 1H, 8''), 1.57 (ddd, *J* = 14.6, 9.1, 2.1 Hz, 1H, 22a), 1.53 (td, *J* = 13.7, 11.0, 2.7 Hz, 1H, 10'), 1.28 (d, *J* = 84.3 Hz, 4H), 1.28 (d, *J* = 6.2 Hz, 3H, 56), 1.17 (td, *J* = 11.5, -12.3 Hz, 1H, 6'), 1.16 (ddd, *J* = 11.2, 1.3, -13.7 Hz, 1H, 10''), 1.00 (dd, *J* = 6.9, 2.1 Hz, 3H, 25, 26).

^{13}C NMR (151 MHz, Chloroform-*d*) δ 166.51 , (1), 145.90 , (3), 141.09 , (12), 130.39 , (14), 125.94 , (15), 124.66 , (13), 123.39 , (2), 93.89 ,(51), 81.90 , (20), 81.53 , (17), 80.62 , (52), 73.95 , (54), 73.71 , (5), 73.67 , (23), 73.07 , (21), 72.99 , (9), 72.46 , (7), 71.41 , (53), 67.93 , (55), 65.51 , (24), 58.91 , (57), 43.27 , (10), 39.23 , (4), 39.01 , (8), 37.98 , (6), 36.65 , (18), 36.20 , (19), 34.42 , (22), 33.86 , (11), 30.81 , (16), 21.77 , (25), 17.49 , (56), 14.63 , (26).

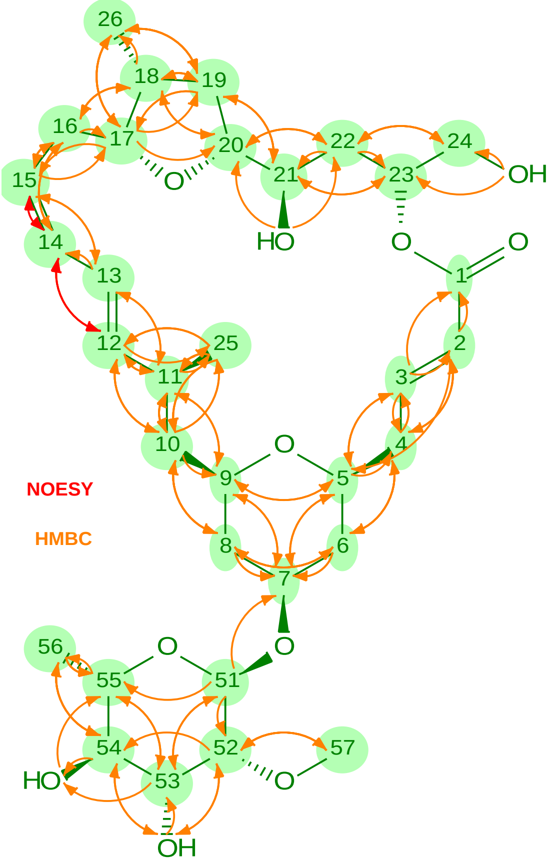

Summary of correlations for 11-*epi*-**2p**

Table S8: Full NMR assignment of 11-*epi*-**2p**

Assignments						
Atom	Chemical Shift --ppm	Predicted Shift --ppm	J in Hz (coupling partner)	COSY	HSQC	HMBC
1 C	166.51	166.98				3, 2
2 C	123.39	122.4			2	3, 4', 4''
H	5.94	5.9	15.60(3)	3	2	1, 4, 5

3 C	145.9	141.02			3	5, 4', 4''
H	7.1	6.97	15.60(2), 8.20(4''), 6.70(4')	2, 4'', 4'	3	2, 5, 1, 4
4 C	39.23	39.18			4', 4''	3, 6', 6'', 5, 2
H'	2.41	2.13, 2.35	6.70(3), 14.40(4''), 10.90(5)	3, 4''	4	2, 6, 3
H''	2.33	2.13, 2.35	8.20(3), 14.40(4'), 2.30(5)	3, 4'	4	2, 6, 3
5 C	73.71	74.64			5	3, 7, 9, 2
H	3.28	3.82	10.90(4'), 2.30(4''), 2.40(6''), 11.00(6')	5, 6'', 6'	5	9, 4, 3, 7
6 C	37.98	39.19			6', 6''	4', 4'', 8', 8''
H'	1.17	1.50, 1.78	11.00(5), -12.50(6''), 10.00(7)	5, 7, 6''	6	8, 4, 7
H''	2.01	1.50, 1.78	2.40(5), 4.60(7), -12.50(6')	5, 7, 8'', 6'	6	8, 4, 7
7 C	72.46	74.66			7	5, 9, 51, 6', 6'', 8', 8''
H	3.77	3.99	4.60(6''), 10.00(6')	8'', 6'', 6', 8'	7	5, 9
8 C	39.01	39.7			8', 8''	6', 6'', 10'', 10'
H'	1.28	1.54, 1.76	-12.40(8''), 11.10(9)	7, 9, 8''	8	6, 10, 7
H''	1.77	1.54, 1.76	-12.40(8'), 1.60(9)	7, 6'', 9, 8'	8	6, 10, 7
9 C	72.99	74.42			9	5, 7, 10', 11
H	3.18	3.84	11.10(8'), 1.60(8''), 11.10(10'), 1.50(10'')	8'', 10'', 8', 10'	9	5, 7, 11
10 C	43.27	44.05			10', 10''	8', 8'', 12, 11, 25
H'	1.54	1.22, 1.67	-13.70(10''), 2.70(11), 11.10(9)	9, 11, 10''	10	8, 25, 12, 9, 11
H''	1.16	1.22, 1.67	-13.70(10'), 1.50(9)	9, 11, 10'	10	8, 25, 12, 11
11 C	33.86	34.63			11	9, 12, 10', 10'', 25, 13
H	2.5	2.08	9.60(12), 6.80(25), 2.70(10')	10'', 12, 25, 10'	11	9, 25, 13, 12, 10
12 C	141.09	139.83			12	10'', 14, 10', 11, 25
H	5.34	5.39	14.80(13), 9.60(11)	11, 13	12	25, 10, 14, 11
13 C	124.66	126.51			13	15, 11
H	6.12	5.73	14.80(12), 11.00(14)	12, 14	13	15, 11, 14
14 C	130.39	130.7			14	12, 16'', 16', 13
H	6.02	5.91	11.00(13), 10.90(15), 1.80(16''), 1.20(16')	13, 15	14	16, 12
15 C	125.94	128.5			15	17, 16'', 16', 13
H	5.21	5.44	10.90(14), 5.90(16''), 9.60(?)	14, 16'', 16'	15	13, 17, 16
16 C	30.81	31.49			16', 16''	14, 18, 15
H'	2.27	2.15, 2.42	1.20(14), -14.70(16''), 7.60(17), 8.90(?)	15, 17, 16''	16	14, 15, 17, 18
H''	2.09	2.15, 2.42	1.80(14), 5.90(15), 5.90(17), -14.70(16')	15, 17, 16'	16	14, 15, 18
17 C	81.53	87.14			17	15, 19', 16', 26, 19''
H	4.01	4.35	7.30(18), 5.90(16''), 7.60(16')	16'', 18, 16'	17	26, 19, 20, 15
18 C	36.65	38.4			18	16'', 20, 16', 19', 19''
H	2.48	1.82	7.30(17), 7.00(26)	17, 26, 19', 19''	18	26, 19, 16, 20
19 C	36.2	35.4			19', 19''	17, 21, 18, 26
H'	2.11	1.41, 1.96		19'', 18, 20	19	26, 17, 21, 18
H''	1.29	1.41, 1.96		19', 18, 20	19	17, 26, 21, 18
20 C	81.9	80.58			20	18, 21OH, 22a, 22b, 17
H	3.77	3.83	8.00(21)	19', 19'', 21	20	22, 18
21 C	73.07	71.34			21	19', 23, 22a, 19''
H	3.48	3.39	8.00(20), 9.10(22a), 2.00(22b), 2.80(?)	20, 21OH, 22a, 22b	21	19, 23

H	2.75	2.61		21		22, 20
22 C	34.42	37.8			22a, 22b	20, 21OH, 24a
Ha	1.57	1.74, 1.98	-14.40(22b), 2.00(23), 9.10(21)	21, 23, 22b	22	24, 20, 21
Hb	1.9	1.74, 1.98	-14.40(22a), 11.60(23), 2.00(21)	22a, 21, 23	22	24, 20, 23
23 C	73.67	72.91			23	21, 24OH, 22b
H	5.25	4.84	2.00(22a), 11.60(22b), 3.00(?), 5.20(?)	22a, 22b, 24a, 24b	23	21
24 C	65.51	66.11			24a, 24b	22a, 22b, 24OH
Ha	3.67	3.68, 4.05		23, 24OH, 24b	24	22
Hb	3.81	3.68, 4.05		24a, 23, 24OH	24	
24OH O						
H	2.41	2.61		24a, 24b		24, 23
25 C	21.77	21.27			25	10'', 12, 10', 11
H3	1	0.99	6.80(11)	11	25	12, 10, 11
26 C	14.63	18.13			26	17, 18, 19', 19''
H3	1	0.95	7.00(18)	18	26	17, 19
51 C	93.89	95.46			51	53
H	5.01	5.32		52	51	53, 55, 52, 7
52 C	80.62	80.9			52	57, 51, 53OH
H	3.4	3.61	3.80(53)	51, 53	52	57, 54, 53OH
53 C	71.41	71.71			53	55, 51, 53OH
H	3.7	3.61	3.80(52), 9.80(54), 9.80(53OH)	52, 53OH, 54	53	51, 54OH, 55
53OH O						52, 54
H	2.41	2.22	9.80(53)	53		54, 52, 53
54 C	73.95	73.3			54	52, 56, 53OH
H	3.35	3.47	9.80(53)	53, 55, 54OH	54	53OH, 54OH, 56
H	2.47	4.29		54		55
55 C	67.93	69.91			55	53, 56, 51, 54OH
H	3.63	3.5		54, 56	55	53, 56
56 C	17.49	18.32			56	55, 54
H3	1.28	1.28		55	56	54, 55
57 C	58.91	59.1			57	52
H3	3.46	3.43			57	52

Dataset for 11-epi-**2*p*** is available in NMReDATA[1] format here: https://dx.doi.org/10.17617/3.34

2 Methods

2.1 Calculation of Dipolar Couplings

The dipolar coupling constant *D* can be calculated from the distance *R* of the magnetic nuclei *A* and *B*:

$$D_{AB} = \frac{\hbar\mu_0}{4\pi}\frac{\gamma_A\gamma_B}{R_{AB}^3} \tag{S1}$$

The maximum dipolar splitting is *2D* for the week coupling case and *3D* for the strong coupling case. In the principal axes system with the z-axis in the direction from atom A to atom B the dipolar coupling tensor $\mathbf{D}_{AB}$ has the following form:

$$\mathbf{D}_{AB} = \begin{pmatrix} -D_{AB}/2 & 0 & 0 \\ 0 & -D_{AB}/2 & 0 \\ 0 & 0 & D_{AB} \end{pmatrix} \quad \text{(S2)}$$

For the bond dipolar couplings the following fixed coupling constants are used:

Table S9: Coupling constants for 13C1H one bond dipolar couplings

Bond Type	Average Distance* / Å	Coupling Constant D / Hz
C(sp3)-H	1.093	23136.65
C(sp2)-H	1.080	23982.25

* The average distances are obtained from J. Chem. Soc. Perkin Trans. JI 1987

2.2 The Recursion Formula for the Time Average with an Exponential Decaying MemoryFunction

The time average of all components the dipolar tensor **D** is calculated using the following exponentially decaying memory function:

$$\mathbf{D}(t) = \frac{1}{N(t)} \int_{t'=t_0}^{t} e^{\frac{-(t-t')}{\tau}} \mathbf{D}(t') \quad dt'$$
$$N(t) = \int_{t'=t_0}^{t} e^{\frac{-(t-t')}{\tau}} \quad dt' \quad \text{(S3)}$$

The second integral N(t) is the norm and τ the memory time constant. This folding integral transforms the original function **D**(t') to the new time scale t. Introducing n equidistant discrete time steps Δt with t' ={0,...,n Δt} the folding integral (S3) can be written in matrix form as follows:

$$\begin{pmatrix} \mathbf{d}_{t_0} \\ \mathbf{d}_{t_1} \\ \vdots \\ \mathbf{d}_{t_n} \end{pmatrix} = \begin{pmatrix} e^{-\frac{0}{\tau}} & 0 & \cdots & 0 \\ e^{-\frac{\Delta t}{\tau}} & e^{-\frac{0}{\tau}} & \cdots & 0 \\ \vdots & \vdots & \vdots & \vdots \\ e^{-\frac{n\Delta t}{\tau}} & e^{-\frac{(n-1)\Delta t}{\tau}} & \cdots & e^{-\frac{0}{\tau}} \end{pmatrix} \begin{pmatrix} \mathbf{D}_{t'_0} \\ \mathbf{D}_{t'_1} \\ \vdots \\ \mathbf{D}_{t'_n} \end{pmatrix} \quad \text{(S4)}$$

To obtain the final $< D >_{k\Delta t}$ values ($<>_t$ indicates the time average) are obtained by the elements of the column on the right side have to be divided by the norm:

$$\langle \mathbf{D} \rangle_{k\Delta t} = \frac{\mathbf{d}_k}{N_k} \tag{S5}$$

The vector of the norm values can be obtained by replacing the column vector on the right side of equation (S4)with a vector containing only *1* as elements. From (S4) we can write down the following recursion for **d** and *N*:

$$\begin{aligned} &\mathbf{d}_0 = \mathbf{D}_0 \\ &\mathbf{d}_{k+1} = \mathbf{d}_i e^{-\frac{\Delta t}{\tau}} + \mathbf{D}_{k+1} \\ &N_0 = 1 \\ &N_{k+1} = N_k e^{-\frac{\Delta t}{\tau}} + 1 \end{aligned} \tag{S6}$$

In this version the norm N_i has to be stored together with $\mathbf{d_k}$. Depending on resources and the speed of memory access it may be favorable to run the recursion directly using $< D >_{k\Delta t}$:

$$\begin{aligned} &\langle \mathbf{D} \rangle_{t=0} = \mathbf{D}_0 \\ &N_0 = 1 \\ &N_{k+1} = N_k e^{-\frac{\Delta t}{\tau}} + 1 \\ &\langle \mathbf{D} \rangle_{(k+1)\Delta t} = \frac{1}{N_{k+1}} \left(\mathbf{D}_{k+1} + N_k \langle \mathbf{D} \rangle_{k\Delta t} e^{-\frac{\Delta t}{\tau}} \right) \end{aligned} \tag{S7}$$

The sum within the parenthesis is calculated before the recursion of the norm is executed.

2.3 Performing MDOC Calculations

All calculations performed in this paper are performed using the COSMOS (Computer Simulations of MOlecular Structures) software. For MDOC simulations at first a coordinate (*.coo) file is necessary. This file can be created by Molecular Modelling using the program COSMOS-Frontend or the atomic coordinates can be imported from data collections as for instance from PDB (Protein Data Bank), from CSD (Cambridge Structural Database) or from other modelling programs. The COSMOS-Frontend runs a Windows graphical interface (GUI) and imports 20 data types.

The COSMOS coordinate files can store additionally to the coordinates experimental NMR data and the results of calculations. The experimental data are assigned to the atoms using their site names that are present in coordinate file as well. From the NMR measurements a COSMOS data file (*.cod) is prepared and parsed into the *.coo file using the COSMOS programs.

Time consuming calculations are mostly performed on HPC systems using the COSMOS-Backend program[1]. The execution is controlled by a COSMOS project (*.cos) file. This file can be stored by the COSMOS-Frontend but it can be also edited using any ASCII editor. The entries of the project file controlling MDOC calculations are given in chapter 1.6.

During MDOC simulations the COSMOS-Backend can store snapshots of coordinate files that contain calculated NMR properties as for instance RDC values. These stored RDC are values that are calculated with the exponential memory function (see Equations (S3)-(S7)). The COSMOS-Backend can be used to perform the final data averages over the time dependent values. The COSMOS-Backend can additionally store data snapshots that contain RDC values or geometry data like distances and torsion angles. The data snapshots are saved in Mathematica format and notebooks have been developed to perform several types of analysis.

2.4 *Analysis of MDOC Trajectories*

The analysis of MDOC torsion trajectories is performed using Mathematica notebooks. In relatively simple cases like the 1,4-diketone it is sufficient to look at on the distribution of dihedral angles of only a few bonds.

In most cases as for instance for Mandelalide A the dihedrals are regularly not independent but appear only in certain combinations. The reason for that is that some molecules with a definite combination of dihedrals form islands of stability within the dihedral landscape. For proteins these islands of stability are the well-known helices and beta sheets. If we would perform an analysis of the occurrence of all combinations of dihedrals for only a moderate number of bonds we would reach a large number of such combinations even if we would restrict on three bins like {trans, gauche(-), gauche(+)}.

A first assumption is to treat the dihedral independently and to search simply for molecular models within our snapshot library for exemplars that display angles near the maxima of {Tr, G-, G+}. In our Mandelalide example this was performed for the 2p (10 models) and 11-epi-2p structures (12 models) and the models with the lowest force field energies are taken for the Figures 10 and 11 of the paper.

An approach that works without the above assumptions is the Principal Components Analysis (PCA) on the basis of dihedral angles as developed by Altis *et al.*[2] In a first step one defines unique internal coordinates from our dihedral angles φ_n:

$$q_n = \cos(\varphi_n) + i \sin(\varphi_n) \tag{S8}$$

It is convenient to concentrate on N characteristic backbone dihedrals that display pronounced variability. The idea behind that is to concentrate on big, long lasting changes and to exclude

[1] The COSMOS Backend program is provided without charge by one of the authors (Ulrich Sternberg). A description of all file types, a manual and examples are provided as well. Please contact ulrich.sternberg@partner.kit.edu and visit www.cosmos-software.de.

rapid motions like vibrations from the consideration. The correlated internal motions can be described using a covariance matrix:

$$\sigma_{ij} = \left\langle \left(q_i - \langle q_i \rangle\right)\left(q_j - \langle q_j \rangle\right) \right\rangle \tag{S9}$$

The complex covariance matrix (S9) has N real eigenvalues. The eigenvectors $v^{(i)}$ describe the modes of collective motion and the largest eigenvalues belong to eigenvectors or principal components with the highest variability.

The components of the eigenvectors can be analysed for the contribution of a certain dihedrals q_i by calculating the absolute value or modulus (index m) of the components $v^{(i)}$. In this way we can find out which dihedrals contribute most to a certain principal component (see Figure 12 of the paper). In this way we can look for molecular models that are governed by a certain combination of dihedrals. The principal components of the data V_i are calculated from the internal coordinates q (see Equation (S8), represented as column vector):

$$V_i = \mathbf{v}_m^{(i)} \mathbf{q} \tag{S10}$$

In this paper only the two eigenvectors $v^{(1)}$ and $v^{(2)}$ are analyzed that belong to the largest eigenvalues of the covariance matrix. The V_i can be used to represent dihedral distributions or free energy surfaces of the system[3].

2.5 Parameters for the MDOC simulation

Table S10: General parameters for the MDOC simulations

Parameter	*Value*
Target temperature	*300 K*
MD time step	*0.5 fs*
BPT atomic charge calculation	*2 fs*
Coupling time η to the heat bath	*0.02 ps*
Memory decay time τ for the property average	*200 ps*
Time constant ρ for the exponential rise of pseudo-forces	*200 ps*
Order parameter of the alignment medium S_{am} (dipolar couplings)	*0.004*
Order parameter of the alignment medium S_{am} (dipolar splittings)	*0.008*
*Enhancement factor for the torsion of π-bonds**	*1.5*

** The torsion barrier for π-bonds with valence 2.0 is stepped up from 94.403 kJ/mol to 141.6045 kJ/mol. This enhances the stiffness of π-systems and prevents too large distortions by orientational pseudo-forces.*

Table S11: Width and weight parameters for the pseudo forces according equations (4) and (5) of the paper.

1, 4 Diketone simulations	
Parameter	Value
Pseudo-force width *ΔD* for the one bond CH couplings	0.5 Hz
Weight parameter k_D (one bond - in kJ mol^{-1} Hz^{-1})	0.00025
Pseudo-force width *ΔD* for the one long range couplings	0.5 Hz
Weight parameter k_D (long range - in kJ mol^{-1} Hz^{-1})	0.002
Pseudo-force width *ΔR* for the NOE distance constraints	0.4 Å
Weight parameter k_R (NOE distances - in kJ mol^{-1} Å^{-1})	0.3
Pseudo-force width *ΔJ* for the $^3J_{HH}$ coupling constraints	1.0 Hz
Weight parameter k_J ($^3J_{HH}$ couplings - in kJ mol^{-1} Hz^{-1})	4.0
Total MD duration	80 ns
Mandelalide A simulations	
Parameter	Value
Pseudo-force width *ΔD* for the one bond CH couplings	0.5 Hz
Weight parameter k_D (one bond - in kJ mol^{-1} Hz^{-1})	0.00025
Pseudo-force width *ΔR* for the NOE distance constraints	1.0 Å
Weight parameter k_R (NOE distances - in kJ mol^{-1} Å^{-1})	0.25
Pseudo-force width *ΔJ* for the $^3J_{HH}$ coupling constraints	2.0 Hz
Weight parameter k_J ($^3J_{HH}$ couplings - in kJ mol^{-1} Hz^{-1})	10
Total MD duration	41.2 ns

3 MDOC Results

3.1 Results of 1,4-Diketone MDOC Simulations

Table S12: Experimental and calculated RDC splittings of **1-*SS*** and **1-*SR*** with data set 1A (outliers in red).

1A: RDC-CH									
expDATA				1SS			1SR		
Atom A	Atom B	RDC Exp. / Hz	Error / Hz	RDC MDOC / Hz	Diff. Exp.-Calc. / Hz	χ2	RDC MDOC / Hz	Diff. Exp.-Calc. / Hz	χ2
H6	C6	-9.80	1.20	-9.76	-0.04	0.00	-9.70	-0.10	0.01
H5	C5	13.50	1.60	13.73	-0.23	0.02	13.78	-0.28	0.03
H4	C4	21.50	1.40	21.40	0.10	0.01	21.24	0.26	0.04
H3	C3	-11.60	1.60	-11.63	0.03	0.00	-11.66	0.06	0.00
H8	C8	-12.00	0.90	-11.85	-0.15	0.03	-11.66	-0.34	0.14
H9	C9	21.50	0.50	21.33	0.17	0.11	21.12	0.38	0.57
H13b	C13	-11.10	1.00	-11.28	0.18	0.03	-11.24	0.14	0.02
H13a	C13	2.80	0.60	2.29	0.51	0.71	2.25	0.55	0.84
H12	C12	-15.00	0.50	-14.18	-0.83	2.72	-13.90	-1.10	4.81
H11c	C11	3.10	1.60	2.75	0.35	0.05	2.66	0.44	0.08
H11a	C11	3.10	1.60	2.81	0.29	0.03	2.69	0.41	0.07
H11b	C11	3.10	1.60	2.77	0.33	0.04	2.68	0.42	0.07
H15	C15	6.90	1.10	6.96	-0.06	0.00	7.01	-0.11	0.01
H16	C16	-5.80	0.60	-5.66	-0.14	0.06	-5.58	-0.23	0.14
H17	C17	19.70	4.00	19.77	-0.07	0.00	19.58	0.12	0.00
H18	C18	7.10	0.70	6.95	0.15	0.05	6.89	0.21	0.09
					n	16		n	16
					outliers	1		outliers	1
					F	15/16		F	15/16
					min(1/X2)	0.37		min(1/X2)	0.21
					n/X2	4.14		n/X2	2.31

1A: RDC-HH									
expDATA				1SS			1SR		
Atom A	Atom B	RDC Exp. / Hz	Error / Hz	RDC MDOC / Hz	Diff. Exp.-Calc. / Hz	χ2	RDC MDOC / Hz	Diff. Exp.-Calc. / Hz	χ2
H6	H8	10.20	0.90	8.77	1.43	2.51	8.37	1.83	4.12
H5	H6	8.10	0.40	7.49	0.61	2.30	7.25	0.85	4.47
H3	H4	4.80	0.40	4.74	0.06	0.02	4.82	-0.02	0.00
H8	H9	5.60	0.40	5.18	0.42	1.12	5.20	0.40	1.02
H13a	H13b	-14.40	0.50	-13.92	-0.48	0.91	-14.06	-0.34	0.47
H15	H16	8.80	0.40	8.06	0.74	3.45	7.61	1.19	8.87
H16	H17	2.10	0.40	2.03	0.08	0.04	2.03	0.07	0.03
H18	H17	-2.40	0.40	-2.23	-0.17	0.19	-2.07	-0.33	0.69
					n	8		n	8
					outliers	4		outliers	4
					F	4/8		F	4/8
					min(1/X2)	0.29		min(1/X2)	0.11
					n/X2	0.76		n/X2	0.41

Total RDC (1SS)		Total RDC (1SR)	
n	24	n	24
outliers	5	outliers	5
F	19/24	F	19/24
min(1/X2)	0.29	min(1/X2)	0.11
n/X2	1.67	n/X2	0.90

RMS deviation: 0.45 Hz

Table S13: Experimental and calculated NOE distances of **1-*SS*** and ***1-SR*** with data set 1A.

1A: NOE									
expDATA				1SS			1SR		
Atom A	Atom B	NOE Dist. Exp. / Å	Error / Å	NOE Dist. MDOC / Å	Diff. Exp.-Calc. / Å	χ2	NOE Dist. MDOC / Å	Diff. Exp.-Calc. / Å	χ2
H8	H13b	3.90	0.30	3.99	-0.09	0.08	3.96	-0.06	0.04
H9	H13a	4.50	0.30	3.98	0.52	3.04	3.47	1.03	11.83
H9	H13b	3.20	0.30	3.14	0.06	0.04	2.82	0.38	1.60
H9	H12	3.30	0.30	3.09	0.21	0.48	2.85	0.45	2.27
H13a	H11c	4.20	0.40	4.20	0.00	0.00	4.06	0.14	0.12
H13a	H11a	4.20	0.40	4.20	0.00	0.00	4.08	0.12	0.09
H13a	H11b	4.20	0.40	4.20	0.00	0.00	4.06	0.14	0.12
H13b	H11c	3.60	0.40	3.68	-0.08	0.04	3.45	0.15	0.14
H13b	H11a	3.60	0.40	3.69	-0.09	0.05	3.48	0.13	0.10
H13b	H11b	3.60	0.40	3.69	-0.09	0.05	3.45	0.15	0.14
H12	H11c	2.60	0.40	2.89	-0.29	0.53	2.94	-0.34	0.70
H12	H11a	2.60	0.40	2.89	-0.29	0.52	2.94	-0.34	0.72
H12	H11b	2.60	0.40	2.89	-0.29	0.51	2.93	-0.33	0.66
					**n**	**13**		**n**	**13**
					**outliers**	**1**		**outliers**	**3**
					**F**	**12/13**		**F**	**10/13**
					**min(1/X2)**	**0.33**		**min(1/X2)**	**0.08**
					**n/X2**	**2.44**		**n/X2**	**0.70**

RMS deviation: 0.21 Å

Table S14: Experimental and calculated ^{3}J couplings of **1-*SS*** and ***1-SR*** with data set 1A.

1A: J-couplings									
expDATA				1SS			1SR		
Atom A	Atom B	3J Coup. Exp. / Hz	Error[3)] / Hz	3J Coupling MDOC / Hz	Diff. Calc.-Exp. / Hz	χ2	3J Coupling MDOC / Hz	Diff. Calc.-Exp. / Hz	χ2
C11	H12	3.20	1.06	3.53[1)]	0.33	0.10	3.49	0.29	0.08
H13a	H12	7.90	0.75	7.53[2)]	-0.37	0.24	7.57	-0.33	0.19
H13b	H12	4.60	0.75	5.23[2)]	0.63	0.70	5.20	0.60	0.63
					**n**	**3**		**n**	**3**
					**outliers**	**0**		**outliers**	**0**
					**F**	**3/3**		**F**	**3/3**
					**min(1/X2)**	**1.43**		**min(1/X2)**	**1.59**
					**n/X2**	**2.89**		**n/X2**	**3.34**

1) Calculated using the method of Palermo et al.[4]

2) Calculated using the method of Haasnoot et al.[5]

3) The error of $^3J_{HH}$ couplings is estimated from the error of the experiment of 0.4 Hz plus the error of the prediction of 0.65 Hz. For the $^3J_{HC}$ couplings the error of the prediction was estimated to 0.662 Hz.

RMS deviation: 0.46 Hz

Table S15: Experimental and calculated RDC splittings of **1-*SR*** and **1-*SS*** with data set 1B.

1B: RDC-CH									
expDATA				1SR			1SS		
Atom A	Atom B	RDC Exp. / Hz	Error / Hz	RDC MDOC / Hz	Diff. Exp.-Calc. / Hz	χ2	RDC MDOC / Hz	Diff. Exp.-Calc. / Hz	χ2
H6	C6	-12.10	0.50	-12.07	-0.03	0.00	-12.02	-0.08	0.02
H5	C5	18.60	1.50	18.64	-0.04	0.00	18.71	-0.11	0.00
H4	C4	12.30	0.70	12.19	0.11	0.02	12.05	0.25	0.13
H3	C3	-12.00	0.20	-12.05	0.05	0.06	-12.09	0.09	0.22
H8	C8	-11.60	0.40	-11.55	-0.05	0.01	-11.41	-0.19	0.22
H9	C9	10.40	0.70	10.52	-0.12	0.03	10.33	0.07	0.01
H13a	C13	-4.10	1.30	-4.17	0.07	0.00	-4.16	0.06	0.00
H13b	C13	-20.30	1.30	-19.96	-0.34	0.07	-19.93	-0.37	0.08
H12	C12	-15.60	0.40	-14.93	-0.67	2.83	-14.54	-1.06	6.97
H11c	C11	3.30	0.20	3.13	0.17	0.71	2.98	0.32	2.54
H11a	C11	3.30	0.20	3.13	0.17	0.73	2.95	0.35	3.06
H11b	C11	3.30	0.20	3.15	0.15	0.56	2.96	0.35	2.98
H15	C15	-0.90	0.90	-0.93	0.03	0.00	-0.98	0.08	0.01
H16	C16	24.40	1.00	24.27	0.13	0.02	24.20	0.20	0.04
H17	C17	3.30	0.60	1.66	1.64	7.47	1.92	1.38	5.28
H18	C18	0.30	0.10	0.32	-0.02	0.03	0.33	-0.03	0.11
					**n**	**16**		**n**	**16**
					**outliers**	**2**		**outliers**	**5**
					**F**	**14/16**		**F**	**11/16**
					**min(1/X2)**	**0.13**		**min(1/X2)**	**0.14**
					**n/X2**	**1.28**		**n/X2**	**0.74**

1B: RDC-HH									
expDATA				1SR			1SS		
Atom A	Atom B	RDC Exp. / Hz	Error / Hz	RDC MDOC / Hz	Diff. Exp.-Calc. / Hz	χ2	RDC MDOC / Hz	Diff. Exp.-Calc. / Hz	χ2
H6	H8	8.60	0.70	7.82	0.79	1.26	7.50	1.10	2.48
H5	H6	4.30	1.20	4.06	0.24	0.04	3.92	0.38	0.10
H4	H5	-4.90	1.20	-4.74	-0.16	0.02	-4.73	-0.17	0.02
H3	H4	6.60	0.70	6.47	0.13	0.03	6.55	0.05	0.00
H8	H9	7.10	0.50	6.63	0.47	0.89	6.59	0.51	1.02
H13a	H13b	-14.00	1.00	-13.92	-0.08	0.01	-13.88	-0.12	0.02
H12	H13a	3.90	0.50	3.98	-0.08	0.03	3.82	0.08	0.02
H15	H16	0.80	0.90	0.73	0.07	0.01	0.87	-0.07	0.01
H16	H17	-0.30	1.00	-0.35	0.05	0.00	-0.36	0.06	0.00
H17	H18	8.60	0.80	8.12	0.48	0.36	8.01	0.59	0.54
H9	H13b	±4.8	1.50	2.336[1)]	2.46	2.70	1.24	3.56	5.63
					**n**	**10**		**n**	**10**
					**outliers**	**1**		**outliers**	**2**
					**F**	**9/10**		**F**	**8/10**
					**min(1/X2)**	**0.80**		**min(1/X2)**	**0.40**
					**n/X2**	**3.78**		**n/X2**	**2.37**

**Total RDC**		**Total RDC**	
**n**	**26**	**n**	**26**
**outliers**	**3**	**outliers**	**7**
**F**	**23/26**	**F**	**19/26**
**min(1/X2)**	**0.13**	**min(1/X2)**	**0.14**
**n/X2**	**1.71**	**n/X2**	**1.00**

[1)] Predicted and not taken as constraint because unknown sign from experiment

Table S16: Experimental and calculated NOE distances of **1-*SR*** and **1-*SS*** with data set 1B.

1B: NOE									
expDATA				1SR			1SS		
Atom A	Atom B	NOE Dist. Exp. / Å	Error / Å	NOE Dist. MDOC / Å	Diff. Exp.-Calc. / Å	χ2	NOE Dist. MDOC / Å	Diff. Exp.-Calc. / Å	χ2
H9	H13b	2.30	0.30	2.49	-0.19	0.41	2.86	-0.56	3.45
H9	H12	3.00	0.30	3.26	-0.26	0.75	2.70	0.30	1.03
H13a	H11c	3.20	0.40	3.36	-0.16	0.16	3.45	-0.25	0.40
H13a	H11a	3.20	0.40	3.37	-0.17	0.17	3.46	-0.26	0.41
H13a	H11b	3.20	0.40	3.36	-0.16	0.16	3.46	-0.26	0.42
H13b	H11c	2.70	0.40	2.85	-0.15	0.15	2.90	-0.20	0.25
H13b	H11a	2.70	0.40	2.85	-0.15	0.14	2.90	-0.20	0.25
H13b	H11b	2.70	0.40	2.85	-0.15	0.14	2.90	-0.20	0.26
H12	H11c	2.70	0.40	2.95	-0.25	0.38	2.98	-0.28	0.49
H12	H11a	2.70	0.40	2.94	-0.24	0.37	2.98	-0.28	0.48
H12	H11b	2.70	0.40	2.95	-0.25	0.39	2.98	-0.28	0.50
					**n**	**11**		**n**	**11**
					**outliers**	**0**		**outliers**	**2**
					**F**	**11/11**		**F**	**9/11**
					**min(1/X2)**	**1.33**		**min(1/X2)**	**0.29**
					**n/X2**	**3.40**		**n/X2**	**1.39**

Table S17: Experimental and calculated ^{3}J couplings of **1-*SR*** and **1-*SS*** with data set 1B.

1B: J-couplings									
expDATA				1SR			1SS		
Atom A	Atom B	3J Coup. Exp. / Hz	Error[3] / Hz	3J Coupling MDOC/ Hz	Diff. Calc.-Exp. / Hz	χ2	3J Coupling MDOC/ Hz	Diff. Calc.-Exp. / Hz	χ2
C11	H12	2.60	1.06	3.16[1]	0.56	0.27	3.78[1]	1.18	1.23
H13a	H12	8.40	0.75	7.93[2]	-0.48	0.40	7.86[2]	-0.54	0.52
H13b	H12	5.10	0.75	5.76[2]	0.66	0.78	6.07[2]	0.97	1.66
					**n**	**3**		**n**	**3**
					**outliers**	**0**		**outliers**	**2**
					**F**	**3/3**		**F**	**1/3**
					**min(1/X2)**	**1.28**		**min(1/X2)**	**0.60**
					**n/X2**	**2.06**		**n/X2**	**0.88**

1) Calculated using the method of Palermo et al.[4]

2) Calculated using the method of Haasnoot et al.[5]

3) The error of $^3J_{HH}$ couplings is estimated from the error of the experiment of 0.4 Hz plus the error of the prediction of 0.65 Hz. For the $^3J_{HC}$ couplings the error of the prediction was estimated to 0.662 Hz.

Table S18: Quality criterion n/χ^2 for the datasets 1A and 1B. The quality data of the configuration assigned to its dataset are displayed in red.

1.4-Diketone Diastereomer	Quality n/χ^2 one bond splittings	Quality n/χ^2 long range splittings	Quality n/χ^2 NOE distances	Quality n/χ^2 ^{3}J couplings	Total Quality n/χ^2
	Dataset 1A				
1-SR	2.31	0.41	0.70	3.34	0.87
1-SS	4.15	0.76	2.44	2.89	1.93
	Dataset 1B				
1-SR	1.28	3.78	3.40	2.05	2.01
1-SS	0.73	2.37	1.38	0.88	1.07

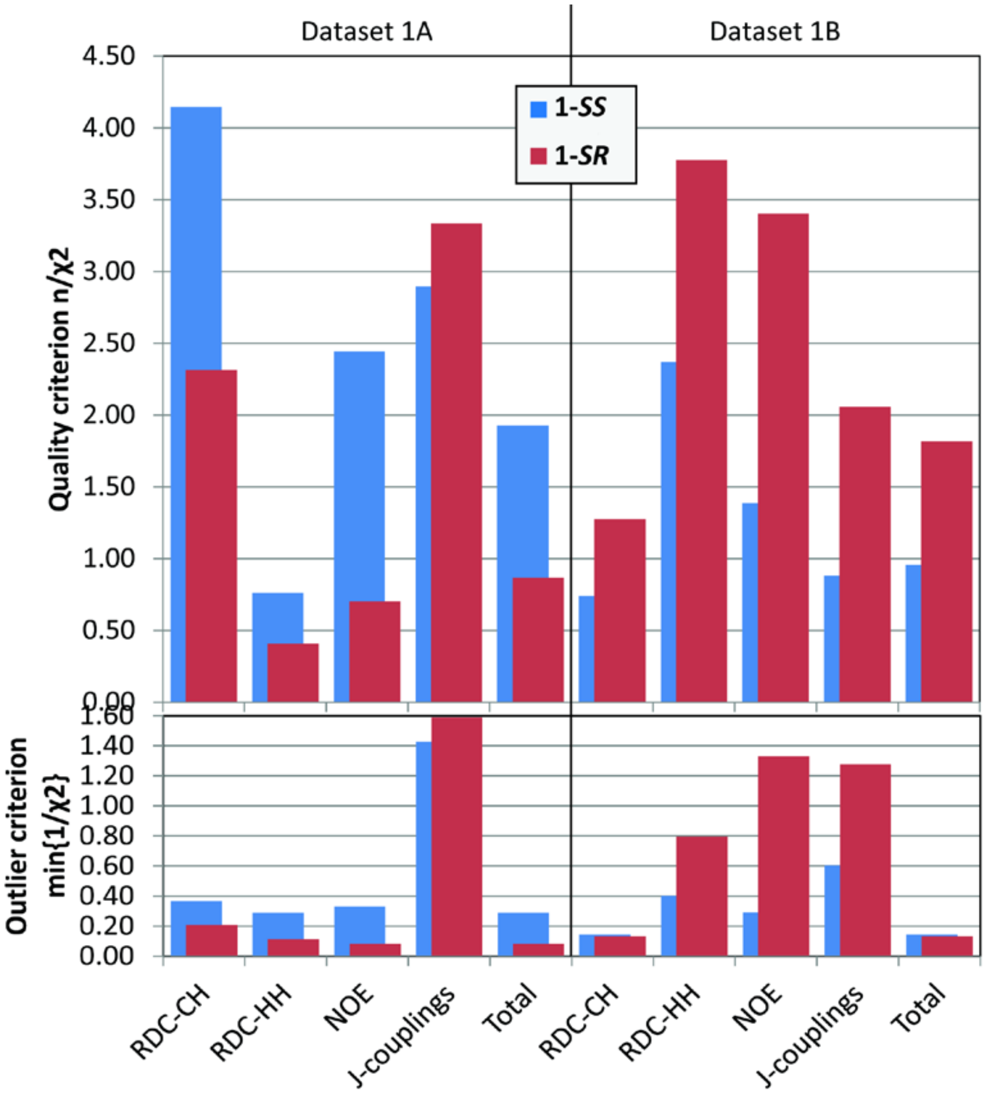

Figure S2: Quality criterion and outlier criterion for dataset 1A and 1B against MDOC simulation on configurations ***1-SS*** and ***1-SR***.

3.2 Results of Mandelalide A MDOC Simulations

Table S19: RMSD values and quality criteria n/χ^2

	RMSD	quality criterion n/χ^2
Mandelalide A Diastereomer **2*p*** – (11*R*)		
Dipolar splittings	0.639 Hz	2.76
NOE distances	0.232 Å	4.64
$^3J_{HH}$ indirect couplings	0.526 Hz	3.61
Total quality criterion n/χ^2		3.95
Total quality n/χ^2 - 11-epi-**2*p*** configuration		3.78
Total quality n/χ^2 - **2*r*** configuration		1.80
Total quality n/χ^2 - 11-epi-**2*r*** configuration		1.78
Mandelalide A Diastereomer 11-epi-**2*p*** – (11*S*)		
Dipolar splittings	0.582 Hz	5.43
NOE distances	0.278 Å	3.23
$^3J_{HH}$ indirect couplings	0.580 Hz	2.97
Total quality criterion n/χ^2		3.57
Total quality n/χ^2 - **2*p*** configuration		2.67
Total quality n/χ^2 - **2*r*** configuration		2.19
Total quality n/χ^2 - 11-epi-**2*r*** configuration		2.49

The assigned configuration is displayed in red.

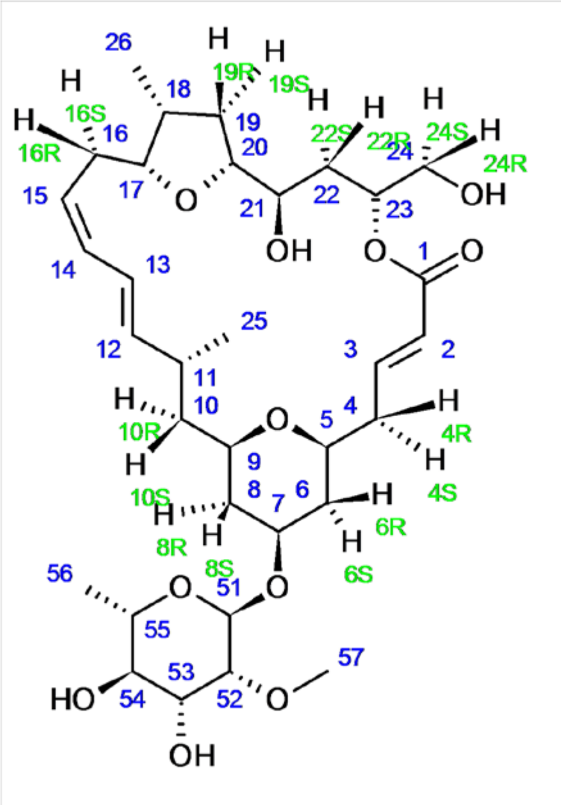

Figure S3: Nomenclature of Mandelalide A configuration **2*p*** (11-epi-**2*p*** differs only at position 11). The prochiral assignment of the protons is indicated with R and S resp. (in green).

Table S20: Experimental and calculated one bond CH residual dipolar splittings for Mandelalide A configuration **2p.** Values in red are outside the experimental estimated error margins ($\chi2>1$).

RDCs															
expDATA				MDOC: 2p			MDOC: 11-epi-2p			MDOC: 2r			MDOC: 11-epi-2r		
Atom A	Atom B	RDC value Exp. / Hz	Est. Err. / Hz	RDC value Calc. / Hz	Diff. Exp.-Calc. / Hz	χ2	RDC value Calc. / Hz	Diff. Exp.-Calc. / Hz	χ2	RDC value Calc. / Hz	Diff. Exp.-Calc. / Hz	χ2	RDC value Calc. / Hz	Diff. Exp.-Calc. / Hz	χ2
H17	C17	3.0	0.4	3.55	0.55	1.86	3.61	0.61	2.29	3.41	0.41	1.05	3.52	0.52	1.66
H16R	C16	10.0	1.9	8.94	1.07	0.31	9.00	1.00	0.28	8.38	1.62	0.72	8.26	1.74	0.84
H16S	C16	22.4	4.2	20.81	1.59	0.14	20.81	1.59	0.14	19.95	2.46	0.34	19.95	2.45	0.34
H14	C14	-3.4	0.4	-3.34	0.06	0.02	-3.33	0.07	0.04	-3.37	0.03	0.01	-3.24	0.16	0.17
H15	C15	17.8	1.4	17.06	0.74	0.28	17.13	0.67	0.23	16.81	1.00	0.51	16.54	1.26	0.81
H13	C13	-1.2	2.0	-1.40	0.20	0.01	-1.39	0.19	0.01	-1.49	0.29	0.02	-1.40	0.20	0.01
H12	C12	-3.8	0.7	-3.76	0.04	0.00	-3.74	0.06	0.01	-3.75	0.05	0.00	-3.67	0.13	0.03
H10S	C10	-11.7	4.2	-11.12	0.58	0.02	-10.93	0.77	0.03	-10.70	1.00	0.06	-10.37	1.33	0.10
H10R	C10	-3.5	2.9	-3.12	0.38	0.02	-2.87	0.63	0.05	-2.95	0.55	0.04	-2.66	0.84	0.08
H07	C07	-11.1	1.4	-10.65	0.45	0.10	-10.67	0.43	0.09	-10.34	0.76	0.29	-10.31	0.79	0.32
H08S	C08	-2.9	3.5	-3.63	0.73	0.04	-3.57	0.67	0.04	-3.88	0.98	0.08	-3.74	0.84	0.06
H08R	C08	1.5	2.7	0.14	1.36	0.25	0.63	0.87	0.10	-0.40	1.90	0.50	0.18	1.32	0.24
H09	C09	-5.3	5.2	-5.71	0.41	0.01	-5.68	0.38	0.01	-5.83	0.53	0.01	-5.74	0.44	0.01
H05	C05	-14.9	1.6	-13.86	1.04	0.42	-13.81	1.09	0.46	-13.24	1.66	1.08	-13.22	1.68	1.10
H06R	C06	-5.1	5.0	-5.46	0.36	0.01	-5.51	0.41	0.01	-5.55	0.45	0.01	-5.54	0.44	0.01
H04S	C04	23.3	5.8	21.98	1.32	0.05	22.02	1.28	0.05	21.04	2.26	0.15	20.96	2.34	0.16
H04R	C04	-13.4	2.6	-12.93	0.47	0.03	-12.98	0.43	0.03	-12.91	0.49	0.04	-12.91	0.49	0.04
H22R	C22	-10.5	1.4	-9.42	1.08	0.59	-9.44	1.06	0.57	-8.83	1.67	1.42	-8.75	1.75	1.57
H03	C03	10.1	1.4	9.96	0.15	0.01	9.94	0.16	0.01	9.79	0.31	0.05	9.81	0.29	0.04
H52	C52	6.3	0.2	5.99	0.31	2.39	6.03	0.27	1.85	5.82	0.48	5.81	5.97	0.33	2.74
H53	C53	4.5	1.3	4.52	0.02	0.00	4.47	0.03	0.00	4.53	0.03	0.00	4.56	0.06	0.00
H55	C55	8.8	0.7	8.52	0.28	0.16	8.48	0.32	0.21	8.39	0.41	0.34	8.36	0.45	0.40
H56a	C56	-2.3	2.0	-2.12	0.19	0.01	-2.10	0.20	0.01	-2.00	0.30	0.02	-2.09	0.21	0.01
H56c	C56	-2.3	2.0	-2.07	0.23	0.01	-2.14	0.17	0.01	-2.02	0.28	0.02	-2.06	0.24	0.01
H56b	C56	-2.3	2.0	-2.15	0.15	0.01	-2.14	0.16	0.01	-2.00	0.30	0.02	-2.06	0.24	0.01
H51	C51	-17.5	0.5	-16.74	0.76	2.29	-16.68	0.82	2.69	-16.25	1.25	6.22	-16.14	1.36	7.40
H57c	C57	-6.0	1.0	-5.58	0.42	0.17	-5.56	0.44	0.19	-5.13	0.87	0.76	-5.26	0.74	0.55
H57a	C57	-6.0	1.0	-5.54	0.46	0.21	-5.56	0.44	0.20	-5.14	0.86	0.73	-5.23	0.77	0.59
H57b	C57	-6.0	1.0	-5.59	0.41	0.16	-5.55	0.45	0.20	-5.10	0.90	0.81	-5.21	0.79	0.63
H54	C54	5.4	0.9	5.41	0.00	0.00	5.35	0.05	0.00	5.42	0.02	0.00	5.44	0.04	0.00
H02	C02	8.7	0.6	8.60	0.10	0.03	8.55	0.15	0.06	8.38	0.32	0.29	8.48	0.22	0.13
H20	C20	-15.2	0.5	-14.13	1.08	4.62	-14.21	0.99	3.90	-13.35	1.85	13.66	-13.31	1.89	14.35
H22S	C22	-5.9	1.4	-5.82	0.08	0.00	-5.76	0.14	0.01	-5.53	0.37	0.07	-5.46	0.44	0.10
H23	C23	-21.1	1.2	-19.90	1.20	1.00	-19.91	1.19	0.99	-18.98	2.12	3.11	-18.87	2.23	3.45
H24S	C24	13.9	1.5	13.41	0.49	0.11	13.45	0.45	0.09	13.27	0.64	0.18	13.24	0.66	0.20
H24R	C24	-1.6	0.9	-1.82	0.22	0.06	-1.78	0.18	0.04	-1.89	0.29	0.10	-1.94	0.34	0.14
H21	C21	7.7	3.3	6.44	1.26	0.15	6.59	1.11	0.11	6.06	1.65	0.25	6.03	1.67	0.26
H25c	C25	2.0	1.2	1.92	0.08	0.00	1.89	0.11	0.01	1.93	0.07	0.00	1.79	0.21	0.03
H25b	C25	2.0	1.2	1.91	0.09	0.01	1.88	0.12	0.01	1.99	0.01	0.00	1.77	0.23	0.04
H25a	C25	2.0	1.2	1.94	0.06	0.00	1.86	0.14	0.01	1.93	0.07	0.00	1.80	0.20	0.03
H11	C11	-7.9	0.7	-7.70	0.20	0.08	-7.85	0.05	0.00	-7.67	0.23	0.11	-7.61	0.30	0.18
H18	C18	1.5	0.4	1.22	0.28	0.50	1.22	0.28	0.50	0.87	0.63	2.50	0.78	0.72	3.22
H26a	C26	-2.7	1.1	-2.46	0.24	0.05	-2.44	0.26	0.06	-2.30	0.40	0.13	-2.37	0.33	0.09
H26b	C26	-2.7	1.1	-2.48	0.22	0.04	-2.46	0.25	0.05	-2.36	0.34	0.10	-2.36	0.34	0.10
H26c	C26	-2.7	1.1	-2.45	0.25	0.05	-2.44	0.26	0.05	-2.32	0.38	0.12	-2.31	0.39	0.13
				**n**		**45**	**n**		**45**	**n**		**45**	**n**		**45**
				**outliers**		**4**	**outliers**		**4**	**outliers**		**7**	**outliers**		**7**
				**F**		**41/45**	**F**		**41/45**	**F**		**38/45**	**F**		**38/45**
				**RMSD**		**0.64**	**RMSD**		**0.61**	**RMSD**		**0.99**	**RMSD**		**1.01**
				**min(1/X2)**		**0.22**	**min(1/X2)**		**0.26**	**min(1/X2)**		**0.07**	**min(1/X2)**		**0.07**
				**n/X2**		**2.76**	**n/X2**		**2.87**	**n/X2**		**1.08**	**n/X2**		**1.06**

Table S21: Experimental and calculated $^3J_{HH}$ couplings for Mandelalide A configuration **2p.** The error of the experiment and the calculation was estimated to 1 Hz. Values in red are outside the experimental estimated error margins ($\chi 2>1$).

RDCs															
expDATA				MDOC: 2p			MDOC: 11-epi-2p			MDOC: 2r			MDOC: 11-epi-2r		
Atom A	Atom B	RDC value Exp. / Hz	Err. / Hz	RDC value Calc. / Hz	Diff. Exp.-Calc. / Hz	χ2	RDC value Calc. / Hz	Diff. Exp.-Calc. / Hz	χ2	RDC value Calc. / Hz	Diff. Exp.-Calc. / Hz	χ2	RDC value Calc. / Hz	Diff. Exp.-Calc. / Hz	χ2
H17	H16S	8.6	1.0	8.00	0.60	0.36	7.93	0.67	0.44	7.64	0.96	0.92	7.66	0.94	0.89
H17	H16R	4.9	1.0	4.61	0.29	0.08	4.61	0.29	0.08	5.33	0.43	0.19	5.34	0.44	0.20
H17	H18	7.2	1.0	6.64	0.56	0.31	6.64	0.56	0.32	5.09	2.11	4.46	5.11	2.09	4.38
H19S	H20	8.8	1.0	8.66	0.14	0.02	8.64	0.16	0.03	7.87	0.93	0.86	7.86	0.94	0.89
H19R	H20	6.6	1.0	6.16	0.44	0.19	6.18	0.42	0.17	6.77	0.17	0.03	6.76	0.16	0.03
H19R	H18	6.8	1.0	6.30	0.50	0.25	6.29	0.51	0.26	7.99	1.19	1.42	8.13	1.33	1.76
H10S	H09	10.1	1.0	9.07	1.03	1.06	8.99	1.12	1.24	9.18	0.92	0.85	9.16	0.94	0.89
H10S	H11	5.1	1.0	5.20	0.10	0.01	5.45	0.35	0.12	4.98	0.13	0.02	5.46	0.36	0.13
H10R	H09	3.5	1.0	3.33	0.17	0.03	3.43	0.07	0.01	3.24	0.26	0.07	3.37	0.13	0.02
H07	H06S	4.7	1.0	3.17	1.54	2.36	3.17	1.54	2.36	3.18	1.52	2.31	3.17	1.54	2.36
H07	H06R	11.1	1.0	11.14	0.04	0.00	11.14	0.04	0.00	11.26	0.16	0.03	11.27	0.17	0.03
H07	H08R	4.7	1.0	4.26	0.44	0.19	4.25	0.45	0.20	4.28	0.42	0.18	4.27	0.43	0.19
H07	H08S	11.2	1.0	10.82	0.38	0.14	10.84	0.36	0.13	10.95	0.25	0.06	10.96	0.24	0.06
H08S	H09	11.2	1.0	10.94	0.26	0.07	10.96	0.24	0.06	11.05	0.15	0.02	11.05	0.15	0.02
H08R	H09	2.0	1.0	2.94	0.94	0.88	2.95	0.95	0.89	2.88	0.88	0.78	2.91	0.91	0.82
H05	H04R	3.8	1.0	4.36	0.56	0.32	4.34	0.54	0.29	4.32	0.52	0.27	4.31	0.51	0.26
H05	H04S	6.7	1.0	6.29	0.41	0.17	6.34	0.36	0.13	6.33	0.37	0.14	6.27	0.43	0.18
H05	H06S	2.0	1.0	2.39	0.39	0.15	2.40	0.40	0.16	2.37	0.37	0.13	2.37	0.37	0.13
H22R	H23	2.5	1.0	2.62	0.12	0.01	2.61	0.11	0.01	3.02	0.52	0.27	3.04	0.54	0.29
H22R	H21	10.5	1.0	10.20	0.30	0.09	10.23	0.27	0.07	9.02	1.49	2.21	9.02	1.48	2.19
H52	H51	1.5	1.0	2.61	1.11	1.24	2.76	1.26	1.58	2.47	0.97	0.94	2.33	0.83	0.68
H52	H53	3.4	1.0	3.17	0.24	0.06	3.19	0.21	0.04	3.13	0.27	0.07	3.15	0.25	0.06
H53	H54	9.4	1.0	9.07	0.33	0.11	8.83	0.58	0.33	9.31	0.09	0.01	9.53	0.13	0.02
H55	H56b	6.4	1.0	6.25	0.15	0.02	6.24	0.16	0.03	6.27	0.13	0.02	6.28	0.12	0.02
H55	H56c	6.4	1.0	6.24	0.16	0.03	6.24	0.16	0.03	6.27	0.13	0.02	6.26	0.14	0.02
H55	H56a	6.4	1.0	6.24	0.16	0.03	6.24	0.16	0.02	6.27	0.13	0.02	6.27	0.13	0.02
H55	H54	9.5	1.0	8.76	0.74	0.54	8.57	0.93	0.87	8.98	0.52	0.27	9.15	0.35	0.12
H20	H21	8.1	1.0	7.78	0.32	0.10	7.77	0.34	0.11	7.66	0.44	0.19	7.74	0.36	0.13
H22S	H23	10.9	1.0	10.17	0.73	0.54	10.16	0.74	0.55	9.42	1.48	2.20	9.41	1.49	2.21
H22S	H21	2.1	1.0	2.35	0.25	0.06	2.35	0.25	0.06	3.13	1.03	1.07	3.20	1.10	1.20
H23	H24R	5.6	1.0	5.75	0.15	0.02	5.75	0.15	0.02	5.72	0.12	0.01	5.71	0.11	0.01
H23	H24S	3.4	1.0	4.11	0.71	0.50	4.11	0.71	0.51	3.87	0.47	0.22	3.87	0.47	0.22
H25c	H11	6.8	1.0	6.57	0.23	0.05	6.56	0.24	0.06	6.57	0.23	0.05	6.55	0.25	0.06
H25b	H11	6.8	1.0	6.57	0.23	0.05	6.56	0.24	0.06	6.56	0.24	0.06	6.54	0.26	0.07
H25a	H11	6.8	1.0	6.57	0.23	0.05	6.56	0.24	0.06	6.56	0.24	0.06	6.55	0.25	0.06
H18	H26c	7.0	1.0	6.63	0.37	0.14	6.62	0.38	0.14	6.61	0.40	0.16	6.60	0.40	0.16
H18	H26b	7.0	1.0	6.62	0.38	0.15	6.63	0.37	0.14	6.60	0.40	0.16	6.61	0.39	0.15
H18	H26a	7.0	1.0	6.63	0.37	0.14	6.63	0.37	0.14	6.61	0.39	0.15	6.61	0.40	0.16

	MDOC: 2p	MDOC: 11-epi-2p	MDOC: 2r	MDOC: 11-epi-2r
**n**	**38**	**38**	**38**	**38**
**outliers**	**3**	**3**	**6**	**6**
**F**	**35/38**	**35/38**	**32/38**	**32/38**
**RMSD**	**0.53**	**0.56**	**0.74**	**0.74**
**min(1/X2)**	**0.42**	**0.42**	**0.22**	**0.23**
**n/X2**	**3.61**	**3.24**	**1.82**	**1.80**

Table S22: Experimental and calculated NOE distances for Mandelalide A configuration **2*p*.** The error of the experiment estimated to 0.5 Å. NOE distances to OH-groups are not taken as constraints. Values in red are outside the experimental estimated error margins ($\chi2>1$).

NOEs														
expDATA				MDOC: 2p			MDOC: 11-epi-2p			MDOC: 2r			MDOC: 11-epi-2r	
Atom A	Atom B	NOE Dist. Exp. / Å	Err. / Å	NOE Dist. Calc. / Å	Diff. Exp.-Calc. / Å	χ2	NOE Dist. Calc. / Å	Diff. Exp.-Calc. / Å	χ2	NOE Dist. Calc. / Å	Diff. Exp.-Calc. / Å	χ2	NOE Dist. Calc. / Å	Diff. Exp.-Calc. / Å	χ2
H17	H16R	2.4	0.5	2.51	0.15	0.09	2.50	0.14	0.08	2.59	0.23	0.21	2.59	0.23	0.21
H17	H16S	2.7	0.5	2.69	0.04	0.01	2.69	0.04	0.01	2.50	0.15	0.09	2.50	0.15	0.09
H17	H15	2.3	0.5	2.76	0.50	1.01	2.76	0.50	1.02	2.87	0.61	1.47	2.85	0.59	1.37
H17	H20	2.9	0.5	2.85	0.05	0.01	2.87	0.03	0.00	2.80	0.10	0.04	2.85	0.05	0.01
H17	H18	2.0	0.5	2.31	0.30	0.35	2.31	0.30	0.35	2.34	0.33	0.43	2.34	0.33	0.42
H17	H26a	3.6	0.5	3.38	0.19	0.15	3.37	0.20	0.17	3.46	0.11	0.05	3.44	0.13	0.07
H17	H26b	3.6	0.5	3.37	0.20	0.16	3.36	0.21	0.17	3.46	0.11	0.04	3.45	0.12	0.06
H17	H26c	3.6	0.5	3.38	0.19	0.15	3.37	0.20	0.16	3.46	0.11	0.05	3.45	0.12	0.06
H19S	H19R	1.8	0.5	1.80	0.02	0.00	1.80	0.02	0.00	1.76	0.02	0.00	1.76	0.02	0.00
H19S	H16R	3.1	0.5	2.95	0.19	0.14	2.96	0.18	0.12	4.23	1.09	4.73	4.21	1.07	4.61
H19S	H20	3.2	0.5	2.96	0.20	0.16	2.96	0.20	0.16	2.29	0.88	3.06	2.29	0.87	3.05
H19S	H22S	2.6	0.5	2.64	0.08	0.03	2.63	0.07	0.02	3.08	0.52	1.06	3.07	0.51	1.04
H19S	H21	2.4	0.5	2.65	0.29	0.33	2.64	0.28	0.32	3.28	0.92	3.39	3.31	0.95	3.61
H19S	H26a	2.8	0.5	2.86	0.02	0.00	2.86	0.02	0.00	3.19	0.35	0.48	3.19	0.35	0.49
H19S	H26b	2.8	0.5	2.87	0.03	0.00	2.87	0.03	0.00	3.18	0.34	0.47	3.18	0.34	0.46
H19S	H26c	2.8	0.5	2.87	0.03	0.00	2.87	0.03	0.00	3.19	0.35	0.48	3.18	0.34	0.46
H19R	H20	2.2	0.5	2.36	0.17	0.11	2.36	0.17	0.11	2.93	0.74	2.20	2.93	0.74	2.18
H19R	H22S	2.9	0.5	2.84	0.02	0.00	2.84	0.02	0.00	2.96	0.10	0.04	2.96	0.10	0.04
H19R	H18	2.3	0.5	2.36	0.09	0.03	2.36	0.09	0.03	2.86	0.59	1.40	2.87	0.60	1.44
H19R	H26a	3.6	0.5	3.27	0.35	0.48	3.27	0.35	0.49	2.81	0.81	2.64	2.82	0.80	2.56
H19R	H26b	3.6	0.5	3.28	0.34	0.46	3.28	0.35	0.48	2.82	0.81	2.59	2.81	0.81	2.63
H19R	H26c	3.6	0.5	3.27	0.35	0.49	3.27	0.35	0.49	2.82	0.80	2.59	2.83	0.79	2.50
H16R	H16S	2.0	0.5	1.77	0.19	0.14	1.77	0.19	0.14	1.76	0.20	0.16	1.76	0.20	0.15
H16R	H15	2.5	0.5	2.54	0.09	0.03	2.55	0.09	0.04	2.63	0.18	0.12	2.64	0.19	0.14
H16R	H13	2.5	0.5	2.62	0.16	0.10	2.62	0.16	0.11	2.50	0.04	0.01	2.52	0.06	0.01
H16R	H26a	2.9	0.5	2.91	0.06	0.01	2.91	0.06	0.02	2.98	0.13	0.06	2.97	0.12	0.05
H16R	H26b	2.9	0.5	2.91	0.06	0.02	2.91	0.06	0.02	2.98	0.13	0.06	2.96	0.11	0.05
H16R	H26c	2.9	0.5	2.92	0.07	0.02	2.92	0.07	0.02	2.98	0.13	0.07	2.98	0.13	0.07
H16S	H15	2.6	0.5	2.69	0.12	0.06	2.68	0.11	0.05	2.58	0.01	0.00	2.61	0.04	0.00
H16S	H13	2.3	0.5	2.44	0.15	0.09	2.46	0.17	0.12	2.54	0.25	0.26	2.53	0.24	0.24
H16S	H26a	3.4	0.5	3.10	0.33	0.44	3.10	0.33	0.43	2.99	0.44	0.78	3.01	0.42	0.71
H16S	H26b	3.4	0.5	3.10	0.33	0.43	3.10	0.33	0.44	2.99	0.44	0.77	3.02	0.42	0.69
H16S	H26c	3.4	0.5	3.11	0.32	0.42	3.11	0.32	0.42	2.99	0.44	0.78	3.01	0.42	0.71
H14	H15	2.1	0.5	2.32	0.25	0.25	2.32	0.25	0.24	2.33	0.26	0.26	2.32	0.25	0.26
H14	H12	2.1	0.5	2.41	0.30	0.36	2.40	0.29	0.33	2.43	0.32	0.41	2.42	0.31	0.38
H15	H13	3.7	0.5	3.77	0.05	0.01	3.77	0.05	0.01	3.77	0.04	0.01	3.77	0.05	0.01
H15	H26a	4.0	0.5	3.53	0.48	0.90	3.49	0.51	1.03	3.55	0.45	0.82	3.52	0.48	0.92
H15	H26b	4.0	0.5	3.52	0.48	0.91	3.51	0.49	0.97	3.54	0.46	0.85	3.50	0.50	0.99
H15	H26c	4.0	0.5	3.54	0.46	0.85	3.50	0.50	1.00	3.53	0.47	0.89	3.51	0.49	0.96
H13	H12	2.9	0.5	3.02	0.15	0.09	3.02	0.15	0.09	3.02	0.15	0.09	3.02	0.15	0.09
H13	H10S	2.8	0.5	2.96	0.12	0.05	2.94	0.10	0.04	2.88	0.04	0.01	2.86	0.02	0.00
H13	H09	3.2	0.5	3.07	0.15	0.09	3.09	0.13	0.07	3.12	0.10	0.04	3.17	0.05	0.01
H13	H21	3.1	0.5	3.37	0.31	0.37	3.41	0.35	0.48	3.19	0.13	0.07	3.28	0.22	0.18
H13	H25c	3.0	0.5	3.04	0.02	0.00	3.02	0.00	0.00	3.02	0.00	0.00	3.02	0.00	0.00
H13	H25b	3.0	0.5	3.04	0.02	0.00	3.02	0.00	0.00	3.03	0.01	0.00	3.02	0.00	0.00
H13	H25a	3.0	0.5	3.04	0.02	0.00	3.02	0.00	0.00	3.03	0.00	0.00	3.03	0.01	0.00
H13	H11	2.4	0.5	2.66	0.25	0.25	2.76	0.35	0.48	2.66	0.25	0.25	2.75	0.34	0.45
H12	H10S	2.7	0.5	2.92	0.22	0.20	2.95	0.25	0.25	2.98	0.28	0.32	3.00	0.30	0.36
H12	H10R	2.9	0.5	2.95	0.06	0.01	3.02	0.13	0.07	2.91	0.02	0.00	3.00	0.11	0.05
H12	H09	3.5	0.5	3.28	0.17	0.11	3.25	0.20	0.15	3.19	0.26	0.27	3.19	0.26	0.27
H12	H25c	3.1	0.5	3.11	0.05	0.01	3.11	0.04	0.01	3.13	0.07	0.02	3.11	0.05	0.01
H12	H25b	3.1	0.5	3.11	0.05	0.01	3.11	0.05	0.01	3.12	0.06	0.01	3.11	0.05	0.01
H12	H25a	3.1	0.5	3.11	0.05	0.01	3.11	0.05	0.01	3.13	0.07	0.02	3.11	0.05	0.01
H12	H11	2.4	0.5	2.61	0.21	0.17	2.57	0.17	0.11	2.62	0.22	0.19	2.59	0.19	0.15
H10S	H10R	1.8	0.5	1.77	0.01	0.00	1.77	0.01	0.00	1.78	0.01	0.00	1.77	0.01	0.00
H10S	H08S	2.7	0.5	2.67	0.01	0.00	2.70	0.03	0.00	2.66	0.00	0.00	2.68	0.02	0.00
H10S	H09	3.1	0.5	2.73	0.33	0.43	2.71	0.35	0.49	2.77	0.29	0.34	2.75	0.31	0.39
H10S	H25c	3.7	0.5	3.17	0.56	1.23	3.07	0.65	1.69	3.22	0.50	1.00	3.07	0.65	1.68
H10S	H25b	3.7	0.5	3.18	0.55	1.19	3.07	0.65	1.67	3.23	0.49	0.96	3.05	0.67	1.78
H10S	H25a	3.7	0.5	3.17	0.55	1.19	3.07	0.65	1.67	3.22	0.50	1.00	3.06	0.66	1.75
H10S	H11	2.6	0.5	2.49	0.08	0.02	2.53	0.04	0.01	2.47	0.11	0.04	2.56	0.01	0.00
H10R	H09	2.2	0.5	2.48	0.24	0.22	2.49	0.25	0.25	2.47	0.23	0.22	2.49	0.25	0.25
H10R	H25c	2.9	0.5	2.99	0.07	0.02	3.05	0.13	0.07	3.01	0.09	0.04	3.07	0.15	0.09
H10R	H25b	2.9	0.5	2.99	0.07	0.02	3.05	0.13	0.06	3.00	0.08	0.03	3.07	0.15	0.09

H10R	H25a	2.9	0.5	2.99	0.07	0.02	3.04	0.12	0.06	3.01	0.09	0.03	3.07	0.15	0.09
H07	H08R	2.4	0.5	2.49	0.14	0.08	2.49	0.14	0.08	2.50	0.15	0.09	2.50	0.15	0.09
H07	H09	2.4	0.5	2.55	0.16	0.10	2.55	0.16	0.10	2.56	0.17	0.11	2.56	0.17	0.12
H07	H05	2.4	0.5	2.60	0.20	0.15	2.60	0.20	0.15	2.61	0.21	0.18	2.61	0.21	0.18
H07	H06R	3.1	0.5	3.04	0.10	0.04	3.04	0.10	0.04	3.05	0.09	0.03	3.05	0.09	0.03
H07	H06S	2.3	0.5	2.44	0.12	0.05	2.43	0.11	0.05	2.44	0.12	0.05	2.44	0.12	0.06
H08S	H08R	1.8	0.5	1.77	0.01	0.00	1.77	0.01	0.00	1.77	0.01	0.00	1.77	0.01	0.00
H08R	H09	2.5	0.5	2.49	0.01	0.00	2.49	0.02	0.00	2.49	0.02	0.00	2.49	0.02	0.00
H08R	H55	2.9	0.5	3.12	0.23	0.20	3.13	0.24	0.22	3.08	0.19	0.14	3.09	0.20	0.15
H08R	H11	3.0	0.5	3.44	0.41	0.67	3.40	0.37	0.55	3.44	0.41	0.67	3.42	0.39	0.60
H09	H05	2.1	0.5	2.32	0.22	0.19	2.32	0.22	0.20	2.32	0.22	0.18	2.32	0.22	0.19
H09	H25c	2.9	0.5	3.01	0.09	0.03	3.03	0.11	0.05	2.97	0.05	0.01	3.03	0.11	0.04
H09	H25b	2.9	0.5	3.02	0.10	0.04	3.01	0.09	0.03	2.98	0.06	0.01	3.03	0.11	0.05
H09	H25a	2.9	0.5	3.02	0.10	0.04	3.03	0.11	0.05	2.99	0.07	0.02	3.02	0.10	0.04
H09	H11	2.8	0.5	2.94	0.13	0.07	2.96	0.15	0.09	3.02	0.21	0.18	2.93	0.12	0.05
H05	H06S	2.4	0.5	2.45	0.06	0.01	2.45	0.06	0.01	2.45	0.06	0.01	2.45	0.06	0.02
H05	H04S	2.4	0.5	2.48	0.13	0.07	2.49	0.14	0.08	2.48	0.13	0.07	2.47	0.12	0.06
H05	H04R	2.4	0.5	2.59	0.22	0.20	2.58	0.21	0.17	2.60	0.23	0.21	2.60	0.23	0.21
H05	H03	2.7	0.5	3.13	0.43	0.74	3.13	0.43	0.75	3.14	0.44	0.76	3.07	0.37	0.54
H05	H02	2.8	0.5	2.88	0.07	0.02	2.88	0.07	0.02	2.92	0.11	0.05	3.04	0.23	0.21
H06R	H06S	1.8	0.5	1.79	0.04	0.01	1.79	0.04	0.01	1.79	0.04	0.01	1.79	0.04	0.01
H06R	H03	2.7	0.5	2.99	0.33	0.44	2.99	0.33	0.44	2.98	0.32	0.40	2.96	0.30	0.35
H06S	H04R	3.2	0.5	2.84	0.38	0.59	2.84	0.38	0.58	2.84	0.38	0.57	2.82	0.40	0.65
H04S	H04R	1.8	0.5	1.78	0.03	0.00	1.78	0.03	0.00	1.78	0.03	0.00	1.78	0.03	0.00
H04S	H03	2.4	0.5	2.57	0.15	0.09	2.57	0.15	0.09	2.58	0.16	0.10	2.59	0.17	0.11
H04S	H02	2.9	0.5	2.84	0.10	0.04	2.82	0.13	0.06	2.80	0.14	0.08	2.76	0.18	0.13
H04R	H03	2.6	0.5	2.60	0.00	0.00	2.61	0.01	0.00	2.61	0.01	0.00	2.65	0.05	0.01
H04R	H02	2.3	0.5	2.67	0.37	0.56	2.66	0.36	0.52	2.65	0.35	0.50	2.62	0.32	0.42
H22R	H20	2.4	0.5	2.69	0.29	0.35	2.70	0.30	0.35	2.87	0.47	0.88	2.87	0.47	0.89
H22R	H22S	1.8	0.5	1.77	0.04	0.01	1.77	0.04	0.01	1.73	0.08	0.03	1.73	0.08	0.03
H22R	H23	2.3	0.5	2.47	0.15	0.09	2.47	0.15	0.09	2.54	0.22	0.19	2.54	0.22	0.20
H22R	H24S	2.7	0.5	2.76	0.09	0.03	2.77	0.10	0.04	3.01	0.34	0.46	3.01	0.34	0.45
H22R	H24R	3.0	0.5	2.97	0.01	0.00	2.97	0.02	0.00	3.26	0.31	0.39	3.25	0.30	0.35
H22R	H21	3.3	0.5	2.87	0.46	0.84	2.87	0.46	0.84	2.46	0.87	3.06	2.44	0.90	3.20
H03	H02	2.8	0.5	3.02	0.27	0.29	3.02	0.27	0.29	3.02	0.27	0.29	3.02	0.27	0.29
H03	H25c	4.3	0.5	4.33	0.05	0.01	4.25	0.03	0.00	4.35	0.06	0.02	4.18	0.10	0.04
H03	H25b	4.3	0.5	4.30	0.02	0.00	4.23	0.05	0.01	4.30	0.02	0.00	4.15	0.13	0.07
H03	H25a	4.3	0.5	4.32	0.04	0.01	4.23	0.05	0.01	4.29	0.00	0.00	4.14	0.14	0.08
H52	H53	2.1	0.5	2.41	0.31	0.39	2.41	0.31	0.38	2.42	0.32	0.40	2.42	0.32	0.40
H53	H54	3.0	0.5	2.97	0.06	0.02	2.95	0.09	0.03	2.98	0.05	0.01	2.99	0.04	0.01
H55	H54	3.6	0.5	2.97	0.58	1.36	2.95	0.60	1.44	2.98	0.57	1.30	2.99	0.56	1.24
H02	H23	3.8	0.5	3.00	0.83	2.72	3.06	0.76	2.29	3.08	0.74	2.18	3.85	0.03	0.00
H02	H25c	3.8	0.5	3.86	0.08	0.03	3.84	0.06	0.02	3.88	0.10	0.04	3.89	0.11	0.04
H02	H25b	3.8	0.5	3.88	0.10	0.04	3.87	0.09	0.03	3.89	0.11	0.05	3.88	0.10	0.04
H02	H25a	3.8	0.5	3.85	0.07	0.02	3.84	0.06	0.02	3.91	0.13	0.06	3.89	0.11	0.05
H20	H22S	3.1	0.5	3.05	0.09	0.03	3.05	0.09	0.04	2.90	0.25	0.24	2.87	0.27	0.29
H20	H21	2.9	0.5	2.80	0.11	0.05	2.80	0.11	0.05	2.68	0.23	0.21	2.69	0.22	0.20
H20	H18	2.6	0.5	2.66	0.11	0.04	2.66	0.11	0.05	2.69	0.14	0.08	2.68	0.13	0.07
H22S	H23	2.8	0.5	2.85	0.01	0.00	2.84	0.00	0.00	2.59	0.25	0.25	2.58	0.26	0.27
H22S	H24S	3.2	0.5	3.19	0.06	0.01	3.17	0.07	0.02	2.97	0.27	0.29	2.97	0.27	0.29
H22S	H24R	2.9	0.5	2.95	0.06	0.01	2.94	0.05	0.01	2.96	0.07	0.02	2.96	0.07	0.02
H22S	H21	2.5	0.5	2.51	0.01	0.00	2.51	0.01	0.00	2.62	0.12	0.05	2.63	0.13	0.07
H23	H24S	2.2	0.5	2.52	0.30	0.36	2.52	0.30	0.36	2.54	0.32	0.41	2.54	0.32	0.41
H23	H24R	2.3	0.5	2.59	0.25	0.25	2.59	0.25	0.24	2.60	0.26	0.26	2.60	0.26	0.26
H23	H21	2.6	0.5	2.91	0.28	0.31	2.91	0.28	0.32	3.00	0.37	0.54	3.00	0.37	0.53
H24S	H24R	1.7	0.5	1.76	0.09	0.03	1.76	0.09	0.03	1.77	0.10	0.04	1.76	0.09	0.04
H21	H26a	3.4	0.5	3.51	0.07	0.02	3.51	0.07	0.02	3.50	0.06	0.01	3.47	0.03	0.00
H21	H26b	3.4	0.5	3.49	0.05	0.01	3.50	0.06	0.02	3.50	0.06	0.01	3.50	0.06	0.02
H21	H26c	3.4	0.5	3.49	0.05	0.01	3.50	0.06	0.01	3.49	0.05	0.01	3.46	0.02	0.00
H25c	H11	2.6	0.5	2.55	0.01	0.00	2.55	0.01	0.00	2.55	0.01	0.00	2.55	0.01	0.00
H25b	H11	2.6	0.5	2.55	0.01	0.00	2.55	0.01	0.00	2.56	0.00	0.00	2.55	0.01	0.00
H25a	H11	2.6	0.5	2.55	0.01	0.00	2.55	0.01	0.00	2.55	0.01	0.00	2.55	0.01	0.00
H18	H26a	2.6	0.5	2.54	0.06	0.01	2.54	0.06	0.01	2.53	0.07	0.02	2.53	0.07	0.02
H18	H26b	2.6	0.5	2.54	0.06	0.02	2.54	0.06	0.02	2.53	0.07	0.02	2.53	0.07	0.02
H18	H26c	2.6	0.5	2.54	0.06	0.02	2.54	0.06	0.02	2.53	0.07	0.02	2.52	0.08	0.02

**n**	**129**	**n**	**129**	**n**	**129**	**n**	**129**
**outliers**	**6**	**outliers**	**7**	**outliers**	**14**	**outliers**	**15**
**F**	**123/129**	**F**	**122/129**	**F**	**115/129**	**F**	**114/129**
**RMSD**	**0.23**	**RMSD**	**0.24**	**RMSD**	**0.33**	**RMSD**	**0.33**
**min(1/X2)**	**0.37**	**min(1/X2)**	**0.44**	**min(1/X2)**	**0.21**	**min(1/X2)**	**0.22**
**n/X2**	**4.82**	**n/X2**	**4.49**	**n/X2**	**2.34**	**n/X2**	**2.32**

Table S23: Summary of fitting scores ((#outliers, Fidelity, min(1/χ^2), n/χ^2) for the ensemble of data(Table Table S20, Table S21, and Table S22) used in the MDOC simulation of Mandelalide A configuration **2*p*.**

Mandelalide A **2*p***	MDOC: 2p		MDOC: 11-epi-2p		MDOC: 2r		MDOC: 11-epi-2r	
	**outliers**	**13**	**outliers**	**14**	**outliers**	**27**	**outliers**	**28**
	**F**	**199/212**	**F**	**198/212**	**F**	**185/212**	**F**	**184/212**
	**n**	**212**	**n**	**212**	**n**	**212**	**n**	**212**
	**min(1/X2)**	**0.22**	**min(1/X2)**	**0.26**	**min(1/X2)**	**0.07**	**min(1/X2)**	**0.07**
	**n/X2**	**3.95**	**n/X2**	**3.78**	**n/X2**	**1.80**	**n/X2**	**1.78**

Table S24: Experimental and calculated one bond CH residual dipolar splittings for Mandelalide A configuration 11-epi-**2*p*.** Values in red are outside the experimental estimated error margins ($\chi2>1$).

RDCs														
expDATA				MDOC: 2p			MDOC: 11-epi-2p			MDOC: 2r			MDOC: 11-epi-2r	
Atom A	Atom B	RDC value Exp. / Hz	Err. / Hz	RDC value Calc. / Hz	Diff. Exp.-Calc. / Hz	χ2	RDC value Calc. / Hz	Diff. Exp.-Calc. / Hz	χ2	RDC value Calc. / Hz	Diff. Exp.-Calc. / Hz	χ2	RDC value Calc. / Hz	Diff. Exp.-Calc. / Hz	χ2
H17	C17	11.7	1.7	10.71	0.99	0.34	10.97	0.73	0.19	10.93	0.77	0.21	10.95	0.75	0.19
H19S	C19	7.9	1.7	7.50	0.40	0.06	7.77	0.13	0.01	7.78	0.12	0.00	7.77	0.13	0.01
H19R	C19	-5.6	1.5	-5.45	0.15	0.01	-5.37	0.23	0.02	-5.08	0.52	0.12	-5.21	0.39	0.07
H16R	C16	-0.7	4.6	-0.48	0.22	0.00	-0.41	0.29	0.00	-0.50	0.20	0.00	-0.30	0.40	0.01
H16S	C16	4	3.8	4.01	0.01	0.00	4.13	0.13	0.00	4.04	0.04	0.00	4.07	0.07	0.00
H14	C14	-7.3	3.5	-7.10	0.20	0.00	-7.19	0.11	0.00	-7.09	0.21	0.00	-7.02	0.28	0.01
H15	C15	11	1.1	10.37	0.63	0.33	10.36	0.64	0.34	10.14	0.86	0.61	10.26	0.74	0.46
H13	C13	-12.4	3.1	-11.63	0.77	0.06	-11.49	0.91	0.09	-11.58	0.82	0.07	-11.57	0.83	0.07
H12	C12	-3.9	4.7	-4.42	0.52	0.01	-4.46	0.56	0.01	-4.41	0.51	0.01	-4.47	0.57	0.01
H10S	C10	10.8	3.9	8.07	2.73	0.49	10.51	0.29	0.01	10.55	0.25	0.00	10.47	0.33	0.01
H10R	C10	-15.6	3.8	-11.47	4.13	1.18	-14.95	0.65	0.03	-14.89	0.71	0.03	-15.00	0.60	0.02
H07	C07	6.4	3.4	6.18	0.22	0.00	6.81	0.41	0.01	6.70	0.30	0.01	6.79	0.39	0.01
H08S	C08	8.6	3.1	8.40	0.20	0.00	8.85	0.25	0.01	8.73	0.13	0.00	8.85	0.25	0.01
H08R	C08	-13.5	1.1	-12.44	1.06	0.94	-12.49	1.01	0.85	-12.35	1.15	1.10	-12.40	1.10	0.99
H09	C09	11.3	3.7	11.03	0.27	0.01	11.10	0.20	0.00	10.98	0.32	0.01	11.00	0.30	0.01
H05	C05	8.6	2.3	8.31	0.29	0.02	8.70	0.10	0.00	8.53	0.07	0.00	8.70	0.10	0.00
H06R	C06	13.4	3.7	11.90	1.50	0.16	12.53	0.88	0.06	12.41	0.99	0.07	12.59	0.81	0.05
H06S	C06	7.2	1	6.23	0.97	0.94	5.84	1.36	1.85	5.55	1.66	2.74	6.06	1.14	1.30
H04S	C04	7.9	1.8	6.38	1.53	0.72	6.53	1.37	0.58	6.00	1.90	1.11	6.49	1.41	0.61
H04R	C04	12.3	1.6	10.99	1.31	0.67	11.27	1.03	0.42	11.12	1.18	0.54	11.45	0.85	0.28
H22R	C22	2.7	1.1	2.60	0.10	0.01	2.54	0.16	0.02	2.58	0.12	0.01	2.53	0.17	0.03
H03	C03	4.7	1.6	4.81	0.11	0.01	4.90	0.20	0.02	4.84	0.14	0.01	4.94	0.24	0.02
H52	C52	6.7	2	5.99	0.72	0.13	6.36	0.34	0.03	6.24	0.46	0.05	6.24	0.46	0.05
H53	C53	-5.5	0.6	-5.33	0.17	0.08	-5.48	0.02	0.00	-5.54	0.04	0.00	-5.53	0.03	0.00
H55	C55	-4.6	0.6	-4.36	0.24	0.16	-4.62	0.02	0.00	-4.68	0.08	0.02	-4.67	0.07	0.01
H56a	C56	-1.3	0.4	-1.37	0.07	0.03	-1.34	0.04	0.01	-1.33	0.03	0.01	-1.38	0.08	0.04
H56c	C56	-1.3	0.4	-1.34	0.03	0.01	-1.41	0.11	0.08	-1.33	0.03	0.01	-1.33	0.03	0.00
H56b	C56	-1.3	0.4	-1.32	0.02	0.00	-1.38	0.08	0.04	-1.37	0.07	0.03	-1.38	0.08	0.04

H51	C51	-4.8	1.4	-4.36	0.44	0.10	-4.15	0.65	0.22	-4.25	0.55	0.15	-4.25	0.55	0.16
H57c	C57	-6.7	1.6	-6.21	0.49	0.09	-5.88	0.82	0.26	-5.85	0.85	0.28	-5.84	0.86	0.29
H57a	C57	-6.7	1.6	-6.31	0.39	0.06	-5.86	0.84	0.27	-5.87	0.84	0.27	-5.89	0.81	0.26
H57b	C57	-6.7	1.6	-6.52	0.18	0.01	-5.90	0.80	0.25	-5.87	0.83	0.27	-5.88	0.82	0.26
H54	C54	-9	0.8	-8.43	0.57	0.50	-8.58	0.42	0.28	-8.64	0.36	0.20	-8.65	0.35	0.19
H02	C02	7.5	0.5	7.18	0.32	0.42	7.21	0.29	0.33	7.16	0.34	0.46	7.19	0.31	0.37
H20	C20	-5.2	1.6	-4.60	0.60	0.14	-4.57	0.63	0.15	-4.70	0.50	0.10	-4.69	0.51	0.10
H22S	C22	-6	1.6	-5.35	0.65	0.16	-5.43	0.58	0.13	-5.51	0.49	0.09	-5.51	0.49	0.09
H23	C23	4.6	0.9	3.96	0.64	0.51	3.85	0.75	0.70	4.05	0.55	0.37	4.06	0.54	0.36
H24S	C24	8.5	1.9	7.85	0.65	0.12	7.94	0.56	0.09	7.93	0.57	0.09	7.98	0.52	0.07
H24R	C24	3.8	0.4	3.38	0.42	1.09	3.42	0.38	0.91	3.36	0.45	1.24	3.43	0.37	0.87
H21	C21	0.8	2.6	0.57	0.23	0.01	0.66	0.14	0.00	0.66	0.15	0.00	0.58	0.23	0.01
H25c	C25	-1	1	-1.01	0.01	0.00	-1.10	0.10	0.01	-1.15	0.15	0.02	-1.04	0.04	0.00
H25b	C25	-1	1	-0.96	0.04	0.00	-1.09	0.09	0.01	-1.21	0.21	0.04	-1.08	0.08	0.01
H25a	C25	-1	1	-0.96	0.04	0.00	-1.03	0.03	0.00	-1.19	0.19	0.03	-1.06	0.06	0.00
H11	C11	-15	2.5	-13.51	1.49	0.35	-13.72	1.28	0.26	-13.56	1.45	0.33	-13.75	1.25	0.25
H18	C18	7.9	2.3	7.42	0.49	0.04	7.50	0.40	0.03	7.08	0.82	0.13	7.11	0.79	0.12
H26a	C26	0.7	0.6	0.76	0.06	0.01	0.86	0.16	0.07	0.83	0.13	0.05	0.72	0.02	0.00
H26b	C26	0.7	0.6	0.70	0.00	0.00	0.91	0.21	0.13	0.84	0.14	0.06	0.77	0.07	0.01
H26c	C26	0.7	0.6	0.75	0.05	0.01	0.86	0.16	0.07	0.92	0.22	0.13	0.76	0.06	0.01
				**n**		**48**	**n**		**48**	**n**		**48**	**n**		**48**
				**outliers**		**2**	**outliers**		**1**	**outliers**		**4**	**outliers**		**1**
				**F**		**46/48**	**F**		**47/48**	**F**		**44/48**	**F**		**47/48**
				**RMSD**		**0.93**	**RMSD**		**0.58**	**RMSD**		**0.66**	**RMSD**		**0.57**
				**min(1/X2)**		**0.85**	**min(1/X2)**		**0.54**	**min(1/X2)**		**0.37**	**min(1/X2)**		**0.77**
				**n/X2**		**4.81**	**n/X2**		**5.43**	**n/X2**		**4.32**	**n/X2**		**6.19**

Table S25: Experimental and calculated $^3J_{HH}$ couplings for Mandelalide A configuration 11-epi-**2.** The error of the experiment and the calculation was estimated to 1 Hz. Values in red are outside the experimental estimated error margins ($\chi 2>1$).

J-couplings															
expDATA				MDOC: 2p			MDOC: 11-epi-2p			MDOC: 2r			MDOC: 11-epi-2r		
Atom A	Atom B	$^3J_{HH}$ coupl. Exp./ Hz	Err. / Hz	$^3J_{HH}$ coupl. Calc. / Hz	Diff. Exp.- Calc. / Hz	χ2	$^3J_{HH}$ coupl. Calc. / Hz	Diff. Exp.- Calc. / Hz	χ2	$^3J_{HH}$ coupl. Calc. / Hz	Diff. Exp.- Calc. / Hz	χ2	$^3J_{HH}$ coupl. Calc. / Hz	Diff. Exp.- Calc. / Hz	χ2
H17	H16S	7.6	1	6.82	0.78	0.61	6.74	0.86	0.75	7.17	0.43	0.18	7.06	0.54	0.30
H17	H16R	6	1	5.81	0.19	0.03	5.80	0.21	0.04	5.89	0.11	0.01	5.87	0.13	0.02
H17	H18	7.3	1	6.63	0.67	0.45	6.68	0.62	0.38	5.70	1.60	2.57	5.62	1.68	2.81
H19R	H20	7	1	6.74	0.27	0.07	6.62	0.38	0.14	6.90	0.10	0.01	6.88	0.12	0.01
H19R	H18	7	1	6.50	0.50	0.25	6.43	0.58	0.33	7.74	0.74	0.55	7.56	0.56	0.31
H10S	H09	11	1	10.44	0.56	0.31	10.10	0.90	0.82	10.26	0.74	0.55	10.30	0.70	0.49
H10S	H11	2.7	1	3.32	0.62	0.38	3.27	0.57	0.32	3.30	0.60	0.36	3.39	0.69	0.48
H10R	H09	1.4	1	1.83	0.43	0.18	1.91	0.51	0.26	1.94	0.54	0.29	1.86	0.46	0.21
H10R	H11	11.2	1	9.07	2.13	4.53	10.11	1.09	1.19	9.31	1.89	3.58	10.53	0.67	0.45
H07	H06S	4.6	1	3.17	1.43	2.04	3.17	1.43	2.04	3.17	1.43	2.05	3.17	1.44	2.06
H07	H06R	11.4	1	11.22	0.18	0.03	11.21	0.19	0.04	11.20	0.20	0.04	11.22	0.18	0.03
H07	H08R	4.7	1	4.30	0.40	0.16	4.30	0.40	0.16	4.29	0.41	0.17	4.30	0.40	0.16
H08S	H09	11.1	1	11.02	0.08	0.01	10.98	0.12	0.01	10.95	0.15	0.02	10.95	0.15	0.02

H08R	H09	1.6	1	2.80	1.20	1.45	2.81	1.21	1.46	2.87	1.27	1.60	2.80	1.20	1.45
H05	H04R	10.9	1	10.05	0.85	0.72	9.84	1.06	1.13	9.65	1.25	1.56	9.89	1.02	1.03
H05	H04S	2.2	1	2.85	0.65	0.42	2.75	0.55	0.30	2.69	0.49	0.24	2.62	0.42	0.18
H05	H06S	2.2	1	2.40	0.20	0.04	2.44	0.24	0.06	2.45	0.25	0.06	2.41	0.21	0.04
H05	H06R	11.3	1	11.40	0.10	0.01	11.37	0.07	0.01	11.29	0.01	0.00	11.36	0.06	0.00
H22R	H23	2	1	2.00	0.00	0.00	2.04	0.04	0.00	2.66	0.66	0.44	2.70	0.70	0.49
H22R	H21	9.1	1	9.86	0.76	0.58	9.44	0.34	0.12	8.41	0.69	0.47	8.40	0.70	0.49
H52	H51	1.4	1	2.24	0.84	0.70	2.32	0.92	0.84	2.20	0.80	0.64	2.32	0.92	0.84
H52	H53	3.8	1	3.31	0.49	0.24	3.29	0.51	0.26	3.29	0.51	0.26	3.29	0.52	0.27
H53	H54	9.6	1	9.77	0.17	0.03	9.61	0.01	0.00	9.81	0.21	0.05	9.60	0.00	0.00
H55	H56b	6.2	1	6.21	0.01	0.00	6.21	0.01	0.00	6.21	0.01	0.00	6.20	0.00	0.00
H55	H56c	6.2	1	6.22	0.02	0.00	6.20	0.00	0.00	6.21	0.01	0.00	6.20	0.00	0.00
H55	H56a	6.2	1	6.20	0.00	0.00	6.20	0.00	0.00	6.21	0.00	0.00	6.20	0.00	0.00
H55	H54	9.4	1	9.14	0.26	0.07	9.17	0.23	0.05	9.34	0.06	0.00	9.14	0.26	0.07
H20	H21	8	1	8.27	0.27	0.07	8.00	0.00	0.00	8.10	0.10	0.01	8.04	0.04	0.00
H22S	H23	11.7	1	10.98	0.72	0.52	10.99	0.71	0.50	9.60	2.11	4.43	9.57	2.13	4.55
H22S	H21	2	1	2.47	0.47	0.22	2.52	0.52	0.27	3.41	1.41	1.99	3.27	1.27	1.61
H23	H24R	5	1	5.37	0.37	0.13	5.25	0.25	0.06	5.04	0.04	0.00	5.04	0.04	0.00
H23	H24S	3.3	1	4.13	0.83	0.69	4.08	0.78	0.61	3.75	0.45	0.20	3.73	0.43	0.18
H25c	H11	6.8	1	6.53	0.27	0.08	6.56	0.24	0.06	6.55	0.25	0.06	6.55	0.25	0.06
H25b	H11	6.8	1	6.53	0.27	0.07	6.54	0.26	0.07	6.55	0.26	0.07	6.56	0.24	0.06
H25a	H11	6.8	1	6.52	0.28	0.08	6.55	0.25	0.06	6.55	0.25	0.06	6.56	0.24	0.06
H18	H26c	7	1	6.57	0.43	0.18	6.62	0.39	0.15	6.60	0.40	0.16	6.61	0.39	0.15
H18	H26b	7	1	6.57	0.43	0.19	6.61	0.39	0.15	6.60	0.40	0.16	6.60	0.40	0.16
H18	H26a	7	1	6.56	0.44	0.19	6.61	0.39	0.15	6.60	0.40	0.16	6.59	0.41	0.17

**n**	38	**n**	38	**n**	38	**n**	38
**outliers**	3	**outliers**	4	**outliers**	7	**outliers**	6
**F**	35/38	**F**	34/38	**F**	31/38	**F**	32/38
**RMSD**	0.64	**RMSD**	0.58	**RMSD**	0.78	**RMSD**	0.71
**min(1/X2)**	0.22	**min(1/X2)**	0.49	**min(1/X2)**	0.23	**min(1/X2)**	0.22
**n/X2**	2.42	**n/X2**	2.97	**n/X2**	1.65	**n/X2**	1.98

Table S26: Experimental and calculated NOE distances for Mandelalide A configuration 11-epi-**2*p***. The error of the experiment estimated to 0.5 Å. NOE distances to OH-groups are not taken as constraints. Values in red are outside the experimental estimated error margins ($\chi 2>1$).

NOEs															
expDATA				MDOC: 2p			MDOC: 11-epi-2p			MDOC: 2r			MDOC: 11-epi-2r		
Atom A	Atom B	NOE Dist. Exp. / Å	Err. / Å	NOE Dist. Calc. / Å	Diff. Exp.-Calc. / Å	χ2	NOE Dist. Calc. / Å	Diff. Exp.-Calc. / Å	χ2	NOE Dist. Calc. / Å	Diff. Exp.-Calc. / Å	χ2	NOE Dist. Calc. / Å	Diff. Exp.-Calc. / Å	χ2
H17	H16R	2.56	0.5	2.65	0.09	0.03	2.61	0.04	0.01	2.70	0.14	0.08	2.69	0.13	0.07
H17	H16S	2.59	0.5	2.63	0.04	0.01	2.57	0.02	0.00	2.45	0.14	0.08	2.36	0.23	0.20
H17	H15	2.36	0.5	2.67	0.31	0.38	2.73	0.37	0.54	2.83	0.47	0.88	2.90	0.54	1.15
H17	H18	2.07	0.5	2.33	0.26	0.26	2.31	0.24	0.23	2.33	0.26	0.27	2.32	0.25	0.25
H17	H26a	3.63	0.5	3.29	0.34	0.45	3.29	0.34	0.45	3.46	0.17	0.11	3.50	0.13	0.06
H17	H26b	3.63	0.5	3.30	0.34	0.45	3.30	0.33	0.44	3.46	0.17	0.12	3.49	0.14	0.08
H17	H26c	3.63	0.5	3.27	0.36	0.53	3.29	0.34	0.47	3.46	0.17	0.11	3.50	0.13	0.07
H19S	H16S	2.85	0.5	2.67	0.18	0.14	2.66	0.19	0.15	4.11	1.26	6.39	4.20	1.35	7.24
H19S	H20	2.87	0.5	2.94	0.07	0.02	2.92	0.05	0.01	2.35	0.52	1.10	2.35	0.52	1.09

H19S	H22S	2.7	0.5	2.54	0.16	0.10	2.54	0.16	0.10	3.30	0.60	1.42	3.30	0.60	1.42
H19S	H21	2.34	0.5	2.60	0.26	0.27	2.60	0.26	0.28	3.28	0.94	3.53	3.24	0.90	3.21
H19S	H18	3.36	0.5	2.94	0.43	0.72	2.92	0.44	0.79	2.35	1.01	4.05	2.35	1.01	4.09
H19S	H26a	3.08	0.5	2.93	0.15	0.09	2.93	0.15	0.09	3.28	0.20	0.16	3.28	0.20	0.16
H19S	H26b	3.08	0.5	2.92	0.16	0.11	2.92	0.16	0.11	3.28	0.20	0.15	3.29	0.21	0.17
H19S	H26c	3.08	0.5	2.94	0.14	0.07	2.92	0.16	0.10	3.27	0.19	0.14	3.28	0.20	0.16
H19R	H20	2.26	0.5	2.37	0.11	0.05	2.36	0.10	0.04	2.85	0.59	1.38	2.85	0.59	1.40
H19R	H22S	2.94	0.5	2.87	0.07	0.02	2.93	0.01	0.00	2.94	0.00	0.00	2.93	0.01	0.00
H16R	H15	2.47	0.5	2.88	0.41	0.67	2.95	0.48	0.91	2.89	0.42	0.69	2.92	0.45	0.80
H16R	H13	2.76	0.5	2.17	0.59	1.39	2.08	0.68	1.86	2.10	0.66	1.72	2.08	0.68	1.83
H16R	H25c	2.68	0.5	3.25	0.57	1.31	3.28	0.60	1.43	3.27	0.59	1.39	3.20	0.52	1.09
H16R	H25b	2.68	0.5	3.27	0.59	1.38	3.28	0.60	1.44	3.26	0.58	1.33	3.21	0.53	1.12
H16R	H25a	2.68	0.5	3.25	0.57	1.31	3.28	0.60	1.44	3.26	0.58	1.33	3.21	0.53	1.12
H16R	H26a	2.68	0.5	2.88	0.20	0.17	2.92	0.24	0.23	2.86	0.18	0.13	2.83	0.15	0.08
H16R	H26b	2.68	0.5	2.85	0.17	0.11	2.91	0.23	0.21	2.86	0.18	0.13	2.84	0.16	0.10
H16R	H26c	2.68	0.5	2.87	0.19	0.15	2.92	0.24	0.23	2.85	0.17	0.11	2.83	0.15	0.09
H16S	H15	3.03	0.5	2.70	0.33	0.43	2.60	0.43	0.74	2.57	0.46	0.84	2.49	0.54	1.18
H16S	H13	2.2	0.5	2.27	0.07	0.02	2.45	0.25	0.24	2.31	0.11	0.05	2.49	0.29	0.33
H16S	H21	2.94	0.5	2.47	0.47	0.88	2.51	0.43	0.75	2.84	0.10	0.04	3.11	0.17	0.11
H14	H12	2.11	0.5	2.70	0.59	1.39	2.63	0.52	1.10	2.73	0.62	1.56	2.69	0.58	1.36
H13	H09	3.15	0.5	3.38	0.23	0.21	3.28	0.13	0.07	3.13	0.02	0.00	3.13	0.02	0.00
H13	H05	4.09	0.5	4.00	0.09	0.04	3.93	0.16	0.10	4.08	0.01	0.00	3.81	0.28	0.31
H13	H03	3.08	0.5	3.54	0.46	0.83	3.48	0.40	0.63	3.68	0.60	1.44	3.62	0.54	1.18
H13	H21	2.91	0.5	3.37	0.46	0.83	3.32	0.41	0.67	3.32	0.41	0.68	3.14	0.23	0.20
H13	H11	2.21	0.5	2.38	0.17	0.12	2.50	0.29	0.34	2.42	0.21	0.18	2.41	0.20	0.16
H12	H10R	2.67	0.5	2.93	0.26	0.27	2.83	0.16	0.11	2.91	0.24	0.24	2.74	0.07	0.02
H12	H09	2.97	0.5	3.22	0.25	0.25	2.90	0.07	0.02	3.12	0.15	0.09	2.76	0.21	0.17
H12	H25c	2.8	0.5	3.14	0.34	0.45	3.17	0.37	0.55	3.12	0.32	0.40	3.13	0.33	0.43
H12	H25b	2.8	0.5	3.13	0.33	0.43	3.17	0.37	0.55	3.11	0.31	0.38	3.13	0.33	0.43
H12	H25a	2.8	0.5	3.12	0.32	0.40	3.17	0.37	0.54	3.11	0.31	0.39	3.13	0.33	0.43
H12	H11	2.97	0.5	2.67	0.30	0.36	2.62	0.35	0.48	2.62	0.35	0.48	2.73	0.24	0.22
H10S	H08S	2.97	0.5	2.67	0.30	0.36	2.66	0.31	0.39	2.64	0.33	0.45	2.66	0.32	0.40
H10S	H25c	2.95	0.5	3.14	0.19	0.14	3.02	0.07	0.02	3.14	0.19	0.15	2.97	0.02	0.00
H10S	H25b	2.95	0.5	3.16	0.21	0.17	3.02	0.07	0.02	3.14	0.19	0.14	2.97	0.02	0.00
H10S	H25a	2.95	0.5	3.15	0.20	0.16	3.02	0.07	0.02	3.14	0.19	0.15	2.97	0.01	0.00
H10S	H11	2.57	0.5	2.53	0.04	0.01	2.53	0.04	0.01	2.50	0.07	0.02	2.54	0.03	0.00
H10R	H08S	2.9	0.5	2.98	0.08	0.03	3.08	0.18	0.13	3.02	0.12	0.06	3.06	0.16	0.10
H10R	H08R	2.54	0.5	2.77	0.23	0.20	2.64	0.10	0.04	2.60	0.06	0.02	2.59	0.05	0.01
H10R	H09	2.42	0.5	2.49	0.07	0.02	2.53	0.11	0.04	2.54	0.12	0.06	2.53	0.11	0.04
H07	H08R	2.39	0.5	2.63	0.24	0.22	2.50	0.11	0.05	2.50	0.11	0.05	2.50	0.11	0.04
H07	H09	2.43	0.5	2.93	0.50	0.99	2.57	0.14	0.08	2.60	0.17	0.11	2.58	0.15	0.09
H07	H05	2.7	0.5	2.98	0.28	0.32	2.61	0.09	0.03	2.59	0.11	0.05	2.59	0.11	0.05
H07	H06S	2.37	0.5	2.45	0.07	0.02	2.44	0.07	0.02	2.44	0.07	0.02	2.44	0.07	0.02
H07	H51	2.23	0.5	2.74	0.51	1.04	2.64	0.41	0.68	2.64	0.41	0.67	2.64	0.41	0.66
H08S	H06R	2.9	0.5	2.98	0.08	0.03	2.61	0.29	0.35	2.60	0.30	0.36	2.61	0.29	0.34
H08R	H09	2.66	0.5	2.52	0.14	0.08	2.52	0.14	0.08	2.53	0.13	0.07	2.53	0.13	0.07
H08R	H55	2.86	0.5	3.39	0.53	1.13	3.06	0.20	0.16	3.04	0.18	0.13	3.05	0.19	0.15
H08R	H51	3.72	0.5	2.91	0.81	2.60	3.05	0.68	1.82	3.04	0.68	1.87	3.02	0.70	1.95
H09	H05	2.18	0.5	2.47	0.29	0.33	2.34	0.16	0.10	2.34	0.16	0.10	2.31	0.13	0.07
H09	H26a	4.95	0.5	4.96	0.00	0.00	4.95	0.00	0.00	5.25	0.30	0.35	5.28	0.33	0.43

H09	H26b	4.95	0.5	5.00	0.05	0.01	4.96	0.01	0.00	5.26	0.31	0.39	5.26	0.31	0.38
H09	H26c	4.95	0.5	4.97	0.02	0.00	4.97	0.02	0.00	5.26	0.31	0.39	5.28	0.33	0.44
H05	H06S	2.42	0.5	2.57	0.15	0.09	2.45	0.02	0.00	2.43	0.01	0.00	2.44	0.02	0.00
H05	H04S	2.5	0.5	2.52	0.02	0.00	2.51	0.01	0.00	2.51	0.01	0.00	2.51	0.01	0.00
H05	H03	2.76	0.5	2.86	0.10	0.04	2.83	0.07	0.02	2.77	0.01	0.00	2.73	0.03	0.00
H05	H25c	4.7	0.5	4.14	0.56	1.25	4.63	0.07	0.02	4.20	0.50	0.98	4.72	0.02	0.00
H05	H25b	4.7	0.5	4.15	0.55	1.22	4.62	0.08	0.02	4.21	0.49	0.95	4.73	0.03	0.00
H05	H25a	4.7	0.5	4.19	0.51	1.05	4.64	0.06	0.01	4.19	0.51	1.02	4.76	0.06	0.02
H06R	H04R	2.71	0.5	3.07	0.36	0.53	2.66	0.05	0.01	2.69	0.02	0.00	2.67	0.04	0.01
H06R	H51	3.54	0.5	2.86	0.68	1.87	2.94	0.60	1.45	2.94	0.60	1.44	2.96	0.58	1.35
H06S	H04S	2.83	0.5	2.73	0.10	0.04	2.68	0.16	0.10	2.67	0.17	0.11	2.60	0.23	0.22
H06S	H51	2.56	0.5	2.89	0.33	0.43	2.67	0.11	0.05	2.67	0.11	0.05	2.67	0.11	0.05
H04S	H03	2.64	0.5	2.74	0.10	0.04	2.75	0.11	0.05	2.76	0.12	0.06	2.80	0.16	0.11
H04S	H02	2.68	0.5	2.69	0.01	0.00	2.65	0.03	0.00	2.59	0.09	0.03	2.56	0.12	0.05
H04R	H03	2.69	0.5	2.71	0.02	0.00	2.67	0.02	0.00	2.70	0.01	0.00	2.70	0.01	0.00
H04R	H02	2.71	0.5	2.76	0.05	0.01	2.75	0.04	0.01	2.77	0.06	0.02	2.77	0.06	0.01
H22R	H20	2.29	0.5	2.70	0.41	0.68	2.70	0.41	0.68	2.82	0.53	1.13	2.82	0.53	1.12
H22R	H23	2.44	0.5	2.50	0.06	0.01	2.48	0.04	0.01	2.58	0.14	0.08	2.58	0.14	0.08
H22R	H24R	2.83	0.5	2.92	0.09	0.03	2.92	0.09	0.03	3.12	0.29	0.33	3.13	0.30	0.35
H22R	H21	3.04	0.5	2.84	0.21	0.17	2.84	0.20	0.16	2.50	0.54	1.15	2.52	0.52	1.08
H03	H21	3.09	0.5	3.16	0.07	0.02	3.16	0.07	0.02	3.27	0.18	0.13	3.24	0.15	0.09
H03	H11	3.01	0.5	3.25	0.24	0.23	3.35	0.34	0.47	3.51	0.50	0.98	3.47	0.46	0.86
H52	H53	2.24	0.5	2.44	0.20	0.16	2.42	0.18	0.12	2.42	0.18	0.13	2.42	0.18	0.13
H52	H51	2.3	0.5	2.69	0.39	0.62	2.54	0.24	0.24	2.55	0.25	0.24	2.54	0.24	0.24
H55	H56a	2.49	0.5	2.55	0.06	0.02	2.57	0.08	0.03	2.57	0.08	0.03	2.57	0.08	0.03
H55	H56c	2.49	0.5	2.55	0.06	0.02	2.57	0.08	0.03	2.57	0.08	0.03	2.58	0.09	0.03
H55	H56b	2.49	0.5	2.56	0.06	0.02	2.57	0.08	0.03	2.57	0.08	0.03	2.57	0.08	0.03
H55	H51	3.88	0.5	3.05	0.84	2.79	3.66	0.22	0.20	3.66	0.22	0.20	3.66	0.22	0.19
H56a	H54	2.73	0.5	2.99	0.26	0.26	2.86	0.13	0.07	2.86	0.13	0.07	2.86	0.13	0.07
H56c	H54	2.73	0.5	2.98	0.25	0.25	2.86	0.13	0.07	2.86	0.13	0.07	2.86	0.13	0.07
H56b	H54	2.73	0.5	2.97	0.24	0.24	2.86	0.13	0.07	2.86	0.13	0.07	2.86	0.13	0.07
H51	H57c	2.74	0.5	2.88	0.14	0.08	2.81	0.07	0.02	2.78	0.04	0.01	2.85	0.11	0.04
H51	H57a	2.74	0.5	2.87	0.13	0.07	2.81	0.07	0.02	2.78	0.04	0.01	2.84	0.10	0.04
H51	H57b	2.74	0.5	2.88	0.14	0.08	2.81	0.07	0.02	2.77	0.03	0.00	2.84	0.10	0.04
H20	H18	2.58	0.5	2.73	0.15	0.09	2.76	0.18	0.13	2.64	0.06	0.01	2.63	0.04	0.01
H22S	H24R	3.24	0.5	2.90	0.34	0.47	2.93	0.31	0.39	3.03	0.22	0.18	3.00	0.24	0.22
H22S	H21	2.47	0.5	2.54	0.07	0.02	2.55	0.08	0.03	2.64	0.17	0.12	2.63	0.16	0.10
H23	H24S	2.24	0.5	2.55	0.31	0.37	2.55	0.31	0.39	2.55	0.31	0.38	2.55	0.31	0.38
H23	H24R	2.39	0.5	2.66	0.27	0.30	2.59	0.20	0.16	2.56	0.17	0.12	2.56	0.17	0.12
H23	H21	2.67	0.5	2.92	0.25	0.24	2.87	0.20	0.16	2.91	0.24	0.23	2.92	0.25	0.25
H21	H18	3.4	0.5	4.34	0.94	3.56	4.30	0.90	3.23	4.23	0.83	2.78	4.23	0.83	2.74
H25c	H11	2.3	0.5	2.52	0.22	0.19	2.52	0.22	0.19	2.52	0.22	0.20	2.53	0.23	0.20
H25b	H11	2.3	0.5	2.53	0.23	0.21	2.52	0.22	0.19	2.52	0.22	0.20	2.53	0.23	0.20
H25a	H11	2.3	0.5	2.52	0.22	0.20	2.52	0.22	0.19	2.52	0.22	0.20	2.53	0.23	0.20
H18	H26a	2.3	0.5	2.51	0.21	0.18	2.51	0.21	0.18	2.51	0.21	0.18	2.51	0.21	0.18
H18	H26b	2.3	0.5	2.53	0.23	0.21	2.51	0.21	0.18	2.51	0.21	0.18	2.51	0.21	0.18
H18	H26c	2.3	0.5	2.52	0.22	0.19	2.52	0.22	0.18	2.52	0.22	0.19	2.52	0.22	0.18
				**n**		**106**	**n**		**106**	**n**		**106**	**n**		**106**
				**outliers**		**14**	**outliers**		**8**	**outliers**		**18**	**outliers**		**19**
				**F**		**92/106**	**F**		**98/106**	**F**		**88/106**	**F**		**87/106**

RMSD	0.32	RMSD	0.28	RMSD	0.36	RMSD	0.34
min(1/X2)	0.28	min(1/X2)	0.31	min(1/X2)	0.16	min(1/X2)	0.14
n/X2	2.44	n/X2	3.30	n/X2	1.98	n/X2	2.11

Table S27: Summary of fitting scores (#outliers, Fidelity, min(1/χ^2, n/ χ^2) for the ensemble of data(Table Table S24, Table S25, and Table S26) used in the MDOC simulation of Mandelalide A configuration 11-*epi*-**2*p*.**

Mandelalide A 11-*epi*-**2*p***

MDOC: 2p		MDOC: 11-epi-2p		MDOC: 2r		MDOC: 11-epi-2r	
n	192	n	192	n	192	n	192
outliers	19	outliers	13	outliers	29	outliers	26
F	173/192	F	179/192	F	163/192	F	166/192
min(1/X2)	0.22	min(1/X2)	0.31	min(1/X2)	0.16	min(1/X2)	0.14
n/X2	2.77	n/X2	3.57	n/X2	2.19	n/X2	2.49

4 Structure ensembles

4.1 Mandelalide A

4.1.1 Mandelalide A 2p

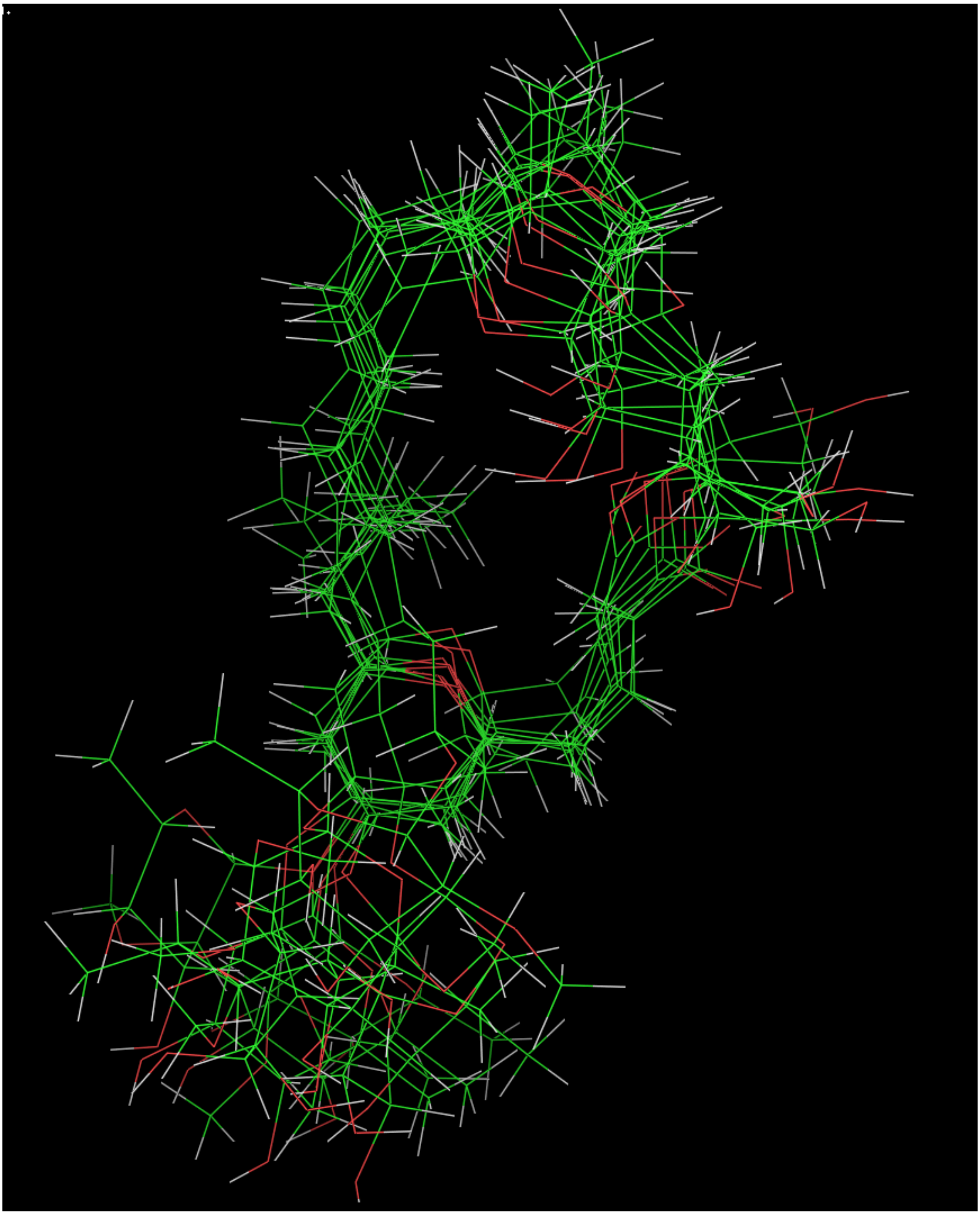

Figure S4: Overlay of 10 representative structures from the MDOC ensemble end result of mandelalide A **2p** run with dataset 2p. These structures are also available from: https://dx.doi.org/10.17617/3.34

4.1.2 Mandelalide A 11-epi-2A

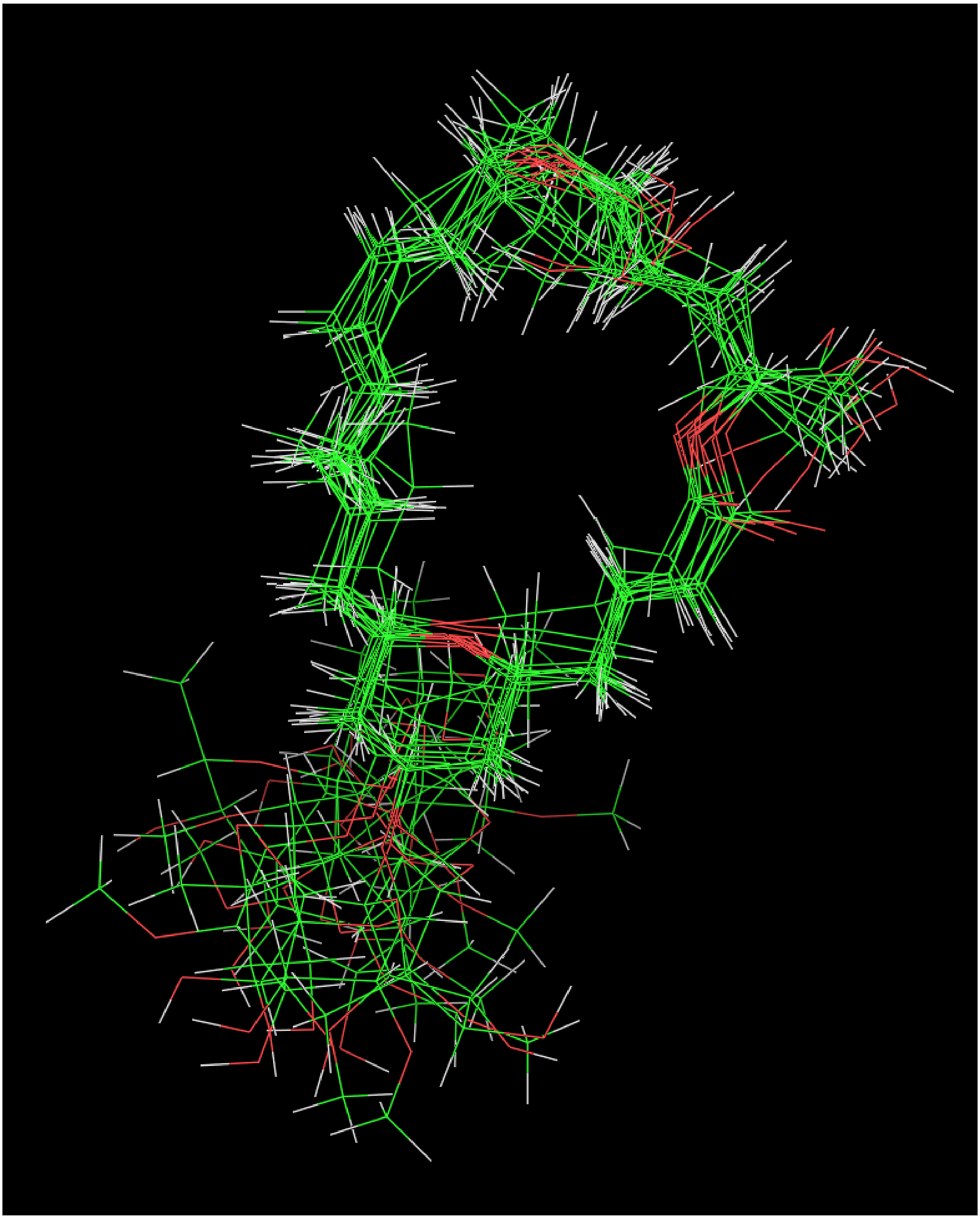

Figure S5: Overlay of 10 representative structures from the MDOC ensemble end result of mandelalide A 11-*epi*-**2*p*** run with dataset **2*p***. These structures are also available from: https://dx.doi.org/10.17617/3.34

5 dPCA results for mandelalide A *11-epi-***2*p***

"Nr"	"Name 1"	"Nr1"	"Nr2"	"Nr3"	"Nr4"	"Name 4"
4	"C17"	1	2	3	4	"C20"
5	"C18"	2	3	4	74	"O110"
6	"C18"	2	3	4	39	"C21"
7	"C18"	2	1	74	4	"C20"
8	"C18"	2	1	8	12	"C15"
9	"C19"	3	4	39	41	"C22"
10	"C19"	3	2	1	74	"O110"
13	"C20"	4	39	41	77	"C23"
14	"C16"	8	12	11	16	"C13"
15	"C14"	11	16	15	19	"C11"
16	"C15"	12	11	16	15	"C12"
18	"C12"	15	19	22	25	"C09"
19	"C13"	16	15	19	22	"C10"
21	"C11"	19	22	25	24	"C08"
23	"C10"	22	25	24	23	"C07"
26	"C07"	23	28	27	36	"C04"
29	"C08"	24	25	26	27	"C05"
35	"C09"	25	24	23	28	"C06"
37	"C05"	27	36	40	72	"C02"
41	"C04"	36	40	72	44	"C01"
42	"C21"	39	41	77	43	"O101"
43	"C03"	40	72	44	43	"O101"
44	"C22"	41	77	43	44	"C01"
54	"C02"	72	44	43	77	"C23"

Table S 28: List of dihedral angles used in the dPCA analysis of 11-*epi*-**2*p***

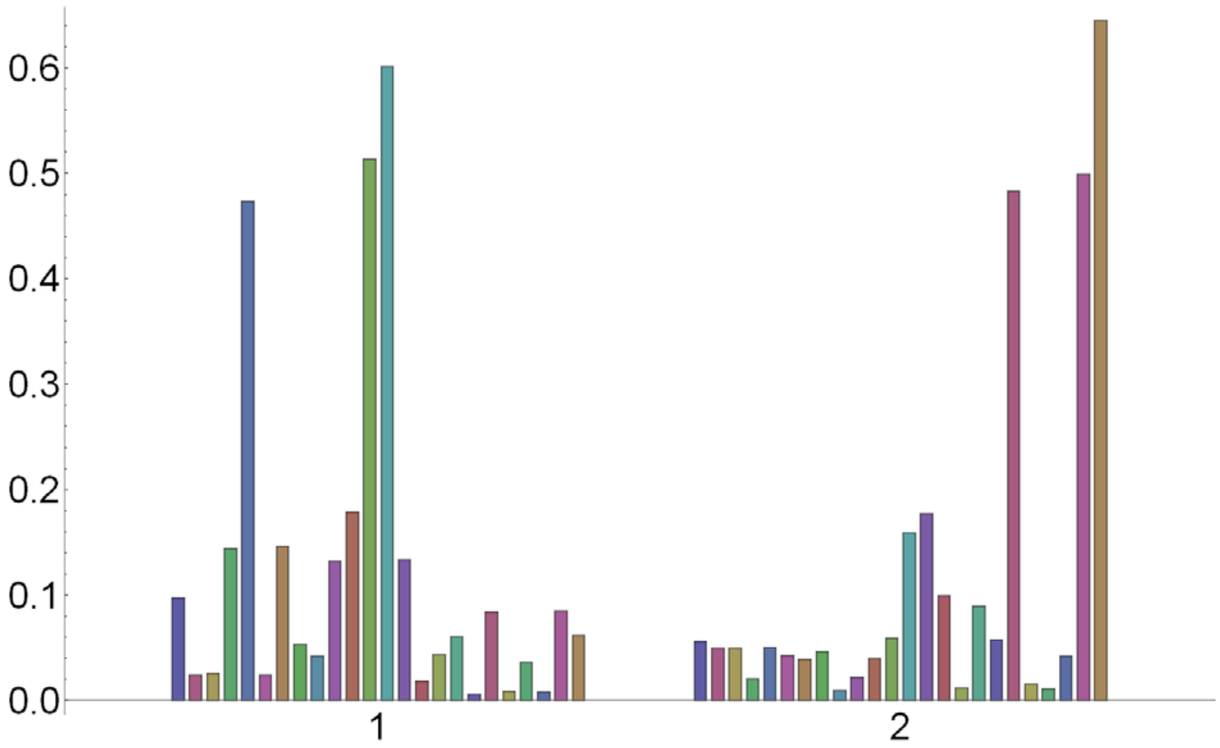

Figure S6: Relative contribution of the dihedrals (see Table S28) to the first 2 principal components for the dPCA analysis of the MDOC simulation of 11-*epi*-**2*p***

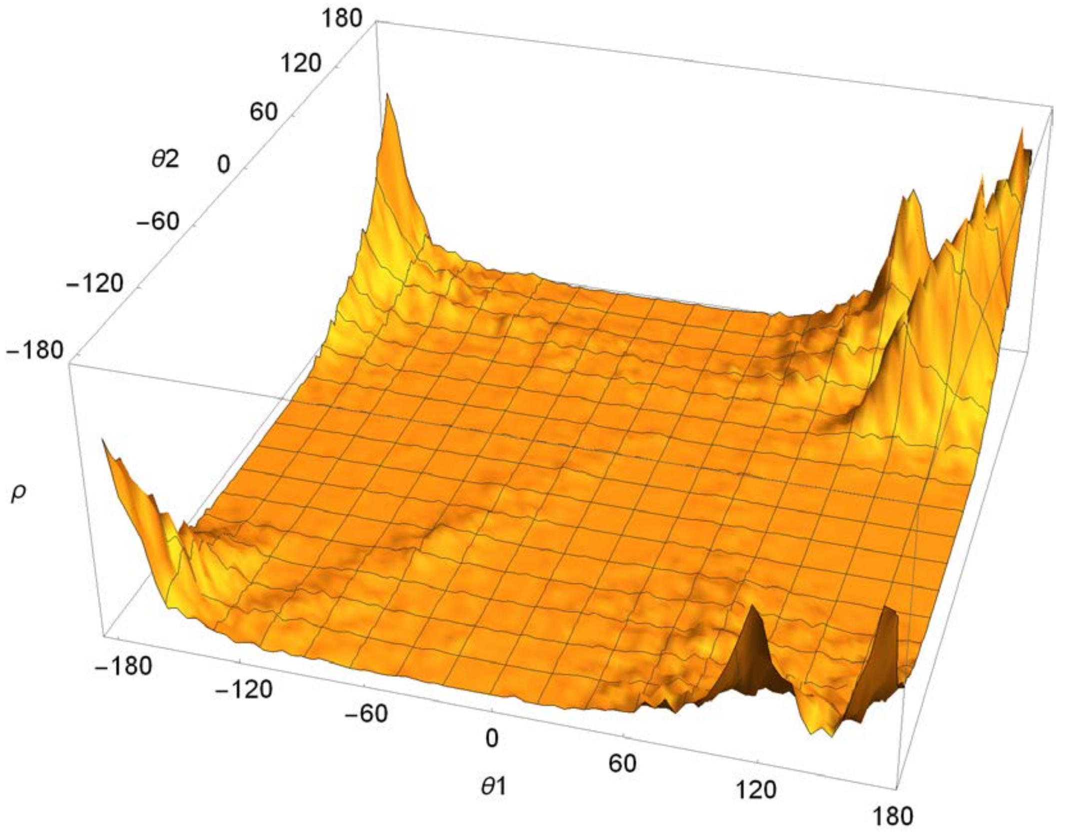

Figure S7: 2D dihedral landscape of the first two principal components of the mandelalide A isomer 11-*epi*-**2*p***

6 References

1. Pupier, M.; Nuzillard, J.M.; Wist, J.; Schlorer, N.E.; Kuhn, S.; Erdelyi, M.; Steinbeck, C.; Williams, A.J.; Butts, C.; Claridge, T.D.W., et al. NMReDATA, a standard to report the NMR assignment and parameters of organic compounds. *Magn. Reson. Chem.* **2018**, *56*, 703-715, doi:10.1002/mrc.4737.
2. Altis, A.; Nguyen, P.H.; Hegger, R.; Stock, G. Dihedral angle principal component analysis of molecular dynamics simulations. *J. Chem. Phys.* **2007**, *126*, doi:10.1063/1.2746330.
3. Altis, A.; Otten, M.; Nguyen, P.H.; Hegger, R.; Stock, G. Construction of the free energy landscape of biomolecules via dihedral angle principal component analysis. *J. Chem. Phys.* **2008**, *128*, doi:10.1063/1.2945165.
4. Palermo, G.; Riccio, R.; Bifulco, G. Effect of Electronegative Substituents and Angular Dependence on the Heteronuclear Spin-Spin Coupling Constant (3)J(C-H): An Empirical Prediction Equation Derived by Density Functional Theory Calculations. *Journal of Organic Chemistry* **2010**, *75*, 1982-1991, doi:10.1021/jo902704u.
5. Haasnoot, C.A.G.; Deleeuw, F.A.A.M.; Altona, C. The Relationship between Proton-Proton Nmr Coupling-Constants and Substituent Electronegativities .1. An Empirical Generalization of the Karplus Equation. *Tetrahedron* **1980**, *36*, 2783-2792, doi:10.1016/0040-4020(80)80155-4.
